# Towards Metabolic Organic Radical Contrast Agents (mORCAs) for Magnetic Resonance Imaging

**DOI:** 10.3390/molecules30071581

**Published:** 2025-04-02

**Authors:** Shuyang Zhang, Sabina Dhakal, Evan Curtis, Hunter Miller, Joseph T. Paletta, Connor Gee, Suchada Rajca, Forrest Kievit, Andrzej Rajca

**Affiliations:** 1 Department of Chemistry, University of Nebraska, Lincoln, NE 68588-0304, USA; zhangshuyangyili@gmail.com (S.Z.); sdhakal6@huskers.unl.edu (S.D.); joepaletta21@gmail.com (J.T.P.); srajca.chem@gmail.com (S.R.); 2 Department of Biological Systems Engineering, University of Nebraska, Lincoln, NE 68583-0900, USA; ecurtis@huskers.unl.edu (E.C.); hmiller23@unl.edu (H.M.); connor.gee@huskers.unl.edu (C.G.); fkievit2@unl.edu (F.K.)

**Keywords:** nitroxide radical, mannosamine, metabolic glycan engineering, electron paramagnetic resonance, magnetic resonance imaging

## Abstract

We report two conjugates of *gem*-diethyl pyrroline nitroxide radicals with D-mannosamine as potential metabolic organic radical contrast agents, mORCAs, circumventing the need for biorthogonal reactions. In-cell EPR spectroscopy, using Jurkat cells and analogous conjugate, based on a pyrrolidine nitroxide radical, shows an efficient incorporation of highly immobilized nitroxides, with a correlation time of *τ*_cor_ = 20 ns. In vivo MRI experiments in mice show that the paramagnetic nitroxide radical shortens the *T*_1_ and *T*_2_ relaxation times of protons in water located in the kidney and brain by only up to ~10% after 3 d. Ex vivo EPR spectroscopic analyses indicate that the contrast agents in mouse tissues are primarily localized in the kidney, lung, liver, heart, and blood, which primarily contain immobilized nitroxide radicals with *τ*_cor_ = 4–9 ns. The spin concentrations in tissues remain low (1–3 nmol g⁻^1^) at 24 h after the third mORCA injection, approximately one to two orders of magnitude lower than those of ORCAFluor and BASP-ORCA (measured at ~24 h post-injection). These low spin concentrations explain the small proton *T*_1_ and *T*_2_ relaxation changes observed in in vivo MRI.

## 1. Introduction

Organic radical contrast agents (ORCAs) for magnetic resonance imaging (MRI) are water-soluble macromolecules functionalized with stable organic radicals, such as nitroxide radicals. Although nitroxide radicals have a long history as contrast agents for MRI (and probes for EPRI) [1,2,3,4,5], the important milestone was achieved by the implementation of the first ORCA in 2012, which demonstrated the long-lasting MRI images of mice [6], as highlighted by the three review articles on the subject in the past few months [7,8,9].

**Figure 1:**
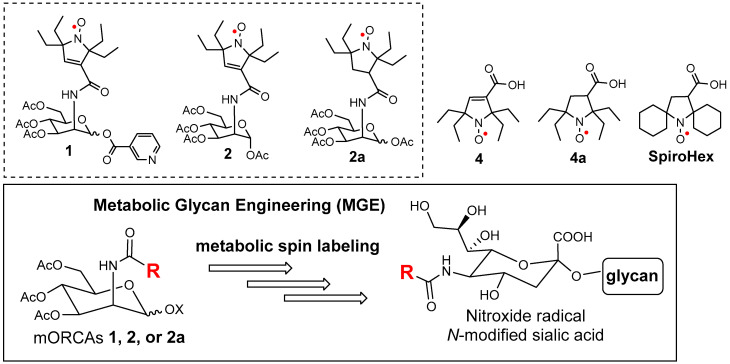
mORCAs **1**, **2**, and **2a** to be used in metabolic spin labeling via glycan engineering and selected nitroxide radicals, such as the *gem*-diethyl pyrroline **4** and pyrrolidine **4a** nitroxides, as well as the bis-spirocyclohexyl pyrrolidine nitroxide radical **SpiroHex**.

The main focus of our research has been on the development of ORCAs based on the bis-spirocyclohexyl pyrrolidine nitroxide radical **SpiroHex** (Figure 1). These ORCAs provide a superior combination of resistance to reduction in vivo and high relaxivity, termed *r*_1_ and *r*_2_, thus leading to long-lasting and strongly enhanced images in vivo [6,10]. The brush-arm star polymer ORCA (BASP-ORCA) was designed to leverage the molecular architecture of strongly immobilized nitroxide radicals (with a rotational correlation time of *τ*_cor_ ≈ 10 ns) to achieve the value of *r*_2_ per *S* = ½ radical, which is comparable to clinical *S* = 7/2 Gd(III)-based contrast agents, thus allowing for high-resolution MRI of tumors in mice [11,12].

Inspired by the discovery of unusually slow reduction of various *gem*-diethyl 5-membered ring nitroxide radicals with ascorbate by Rassat and others [13,14], we have synthesized and studied the *gem*-diethyl pyrroline **4** and pyrrolidine **4a** nitroxides (Figure 1) and their derivatives [15,16]. It has been found that **4**, **4a**, and their derivatives are among the least susceptible organic radicals to reduction by ascorbate, as well as to reduction in live cells and cell or organ homogenates [15,16,17,18,19,20,21]. Moreover, various stable and reduction-resistant pyrroline, pyrrolidine, and piperidine nitroxide radicals have expanded their potential in EPR and NMR spectroscopy, as well as biomedical imaging [22,23,24,25,26].

As we continue pushing the frontiers of our ORCA development, we reckon the difficult challenges. While macromolecular ORCAs, such as dendrimers and various brush polymer architectures, have provided an exciting feasibility for an effective organic MRI contrast agent, there are challenges associated with difficulties in synthesis, reproducibility, large-scale production (consistent size, shape, and drug loading), biocompatibility, and potential immune responses. Meanwhile, small molecule nitroxides as contrast agents are ineffective due to their short half-life as well as rapid clearance.

We have conjectured a concept of metabolic spin labeling that leads to a metabolic ORCA (mORCA). We speculated that nitroxide radical-conjugated *O*-acetylated *D*-mannosamine, e.g., 1, **2**, and **2a** (Figure 1), in which the nitroxide radicals such as **4** and **4a** are difficult to reduce, might be employed as metabolic glycan engineering (MGE) substrates to provide nitroxide radicals on the cell surface, i.e., metabolic spin labeling in one step, thus bypassing the bioorthogonal reaction. The two-step bioorthogonal approach, while powerful, is not without limitations. The use of chemical handles like azides, tetrazines, or thiols can lead to unintended side reactions due to their reactivity with endogenous molecules (e.g., phosphines, amines, or thiols). These side reactions are exacerbated by the need for excess reagents and the extended exposure times required in biological systems [27]. As a result, alternative strategies, such as direct metabolic labeling, may be necessary to achieve higher specificity and efficiency in complex biological environments.

The mORCA might also be more effective for tumor detection, compared to the macromolecular ORCAs based on enhanced permeability and the retention effect. Furthermore, the mORCA could be more readily modified with specific functional groups for targeting. For example, the uptake of the nicotinic acid derivative **1** by the brain may possibly be enhanced due to the presence of nicotinic receptors in the blood–brain barrier (BBB) [28].

MGE is an established method for labeling/modifying glycan structures in cells by treating cells with unnatural derivatives of monosaccharides. It has been established by Bertozzi and others that *O*-acetylated *D*-mannosamines functionalized with small *N*-substituents, in particular **R** = CH_2_N_3_, methyl azide (Figure 1), provide superior substrates for MGE, enabling the modification of the glycans (glycoproteins) via bioorthogonal reactions of the sialic acids on the cell surface [29,30,31,32,33,34,35,36,37]. The technique has been effectively employed to incorporate a variety of reporters, such as fluorescent dyes, Gd(III)-contrast agents, drugs, nitroxide radical, or other agents, onto the cell surface [37,38,39,40,41,42]. In particular, the nitroxide radicals were incorporated into the sialic acids in cells by using classic *O*-acetylated *D*-mannosamine with **R** = CH_2_N_3_, followed by the biorthogonal click reaction with strained alkyne [37], functionalized with the *gem*-dimethyl pyrroline nitroxide radical, based on qualitative EPR spectroscopic evidence [42].

Because of the relatively small size of the nitroxide radical, mORCAs have promising potential to achieve direct metabolic spin labeling, thereby attaching nitroxide radicals to the cell surface. The outcome could be evaluated by quantitative ex vivo EPR spectroscopy and in vivo MRI, e.g., the degree of nitroxide immobilization, spin concentration, and lowering proton *T*_1_ or *T*_2_ in mouse tissues. We recognize that these measurements rely on the presence of intact radical in tissues—a tall order because of the slow rate of MGE, requiring radicals with resistance to reduction on the order of days in a living mammal. We note that the C_12_ nitroxide radicals **4** and **4a** possess significantly larger molecular size and degree of branching, compared to the largest **R** = C_7_ cyclopentanone derivatives, which were studied by Bertozzi and coworkers in Jurkat cells; such *N*-substituted *O*-acetylated mannosamines have shown no detectable fluorescence following biorthogonal functionalization with a fluorescent dye [34].

## 2. Results

### 2.1. Synthesis

**Scheme 1:**
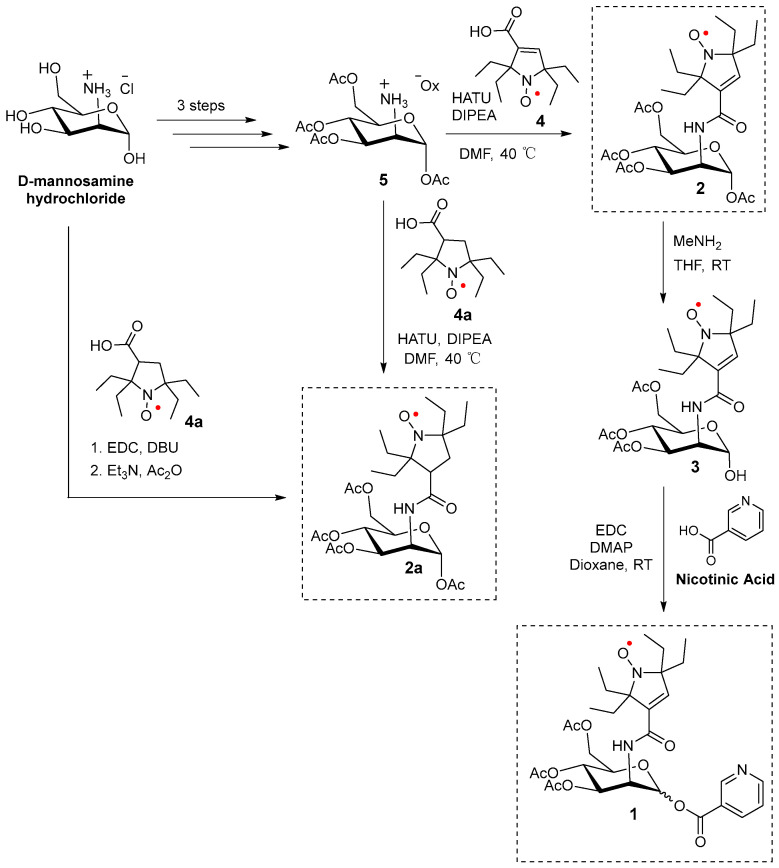
Synthesis of **1**, **2**, and **2a**.

The synthesis of **2** and **2a**, as well as nicotinate-substituted agent **1**, is initiated using commercially available D-mannosamine hydrochloride. The initial three-step synthesis of tetra-*O*-acetyl D-mannosamine oxalate **5** is performed according to the previously published protocol [43]. Compounds **2** and **2a** are synthesized from pyrroline and pyrrolidine nitroxides **4** and **4a** [15,16], respectively, using the HATU amidation reaction [44] with tetra-*O*-acetyl D-mannosamine oxalate **5**. (Alternatively, **2a** is prepared via a one-pot reaction directly from D-mannosamine hydrochloride and **4a**.) Selective hydrolysis of the *O*-acetyl group at anomeric carbon (C1) in **2** is performed using methylamine (MeNH_2_) in THF [45] to yield compound **3**. Finally, the hydroxyl (-OH) group in hemiacetal **3** reacted with nicotinic acid via an EDC-mediated esterification reaction to produce two diastereomers (**A** and **B**) of mannosamine nicotinate **1** [28] (Scheme 1).

The structures of the target compounds and key intermediates are confirmed by ^1^H-NMR, ^13^C-NMR, and IR spectroscopies, as well as high-resolution mass spectrometry (HRMS). As the NMR spectra are obtained at relatively low concentrations (typically, <100 mM), we can observe broadened peaks corresponding only to the nuclei at which the electron spin density is nearly negligible, i.e., primarily sugar moieties. Nevertheless, the resolution of NMR spectra is sufficient to allow us to determine whether the agents are isolated as single diastereomers (**1** and **2**) or as mixtures of diastereomers (**2a**). The spectral data are provided in Supporting Information (SI).

### 2.2. EPR Spectroscopy of ***2a*** in Jurkat Cells

The primary purpose of the experiments described in this section is to determine whether the nitroxide radicals may be incorporated into cells via metabolic glycoengineering by using D-mannosamine conjugates of nitroxide radicals. The rational for the in-cell EPR spectroscopy study may be summarized by the answers to these three questions:

- Will most of the nitroxide radicals remain intact after a few days of incubation at 37 °C?
- How many nitroxide radicals per cell may be incorporated into the cells?
- Will the nitroxide radical be well immobilized inside the cell?

Some 5-membered ring *gem*-diethyl nitroxide radicals, such as **4** and **4a** (Figure 1), possess superior resistance to reduction with an excess of the ascorbate/glutathione mixture, with the corresponding second-order rate constant, *k*_2_ ≈ 1 × 10^−3^ M^−1^ s^−1^ [15,16,17,18]. A similar *k*_2_ is now determined for nitroxide radical **2a** at 295 K (Supplementary Material). Nitroxide radicals **4** and **4a** also possess superior stability in liver homogenates [20]. Nevertheless, at low concentrations of derivatives of **4** in diluted cytosolic extracts, e.g., ~10 µM in the HEK293T extract, the EPR signal from the radical persisted for up to 7 h at room temperature [21] and for much shorter times in *E. coli* extracts [19]. To ensure a significant amount of nitroxide radicals after the incubation at 37 °C, we choose to employ a relatively large concentration, specifically ~0.5 mM, of nitroxide, even though this is anticipated to lead to limited cell viability (Figure S2, Supplementary Material). Conjugate **2a**, isolated as a mixture of diastereomers, provided sufficient solubility.

We select Jurkat cells for the in-cell EPR study because, based on past research [35,46], these cells are found to be among the cell lines with the highest degree of incorporation of D-mannosamine/sialic acid derivatives.

The immobilization of nitroxide radicals is measured by the rotational correlation time, *τ*_cor_. Longer *τ*_cor_ is anticipated to lead to improved relaxivities, namely *r*_1_ and *r*_2_, of the agent in MRI, as reflected by the shortening of relaxation times *T*_1_ and *T*_2_ for ^1^H of water [6]. For example, for a small molecule nitroxide with *τ*_cor_ ≈ 40 ps, *r*_1_ ≈ 0.14 mM^–1^ s^–1^ is found [6], while for macromolecular nitroxides with *τ*_cor_ ≈ 1 ns and 10 ns, *r*_1_ > 0.4 mM^–1^ s^–1^ and *r*_2_ > 4 mM^–1^ s^–1^ are determined, respectively [6,11]. Thus, we can anticipate a significant lowering of *T*_1_ and especially *T*_2_ for strongly immobilized nitroxide radicals such as those attached to glycoproteins through sialic acid (Figure 1).

**Figure 2:**
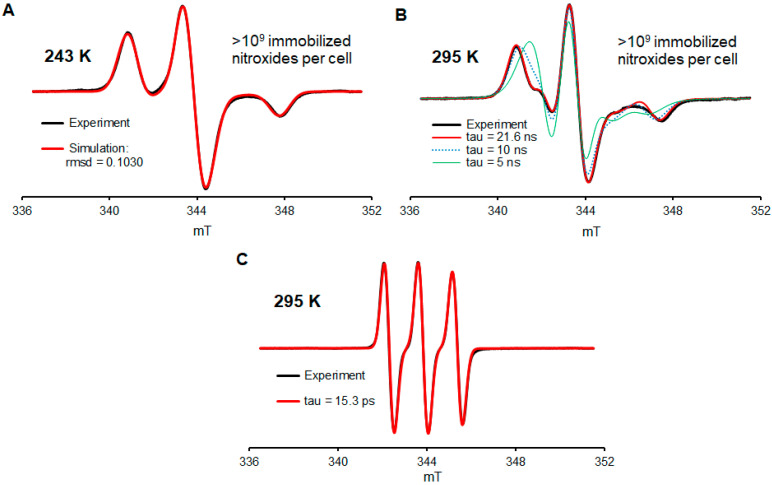
EPR spectra of conjugate **2a** in Jurkat cells. (**A**) Spectrum of washed (5×) cells at 243 K, which were used for the quantitation of nitroxide radicals. (**B**) Spectrum of washed (5×) cells resuspended in the PBS buffer at 295 K showing the predominant presence of highly immobilized nitroxide radicals. (**C**) Spectrum of the cell media with **2a** at 295 K. For more details, see Supplementary Materials (Section S2).

In the paragraphs below, we will focus on the description of experiments illustrated in Figure 2; a more detailed description, including the control and repeat experiments, is found in Supplementary Materials (Section S2). Jurkat cells are incubated in media containing 490 µM nitroxide **2a** (Figure 1) for two days (Figure S2, Supplementary Materials). Subsequently, for one part of the sample, the media are removed, and the cells are suspended in the fresh medium containing 400 µM nitroxide **2a**. Following 1 day of incubation, for one part of the sample, the media is removed, and the cells are suspended in the fresh medium without nitroxide **2a** (with a residual amount of **2a**) and incubated for 1 day. After the cell are counted (Trypan blue), the cells are subjected to five iterations of centrifugation, the removal of media, and suspension in fresh PBS. The fourth PBS wash gives the residual amount of the radical near the detection limit of the X-band EPR spectroscopy (a ~1 µM aqueous sample in a single 0.6 mm ID capillary). Following the final iteration, the cells were suspended in a minimal volume of PBS and then transferred to a 5 mm OD EPR quartz tube for quantitative EPR spectra analysis (spin counting) at 243 K; the spin and cell counts indicated >10^9^ nitroxide radicals per cell (Figure 2A and Figure S2, Supplementary Materials). However, due to long incubation times (4 d) and a high concentration of **2a**, only ca. 10% of the cells are viable. We should mention that in another part of the sample, which was incubated with 0.49 mM nitroxide for two days and then subjected to a work-up, the viable to dead cells are in the 1:1 ratio, which is similar to the EPR spectra of those in Figure 2, though with significantly fewer S/N because the spin and cell counts indicated only ≥10^8^ nitroxide radicals per cell (Figure S4, Supplementary Materials). This degree of incorporation of a label via the direct glycoengineering of cells is greater than those reported via biorthogonal reactions. We speculate that this may be due to some of the labels ending up in a sterically encumbered environment, thus inaccessible to typical biorthogonal reagents.

While at 243 K, all nitroxide radicals are immobilized in the frozen aqueous buffer medium (Figure 2A, simulation). Another spectrum, obtained in a fluid medium at 295 K from the same sample but diluted with extra volume of PBS, indicated that 90+% of nitroxide radicals are well immobilized, with a rotational correlation time of *τ*_cor_ ≥ 20 ns (Figure 2B, Table S1, Supplementary Materials). For comparison, the EPR spectra of nitroxide **2a** in the cell medium at 295 K indicate *τ*_cor_ in the picosecond range (Figure 2C), as expected for a small molecule nitroxide radical [6,47]. We note that the degree of immobilization of nitroxide radical **2a** is comparable to that obtained by biorthogonal labeling of the MBP (211/295 mutant) protein with a derivative of **4** in live HEK293T cells [21]. However, this *τ*_cor_ ≥ 20 ns is much longer than *τ*_cor_ = 0.115 ns for nitroxides strongly complexed inside cucurbit [7], uril [47], or spirocyclohexyl pyrrolidine nitroxides immobilized by conjugation to polymers used for MRI such as *τ*_cor_ = 1.5 ns in dendrimer ORCA [6] or *τ*_cor_ ≈ 10 ns in the brush-arm star polymer BASP-ORCA [11].

### 2.3. In Vivo MRI of ***1*** and ***2***

With promising results obtained using the in-cell EPR spectroscopy, i.e., a high degree of cellular incorporation of highly immobilized nitroxide radicals, we proceed to mouse MRI experiments. All mice are injected in the tail vein with a total of 18 mg of the mORCAs in three equal doses separated by 24 h, i.e., at 0, 24, and 48 h [28]; this dosing corresponds to 3 × 9.5 and 3 × 10.5 µmol per mice for mORCAs **1** and **2**, respectively. (For two mice, the third injection of **2** had to be i.p. because of a broken vein.) A total of two and nine mice are imaged after the injection of mORCAs **1** and **2**, respectively (Table S2, Supplementary Materials). For most mice, MRI *T*_1_ and *T*_2_ maps are acquired at 0 h (pre-injection), 0.5 h (post-injection), 24 h (pre-injection), 24.5 h (post-injection), 48 h (pre-injection), 48.5 h (post-injection), and 72 h (final scan). Each organ is mapped using 10 slices. The experiments are designed to optimize the MRI images, as measured by decreases in ^1^H relaxation times *T*_1_ and *T*_2_, which are obtained in the final scan at 72 h after the initial injection of the mORCA.

**Figure 3:**
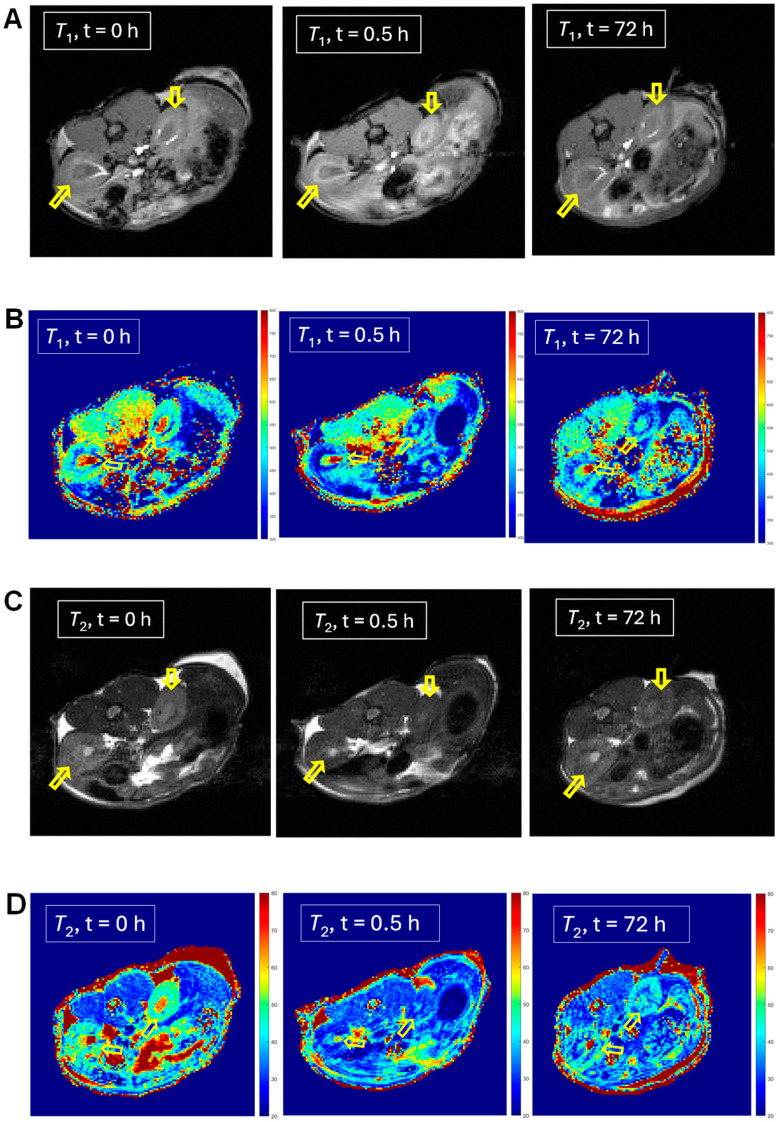

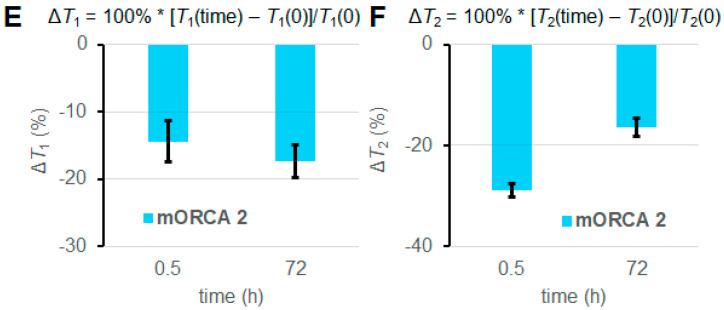
MRI *T*_1_-weighted images (**A**), *T*_1_ maps (**B**), *T*_2_-weighted images (**C**), and *T*_2_ maps (**D**) of kidneys in a selected mouse injected with mORCA **2** at 0 h (pre-injection), 0.5 h (post-injection), and 72 h (final image). Small, yellow arrows point to kidneys, and for maps, color-bar ranges are 300–800 ms (*T*_1_) and 20–80 ms (*T*_2_), from blue to red. A summary of *T*_1_ (**E**) and *T*_2_ (**F**) relative vs. pre-injection at the 0 h decrease for post-injection at 0.5 h and the final image at 72 h. For more details, see Table 1 and Supplementary Materials (Section S3).

For selected mice (*n* = 1), we can observe significantly lowered ^1^H relaxation times *T*_1_ and *T*_2_ following the injection of mORCA **2**, qualitatively judged by the color changes from yellow/green to green/blue in the *T*_1_ and *T*_2_ maps after 24 h following the third injection of the mORCA (final scan at 72 h), as illustrated for kidneys in Figure 3B,D. Corresponding changes are found in the *T*_1_- and *T*_2_-weighted images with the brightening and darkening of kidneys, respectively (Figure 3A,C). Quantitative analyses of *T*_1_ and *T*_2_ maps in all slices showing the organ, using regions of interests (ROIs), indicate statistically significant decreases of Δ*T*_1_ = –17 ± 2% and Δ*T*_2_ = –16 ± 2% at 72 h (Figure 3E,F).

For selected mice (*n* = 1) injected with mORCA **2**, all other organs, such as the liver, heart, and brain, show statistically significant decreases of Δ*T*_1_ and Δ*T*_2_ (Table 1, Supplementary Materials, Section S3). For selected mice (*n* = 1) injected with mORCA **1**, statistically significant decreases in Δ*T*_1_ and Δ*T*_2_ are obtained for kidneys but not for the liver or brain (Table 1, Supplementary Materials, Section S3). While *n* = 1 refers to a single, selected mouse, statistical significance is derived from the large number of independent regions of interests (ROIs) analyzed per organ (60–196 ROIs, with each ROI containing ~9 voxels). Each ROI provides an independent measurement of *T*_1_ and *T*_2_, allowing for meaningful statistical analysis within a single mouse.

**Figure 4:**
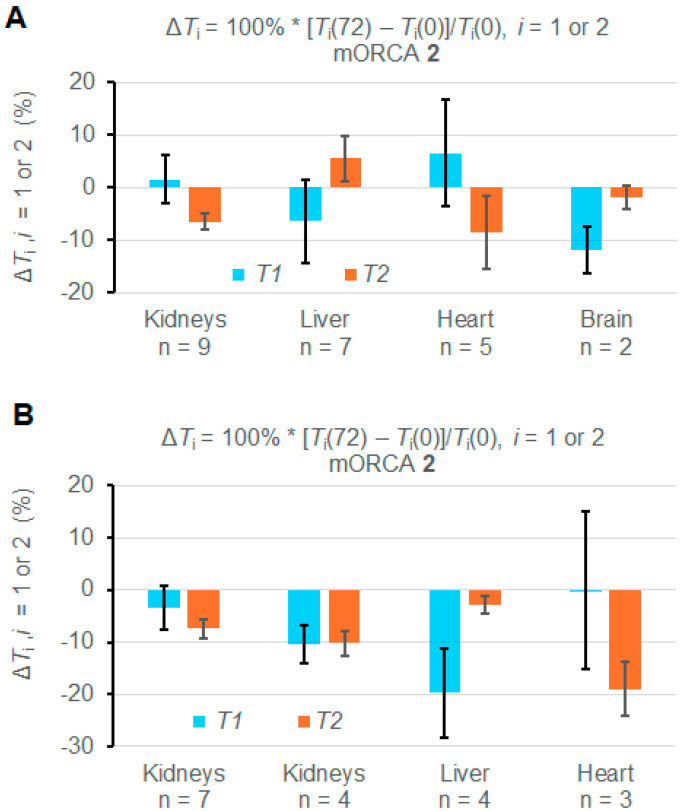
Summary of relative Δ*T*_1_ and Δ*T*_2_ (%) for *T*_1_ and *T*_2_ maps at 72 h vs. 0 h (pre-injection) for mice injected with mORCA **2**. (**A**) Maps are acquired for all studied animals. (**B**) Maps are for selected animals (*n* = 3–7). For more details, see Table 1 and Supplementary Materials (Section S3).

However, some mice show abnormal increases of *T*_1_ and *T*_2_ upon the injection of the mORCA (Supplementary Materials, Section S3). Indeed, statistical analyses of ROIs from each organ in the 11 mice indicate that most changes in *T*_1_ and *T*_2_ over the course of 72 h are significant; however, some of them are positive (Figures S25–S37, Supplementary Materials). Consequently, when values of Δ*T*_1_ and Δ*T*_2_ are averaged over all mice injected with mORCA **2**, i.e., *n* = 9, 7, 5, and 2 for the kidneys, liver, heart, and brain, respectively or over two mice injected with mORCA **1**, the changes in most organs are either insignificant or even positive (Table 1, Figure S19, Supplementary Materials). Only the kidneys (*n* = 9) and brain (*n* = 2) show significant decreases in *T*_2_, with Δ*T*_2_ = –6.5 ± 1.6%, and *T*_1_, with Δ*T*_1_ = –11.9 ± 4.5%, respectively (Figure 4A).

Nevertheless, it is possible to select mice, such as *n* = 7 for kidneys, *n* = 4 for the liver, and *n* = 3 for the heart, to provide negative values for both Δ*T*_1_ and Δ*T*_2_ and with significant Δ*T*_2_ = –7.4 ± 1.9% for kidneys and Δ*T*_2_ = –19 ± 5% for the heart (Figure 4B). For *n* = 4 kidneys, both Δ*T*_1_ and Δ*T*_2_, and for *n* = 3 livers, Δ*T*_1_ corresponds to significant relaxation time decreases (Table 1 and Figure 4B).

### 2.4. Ex Vivo EPR Spectroscopy of ***1*** and ***2***

The identical mice that are imaged by MRI are sacrificed immediately following the last imaging, which is 72 h after the initial injection and 24 h after the last, that is, the third injection of mORCAs **2** (four mice) and **1** (two mice). Their organ homogenates and blood are analyzed via ex vivo EPR spectroscopy at 243 and 294 K.

**Figure 5:**
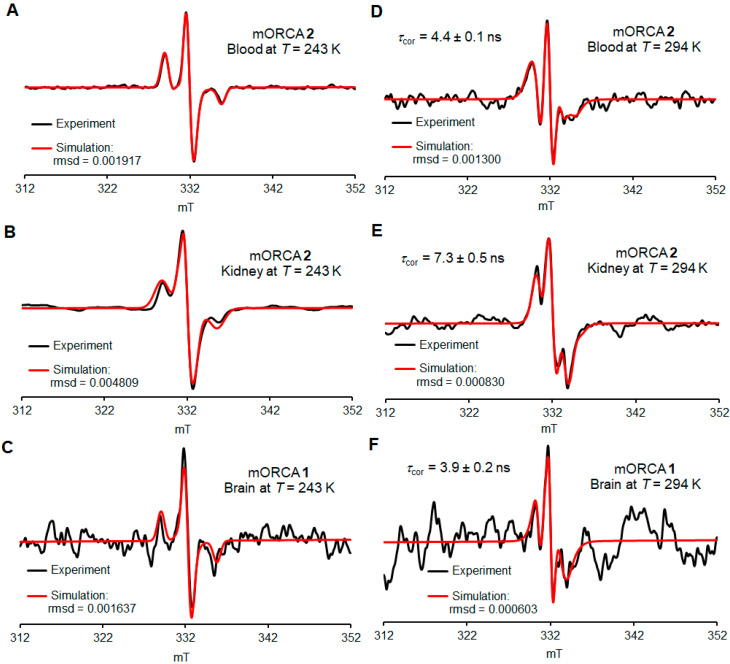
Selected ex vivo EPR spectra for the blood, kidney, and brain at 243 K (**A**–**C**) and 294 K (**D**–**F**). Simulations of spectroscopic quantification are carried out 72 h after the initial injection of contrast agents **1** (orange bars) or **2** (blue bars). For more details, see Supplementary Materials (Section S4), including Tables S3–S5.

**Figure 6:**
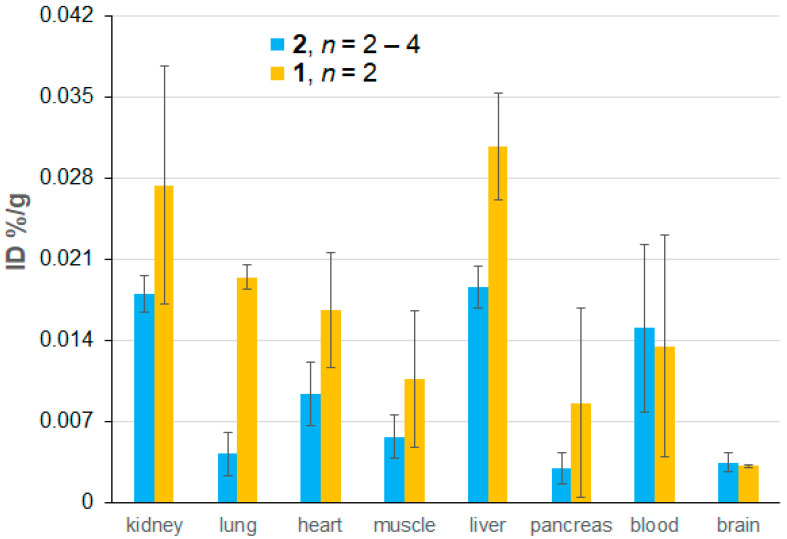
Biodistribution of nitroxide radicals as a percentage of the injected dose (%ID) based on ex vivo EPR spectroscopic quantification, which is carried out 72 h after the initial injection and 24 h after the last injection of mORCAs **1** (orange bars) or **2** (blue bars). Each animal received three 6 mg doses of mORCAs spaced by 24 h. The reported values represent the mean ± SEM, with *n* = 2 for **1** and *n* = 4 for **2**, except for the muscle and brain for **2** where *n* = 3 and *n* = 2, respectively. For more details, see Supplementary Materials (Section S4), including Table S3.

The spectra at 243 K, in a frozen PBS buffer, reveal the presence of nitroxide radicals that are rigidly immobilized (Figure 5A–C, Figures S41–S45, and Table S4, Supplementary Materials). These spectra are used for quantitative spin counting to determine the spin concentration of nitroxide radicals in each tissue (nmol g^−1^) and, therefore, the percent of the injected dose (%ID) (Figure 6).

The spectra at 294 K, in a fluid PBS buffer, reveal the presence of reasonably well-immobilized nitroxide radicals with *τ*_cor_ ≥ 4 ns as the major component in the blood, kidneys, livers, lungs, and brain (Figure 5D–F and Table 2, Figures S46–S70, and Table S5, Supplementary Material). Notably, the EPR spectra of blood can be simulated with just a single component of well-immobilized nitroxide radicals with *τ*_cor_ = 4.0 ± 0.3 ns. We hypothesize that the putative fast nitroxide radical in blood is complexed and thus immobilized by binding to mannose-binding lectin (MBL). MLB is present in mouse serum at a relatively high concentration of 50 µg mL^−1^ (MLB-A and MLB-C) and shows considerable affinity to mannoses including *N*-acetyl mannosamine [48]. This concentration corresponds to about 0.7 nmol mL^−1^ for monomeric MLB [49], which is comparable to 1.3–1.6 nmol g^−1^ concentrations in blood for mORCAs **1** and **2** (Figure S39, Supplementary Material).

Only freshly thawed homogenates give the spectra with acceptable S/N ratios, with the following spectra showing a significant decay (Figure S40, Supplementary Materials). For some organs, such as the pancreas, heart, and muscle, spectra at 294 K do not show peaks corresponding to the nitroxide radical, and for the brain, only one spectrum (out of a couple dozen) could be adequately simulated (Figure 5F and Figure S43B, Supplementary Material).

With the constraint of small *n* = 2 for mORCA **1** and large error bars, differences in radical concentration between **1** and **2** are statistically insignificant for each organ, except for the lung (*p* = 0.00036), where a much higher nitroxide radical concentration is found for **1** (Figure 6 and Figure S39, Supplementary Materials). In addition, a small, similar amount of nitroxide radicals penetrates the blood–brain barrier (BBB) in healthy mice for both mORCAs **1** and **2** (Figure 6), a contrast to the previous findings of a significant improvement in BBB permeability by nicotinate moiety [28]. Similar (and limited) permeability of both **1** and **2** may be associated with the presence of DMSO [50,51,52], or perhaps with the hydrophobic nitroxide moiety mimicking one of the substrates for the transporters within BBB.

The presence of immobilized nitroxide radicals with *τ*_cor_ ≥ 4 ns would suggest very good relaxivity that is comparable to BASP-ORCA. However, the biodistribution of nitroxide radicals indicates that %IDs for mORCAs are a factor of ≥10 smaller for most tissues (and a factor of >100 for blood), compared to those in ORCAFluor 24 h after the contrast injection (Table 3), and about a factor of ~100 vs. BASP-ORCA 21 h after the contrast injection [10,11,12]. These relatively low spin concentrations of nitroxide radicals in all tissues, <3 nmol/g (Figure S39, Supplementary Material), would explain the rather small decreases in *T*_1_ and *T*_2_ observed in the corresponding MRI maps.

## 3. Materials and Methods

### 3.1. Materials

Unless otherwise noted, all solvents used were commercially available and used directly from suppliers without further purification. Perdeuterated solvents for NMR spectroscopy were obtained from Cambridge Isotope Laboratories. Unless otherwise indicated, commercially available chemicals were obtained from either Oakwood or Ambeed. Standard techniques under an inert atmosphere, using vacuum lines and Schlenk vessels, were employed for synthesis.

Column chromatography (0–20 psig pressure) was performed using Sorbtech silica gels (60 Å, 40–75 µm). Analytical TLC was performed on 0.25 mm Millipore Sigma silica plates (60F-254) using UV light as the visualizing agent.

### 3.2. Instruments

^1^H-NMR and ^13^C-NMR spectra were obtained using Bruker spectrometers (400 and 700 MHz), with CDCl_3_, *D*_2_O, acetone-*d*_6_, or DMSO-*d*_6_ as the solvent. The chemical shift references in deuterated solvents for ^1^H NMR were as follows: CDCl_3_, 7.26; *D*_2_O, 4.79; and acetone-*d*_6_, 2.05. The 700 MHz instrument was equipped with a cryoprobe. IR spectra were obtained using a commercial instrument equipped with an ATR sampling accessory. High-resolution mass spectra (HRMS) were obtained at local facilities for mass spectrometry.

### 3.3. Synthesis

Compound **5** is synthesized from D-mannosamine hydrochloride according to a previously published protocol, as outlined in Supplementary Materials [37]. Spectral data as well as extra experimental detail, including the preparations of **2a**, are provided in Supplementary Material.

**1,3,4,6-Tetra-*O*-acetyl-mannose *N*-nitroxide 2:** Compound **5** (105.0 mg, 0.240 mmol) was loaded in a Schlenk tube and evacuated under high vacuum overnight. HATU (91.3 mg, 0.240 mmol) was loaded in another Schlenk tube. Pyrroline nitroxide **4** (48.4 mg, 0.204 mmol), which was synthesized according to the published literature [15], was added, and the tube was evacuated under high vacuum overnight. Under an argon atmosphere, anhydrous DMF (1.0 mL) and DIPEA (100 µL, 0.574 mmol) were added, and the mixture was stirred at room temperature. After stirring for 1 h, the above reaction mixture was added to the Schlenk tube containing compound **5** and stirred under argon at room temperature for 60 h. The crude product was transferred to a vial and concentrated under a N_2_ flow. Crude product was purified via column chromatography on a silica gel using pentane/ethyl acetate (1/1, *R*_f_ = 0.57) to yield compound **2** as a pale-yellow solid foam (94.2 mg, 83%).

**1-Hydroxy-3,4,6-tri-*O*-acetyl-mannose *N*-nitroxide 3:** Compound **2** (60.9 mg, 0.107 mmol) was added to a Schlenk tube and evacuated under a high vacuum line for 1 h. Methylamine (4.3 mg, 0.139 mmol) in THF (distilled with sodium/benzophenone, 0.54 mL) was added under an argon atmosphere and stirred at room temperature for 17 h. THF was removed through evaporation under a N_2_ flow to obtain the crude product as yellow oil. The crude product was then purified using column chromatography on a silica gel using pentane/ethyl acetate (1/1, *R*_f_ = 0.27) to obtain compound **3** as yellow oil (37.4 mg, 66% yield).

**1-*O*-Nicotinate-3,4,6-tri-*O*-acetyl-mannose *N*-nitroxide 1:** Compound **3** (35.5 mg, 0.067 mmol) was added to a Schlenk tube and evacuated under a high vacuum line for 1 h. Nicotinic acid (17.5 mg, 0.142 mmol), DMAP (4.22 mg, 0.035 mmol), and EDC (38.7 mg, 0.202 mmol) were added to the Schlenk tube. Under argon, dioxane (0.6 mL) was added. The heterogenous suspension turned into a yellow homogeneous solution after stirring for 1 h. The reaction mixture was stirred at room temperature for 17 h. The solvent was then evaporated under a N_2_ flow to obtain the crude product as yellow oil. The purification of the crude product was performed with a preparative TLC plate using regular silica and pentane/ethyl acetate (1/1, *R*_f_ = 0.15 and 0.19 for spots A and B, respectively). Diastereomers of compound **1** were isolated and dried under a high vacuum line overnight. Diastereomeric spots **A** (14.9 mg) and **B** (20.2 mg) of compound **1** were obtained as yellow oil (35.1 mg, 82% yield).

### 3.4. In-Cell EPR Spectroscopy

CW X-band EPR spectra are acquired on a Bruker EMX instrument equipped with a frequency counter and nitrogen flow temperature control (110–300 K). All spectra are obtained in a dual mode cavity, with an oscillating magnetic field perpendicular (TE102) to the swept magnetic field (parallel mode, TE012, is not used).

Jurkat cells are incubated in media containing 490 µM nitroxide **2a** for two days. One of the three parts of the sample (Figure S2, Supplementary Materials) is centrifuged; media are removed and suspended in media containing 400 µM nitroxide and incubated for one day. Subsequently, the cells are centrifuged; the media are removed and suspended in media containing no nitroxide and then incubated for one day, followed by analysis. The work-up for analysis involves counting the cells (with staining using Trypan blue to count alive vs. dead cells), followed by five iterations of centrifugation, the removal of the media, and suspension in PBS. Four washes are found to be enough to reduce the radical concentration of the supernatant PBS to within the measurement error of fresh PBS, indicating no further radicals could be removed by washing. Finally, the cells are suspended in a minimal volume of PBS and transferred to a 5 mm OD quartz EPR tube.

Spin counting is accomplished by comparing the radical concentration in cell pellets to a solid sample of TEMPOL of known mass. Solid TEMPOL is loaded into a 5 mm long tube made from a coffee straw sealed with parafilm, thus preventing electrostatic forces from scattering the solids in TEMPOL. The straw vessel is lowered into a 5 mm OD EPR quartz tube and positioned such that all the TEMPOL is within the EPR cavity and would be accounted for in the measurement. Jurkat cells are suspended in a minimal volume of PBS and then transferred to a 5 mm OD EPR quartz tube. The resultant mixture has smaller dimensions than those of the microwave cavity, meaning that the entire sample is analyzed, and any small differences in PBS volume are inconsequential. Spectra are acquired at 243 K (Q values: 1600 for cells, 3800–4100 for TEMPOL). According to the spin counting results (Figure S2, Supplementary Materials), the greatest concentration of nitroxides per cell is observed after 3–4 days of incubation. For the cells which were incubated with nitroxide most of the time, this is not surprising. However, incubation in nitroxide-free media for one day after the initial incubation period also increases the cellular nitroxide concentration, most likely because of a significant amount of **2a** left over after a single centrifugation during the media exchange.

In addition, for the spectra at 295 K, a portion of the final cell pellet is loaded with extra PBS buffer into four stoppered 0.6 mm ID EPR quartz capillaries, which are then placed into a 5 mm OD EPR quartz tube (Figure S5B).

### 3.5. In Vivo MRI

Eight-week-old male C57BL/6J mice (Jackson Laboratory, Bar Harbor, ME, USA) were used for all experimental procedures. Mice received access to food and water ad libitum and were stored at a controlled temperature (21.5 ± 1.5 °C) in a 14 h light/10 h dark cycle. Experimental procedures were performed in accordance with the Animal Care and Use Committee at the University of Nebraska—Lincoln (UNL IACUC approval number 2300).

Mice were imaged on a 9.4 T (400 MHz for protons) MRI system (Varian) with a 40.0 mm Millipede RF imaging probe and triple-axis gradients (100 G/cm max, 1000 mT/m). The software used for imaging was VnmrJ 3.0c (Agilent, Santa Clara, CA, USA). During imaging, mice were anesthetized with 1.5–2.0% isoflurane via inhalation. Then, anesthesia with isoflurane was reduced to 0.5–1.0% isoflurane. Breathing was monitored and maintained between 50 and 80 breaths per minute (Small Animal Instruments, Inc., Stony Brook, NY, USA).

The intravenous (*i.v.*) administration of mORCAs **1** and **2** in mice was conducted for the in vivo MRI study. The mice were anesthetized via inhalation of isoflurane and subjected to a baseline MRI scan on day 1 (0 h), before any injection. The mice were intravenously injected with 6 mg of the contrast agent (9.48 µmol of compound **1** or 10.5 µmol of compound **2**) in 120 µL of the DMSO/H_2_O (9:1) mixture (i.e., 50 mg mL^−1^) through the tail vein. While all eleven mice (two with **1** and nine with **2**) tolerated the agents injected at a concentration of 50 mg mL^−1^ well, the injection of **2** at a much higher concentration of 120 mg mL^−1^ into one mouse resulted in an instant death of the animal, most likely due to the precipitation of the agent in the bloodstream. Post-injection scans are subsequently performed. For days 1 to 4, MRI scans are acquired before and after the injections of the contrast agent to obtain *T*_1_-weighted, *T*_2_-weighted, *T*_1_ map, and *T*_2_ map images for various organs, including the kidneys, liver, heart, and brain. Each organ was scanned transversely in ten slices along the *z*-axis. For all eleven mice, 3 injections of the agent (at 0, 24, and 48 h), with a total dose of 18 mg per animal, were performed. For two animals, the third injection of **2** had to be *i.p.* due to a broken tail vein.

For in vivo MRI sessions, *T*_1_-weighted images were acquired with a gradient echo multi-slice sequence (GEMS), repetition time (TR) = 51.64 ms, echo time (TE) = 2.60 ms, flip angle (FA) = 60°, averages = 5, matrix size = 128 × 128, field of view (FOV) = 30 mm × 30 mm, slice thickness = 1 mm, number of slices = 10, and effective in-plane resolution = 234 µm x 234 µm (scan time = 33 s). To collect the *T*_1_ measurements, a variable FA method was applied. The FAs obtained were at 10°, 30°, and 50°, with all the same parameters as the *T*_1_-weighted GEMS.

*T*_2_-weighted images were acquired with a fast spin-echo multi-slice sequence (FSEMS). Repetition time (TR) = 2000 ms, echo spacing (ESP) = 10 ms, echo train length/ number of segments (ETL/ Seg) = 16/8, k-zero = 4, effective echo time (effTE) = 40.0 ms, averages = 1, matrix size, FOV, and the effective in-plane resolution were the same as T1-weighted images (scan time= 36 s).

*T*_2_ measurements were collected using a multi-echo multi-slice sequence (MEMS), and TR = 3000 ms, TE = 10 ms, number of echoes = 10, averages = 1, matrix size, FOV, and the effective in-plane resolution were the same as *T*_1_-weighted images (scan time = 6 min, 24 s).

All acquired images were then processed in MATLAB R2021 (MathWorks, Natick, MA, USA) using custom-built scripts. In particular, absolute *T*_1_ and *T*_2_ values were extracted directly from MRI *T*_1_ and *T*_2_ map files using a custom-built MATLAB script. To evaluate changes in *T*_1_ and *T*_2_ relaxation times across different time points, statistical significance was assessed using ordinary one-way ANOVA, comparing means for the same organ at each time point. Significance levels were denoted as follows: * *p* < 0.05, ** *p* < 0.01, *** *p* < 0.001, and **** *p* < 0.0001. All statistical analyses and graphical representations were performed using GraphPad Prism 10.

### 3.6. Ex Vivo EPR Spectroscopy

Ex vivo CW X-band EPR spectra were obtained as described for in-cell spectra, except an upgraded Bruker EMXplus instrument, with high-sensitivity Bruker cavity (perpendicular mode), was used.

After the final MRI scan on day 4, i.e., 72 h after the initial, baseline MRI scan followed by agent injection and, for most mice, 24 h after the last, that is, the third injection, the mice were anesthetized at 3% isoflurane and cardio-perfused with a PBS buffer. Cardiac perfusion was performed to ensure that the EPR signal of nitroxide in the blood does not interfere with the signal from nitroxide incorporated into the cells of the organs. After cardiac perfusion, the organs were collected and immediately frozen in a dry ice box to preserve their integrity.

Prior to EPR spectroscopy, each organ is weighed in a vial to determine its mass; then, a known volume of the 0.5 mM PBS buffer, typically 400 μL, is added to the vial. The sample is homogenized at 0 °C with a rotor stator homogenizer and then transferred to an EPR quartz capillary or 2 mm OD EPR quartz tube for qualitative measurement at 295 K and to a 4 mm OD EPR quartz tube for quantitative measurement at 243 K. For some mice, the samples in 2 mm OD tubes are used first for quantitative measurement at 243 K and then for qualitative study at 295 K. To improve the spectral baselines, cavity backgrounds are subtracted from each organ spectrum by using the spectra of the PBS buffer, obtained with identical parameters. When needed, an additional baseline correction (quadratic or linear) is carried out prior to the spectral simulations (and/or within the spectral simulation). The spin concentrations of nitroxide radicals in organs (nmol g^−1^, nmol of *S* = ½ nitroxide radical per gram of tissue) are measured at 243 K to increase the signal-to-noise ratio for the aqueous samples. A fresh solution of known concentration of 3-carboxy-PROXYL nitroxide radical is also prepared in the same PBS buffer and same diameter tube to provide a reference for spin counting at 243 K. The samples of all solutions/homogenates possess a height of at least 5 cm to ensure complete cavity filling. The samples are degassed via sonication, as needed (for example, when gas bubbles are visible). Typical parameters are as follows: microwave power attenuation (20 dB), modulation amplitude (0.5 mT), spectral width (20-40 mT), resolution (800 points), and sweep time (20 s); these parameters are kept identical for the organs, references, and cavity backgrounds. The number of scans (8–64) and receiver gain are adjusted as needed for each sample. Occasionally, a spectrum of 2,2-diphenyl-1-picrylhydrazyl (DPPH) powder is obtained to serve as a *g*-value reference (*g* = 2.0037) to confirm that all detected nitroxide radicals have *g* = 2.005–2.006.

## 4. Conclusions

We have successfully synthesized and characterized two conjugates of nitroxide radical with D-mannosamine as potential metabolic organic radical contrast agents (mORCAs) for MRI. Notably, our approach circumvents the need for biorthogonal reactions, following metabolic glycan engineering. We demonstrate an efficient incorporation of highly immobilized nitroxides in cells via in-cell EPR spectroscopic experiments. In vivo MRI of selected mice shows that mORCA **2** could shorten the *T*_1_ and *T*_2_ relaxation times of protons in water located in the kidney, liver, heart, and brain by up to 40% after 3 days. Nevertheless, the analyses of all studied mice show that only kidneys (*n* = 9) with Δ*T*_2_ = –6.5 ± 1.6% and the brain (*n* = 2) with Δ*T*_1_ = –11.9 ± 4.5% possess significantly shortened *T*_2_ and *T*_1_, respectively. Because of a low signal-to-noise ratio in the MRI data, more accurate quantitative analyses are needed to further investigate the effect of mORCA on the relaxation times; in particular, gating imaging to the cardiac and breath cycles may reduce motion artifacts and increase the signal-to-noise ratio of the Δ*T*_1_ and Δ*T*_2_ values.

The ex vivo EPR spectroscopic analyses indicate that the biodistribution of the nitroxide radicals in mouse tissues is primarily localized in the kidneys, liver, lung, and blood, which contain primarily highly immobilized nitroxide radicals, with *τ*_cor_ ≥ 4 ns. Notably, blood contains exclusively immobilized nitroxide radicals with *τ*_cor_ = 4.0 ± 0.3 ns. The spin concentrations of nitroxide radicals in tissues, which were measured 24 h after the last (third) mORCA injection, are ≤3 nmol g^−1^, which is approximately one-to-two orders of magnitude smaller than for bis-spirocyclohexyl nitroxide-based bottlebrush polymers and BASP-ORCA (21–24 h after a single contrast injection), thus providing the rationale for the observed small relaxation time changes in in vivo MRI.

Our study provides valuable insights into the potential use of nitroxide mannosamine mORCA in biomedical imaging, and further research is warranted in this area.

## Figures and Tables

**Table 1:** Summary of changes in ^1^H relaxation times in different organs 24 h after the third (last) injection of mORCAs **1** and **2**.

Organ		2			1	
		**Δ*T*_1_ (%)**	**Δ*T*_2_ (%)**		**Δ*T*_1_ (%)**	**Δ*T*_2_ (%)**
Kidneys	*n* = 1	**−17 ± 2**	**−16 ± 2**	*n* = 1	**−21 ± 2**	**−17 ± 1**
Kidneys	*n* = 9	+2 ± 5	**−6.5 ± 1.6**	*n* = 2	−8 ± 13	−7 ± 10
Kidneys	*n* = 7	−3.4 ± 4.2	**−7.4 ± 1.9**			
Kidneys	*n* = 4	**−10.5 ± 3.7**	**−10.2 ± 2.4**			
Liver	*n* = 1	**−41 ± 3**	**−7.5 ± 2.6**	*n* = 1	−1 ± 4	0 ± 3
Liver	*n* = 7	−6 ± 8	+6 ± 4	*n* = 2	+9 ± 10	+7 ± 7
Liver	*n* = 4	−19.7 ± 8.5	−2.9 ± 1.7			
Liver	*n* = 3	**−26.0 ± 8.1**	−3.8 ± 2.1			
Heart	*n* = 1	**−26 ± 7**	**−28 ± 5**	*n* = 1		
Heart	*n* = 5	+7 ± 10	−8 ± 7	*n* = 2		
Heart	*n* = 3	0 ± 15	**−19 ± 5**			
Brain	*n* = 1	**−16 ± 1**	**−4.0 ± 1.1**	*n* = 1	+8 ± 1	−1.3 ± 0.8
Brain	*n* = 2	**−11.9 ± 4.5**	−1.7 ± 2.2	*n* = 2	+10 ± 2	−0.5 ± 0.9

Values in bold correspond to statistically significant decreases in relaxation times.

**Table 2:** Summary of simulations of ex vivo EPR spectra at 294 K.

Tissue	*n* ^1^	*τ*_cor_ (ns) ^2^	Fast Component ^2,3^
Blood	7	4.0 ± 0.3	0.00
Kidney	10	6.0 ± 0.5	0.40 ± 0.06
Liver	4	9.2 ± 0.4	0.37 ± 0.05
Lung	4	5.5 ± 0.6	0.51 ± 0.03
Brain	1	3.9	0.73

^1^ *n* = number of EPR spectra simulated. ^2^ mean ± SEM. ^3^ Content of the fast component with *τ*_cor_ fixed to 5 ps, with the slow component with *τ*_cor_ ≥ 4 ns normalized to 1.00.

**Table 3:** Comparison of biodistributions of nitroxide radicals as %ID based on ex vivo EPR spectroscopic quantification 24 h after the last injection of mORCA **1**, mORCA **2**, and the spirocyclohexyl nitroxide-based bottlebrush polymer (ORCAFluor).

Tissue	mORCA 1		mORCA 2		ORCAFluor
	%ID (*n*)	% ^1^	%ID (*n*)	% ^1^	%ID
Blood	0.014 ± 0.010 (2)	0.24	0.015 ± 0.007 (4)	0.27	5.6 ± 0.7
Kidney	0.027 ± 0.010 (2)	5.7	0.018 ± 0.002 (4)	3.8	0.48 ± 0.06
Liver	0.031 ± 0.005 (2)	10.8	0.019 ± 0.002 (4)	6.6	0.28 ± 0.08
Lung	0.020 ± 0.001 (2)	1.7	0.004 ± 0.002 (4)	0.36	1.2 ± 0.2
Heart	0.017 ± 0.005 (2)	2.5	0.009 ± 0.003 (4)	1.4	0.67 ± 0.10
Pancreas	0.009 ± 0.008 (2)	na	0.003 ± 0.001 (4)	na	na
Muscle	0.011 ± 0.006 (2)	na	0.006 ± 0.002 (3)	na	na
Spleen	0.019 ± 0.004 (2)	2.6	0.023 ± 0.007 (2)	3.2	0.72 ± 0.14
Brain	0.0032 ± 0.0001 (2)	na	0.0035 ± 0.0008 (2)	na	<<0.001

^1^ Relative mORCA %ID as percentage of the %ID for ORCAFluor. Single doses of mORCAs **1**, **2**, and ORCAFluor are 9.5, 10.5, and 7.9 µmol of nitroxide radicals, respectively.

## Data Availability

The raw data supporting the conclusions of this article will be made available by the authors upon request.

## Supplementary Materials

The following supporting information can be downloaded at https://www.mdpi.com/article/10.3390/molecules30071581/s1, detailed description of synthesis of mORCAs and their characterization, including in-cell and ex vivo EPR spectroscopy, and MRI in mice. Tables S1–S5; Figures S1–S98. References [15,28,43,44,45] is cited in Supplementary Material.
