# Supplementary material for: Towards Metabolic Organic Radical Contrast Agents (mORCAs) for Magnetic Resonance Imaging
Source: Molecules. 2025 Apr 2;30(7):1581. doi: 10.3390/molecules30071581 (PMC11990138; doi:10.3390/molecules30071581)
Supplement: Supplementary file 1 [file molecules-30-01581-s001.zip › molecules-3530071-supplementary.pdf]

Supporting Information

Shuyang Zhang [1,†], Sabina Dhakal [1], Evan Curtis [2], Hunter Miller [2], Joseph T. Paletta [1], Connor Gee [2], Suchada Rajca [1], Forrest Kievit [2] and Andrzej Rajca [1,*]

[1] Department of Chemistry, University of Nebraska, Lincoln, NE 68588-0304, USA; zhangshuyangyili@gmail.com (S.Z.); sdhakal6@huskers.unl.edu (S.D.); joepaletta21@gmail.com (J.T.P.); srajca.chem@gmail.com (S.R.)
[2] Department of Biological Systems Engineering, University of Nebraska, Lincoln, NE 68583-0900, USA; ecurtis@huskers.unl.edu (E.C.); hmiller23@unl.edu (H.M.); connor.gee@huskers.unl.edu (C.G.); fkievit2@unl.edu (F.K.)
* Correspondence: arajca1@unl.edu
† Current address: School of Engineering and Applied Sciences, Harvard University, Allston, MA 02134, USA.

Table of contents

1. Synthesis of **1**, **2**, and **2a**.
2. Summary of *in-cell* EPR spectroscopy.
3. Summary of in vivo MRI.
4. Summary of ex vivo EPR spectroscopy.
5. Characterization of **1**, **2**, **2a**, and selected intermediates via ^{1}H and ^{13}C NMR, IR, EPR spectroscopies, and mass spectrometry (MS).

1. Synthesis of 1, 2, and 2a.

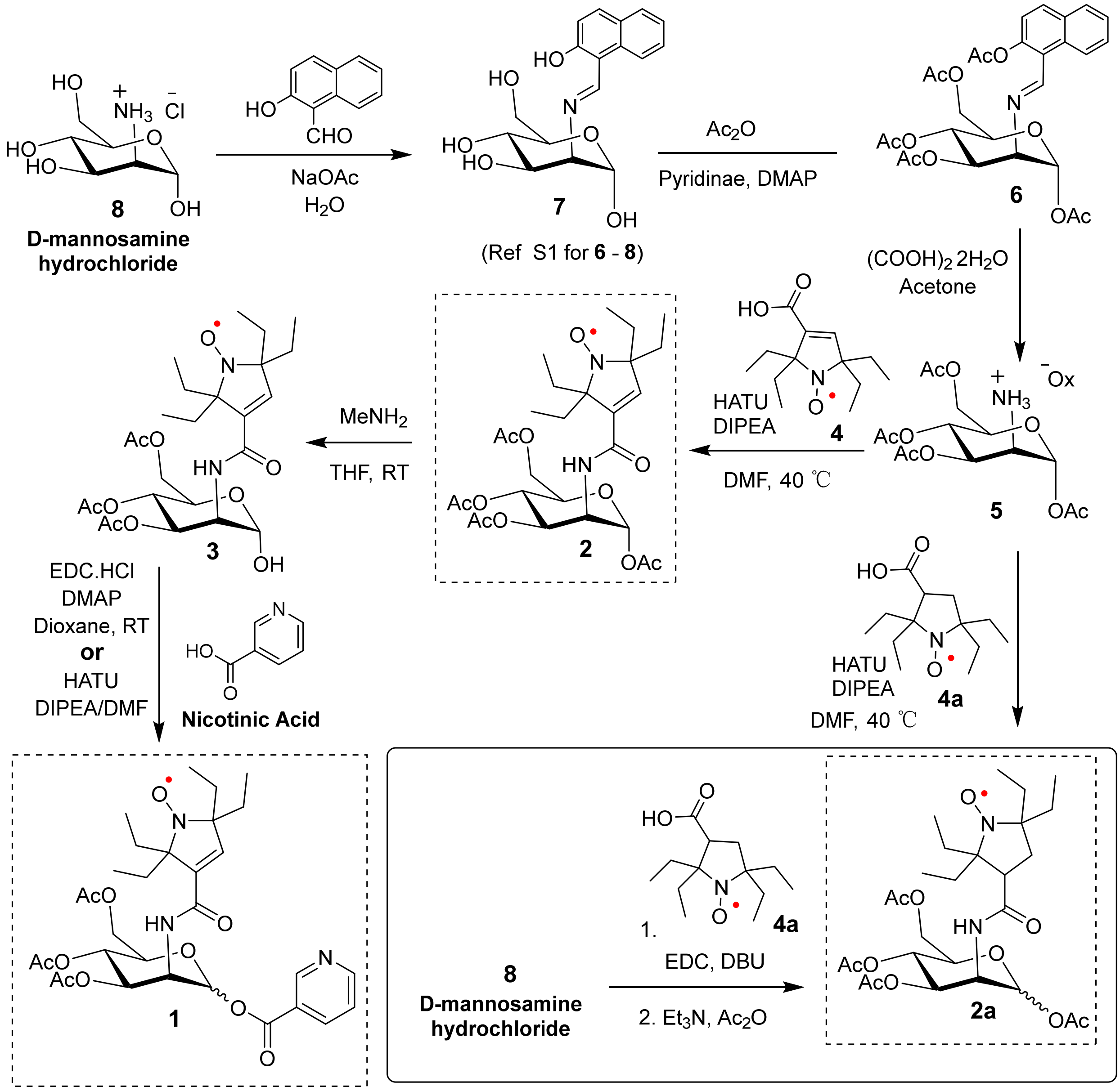

Scheme S1. Synthesis of agents **1**, **2**, and **2a**.

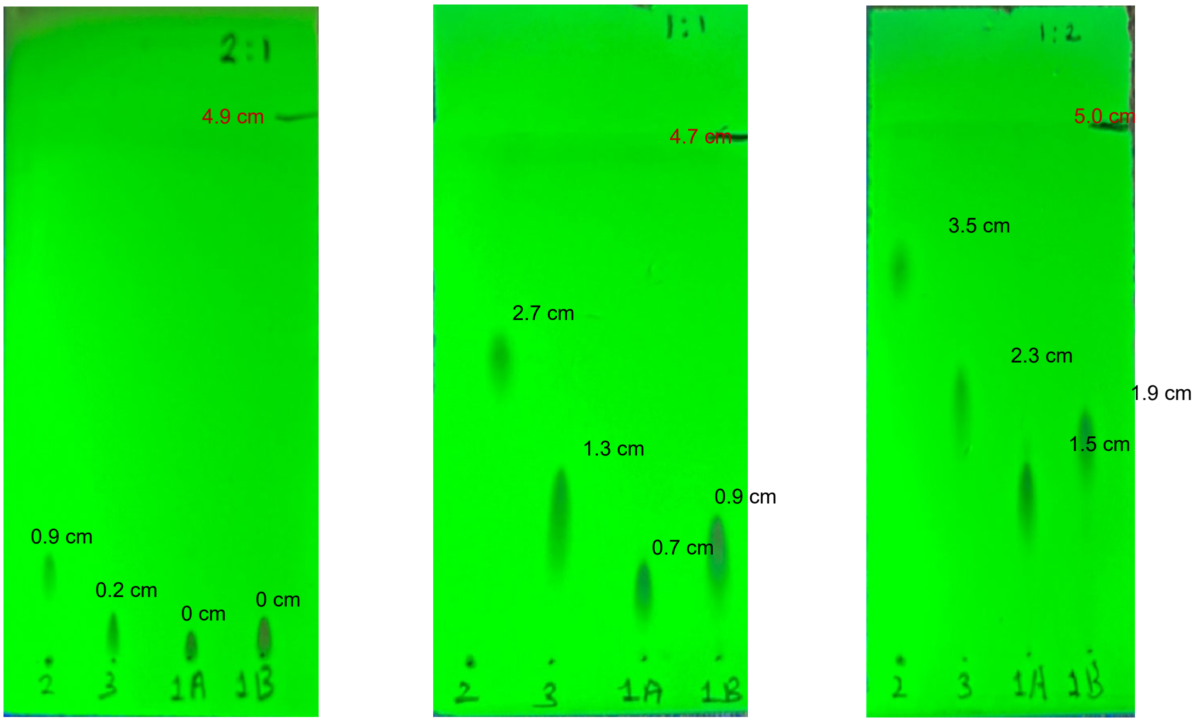

pentane/ethyl acetate (2/1) **pentane/ethyl acetate (1/1)** **pentane/ethyl acetate (1/2)**

TLC Sample	pentane/ethyl acetate (2/1) R_f	pentane/ethyl acetate (1/1) R_f	pentane/ethyl acetate (1/2) R_f
Compound **2**	0.18	0.57	0.70
Compound **3**	0.04	0.27	0.46
Compound **1** (Spot **A**)	0	0.15	0.30
Compound **1** (Spot **B**)	0	0.19	0.38

Figure S1. Examples of TLC plates (normal silica, shown under UV light irradiation) for compounds **1** – **3** and the summary of their R_f values.

1,3,4,6-Tetra-O-acetyl-2-amino-2-deoxy- ***α*** **-D-mannopyranose oxalate (5).** Compound **5** was synthesized according to a procedure reported in the literature, as outlined in Scheme S1; here, we describe the last step of that synthesis.[S1]

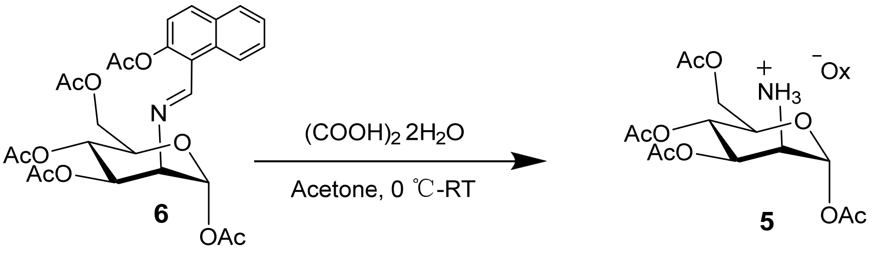

Label	Compound **6** (g/mmol/eq)	Oxalic acid dihydrate (g/mmol/eq)	Acetone (mL)	Yield (g/%)
CY-1-10	0.20/0.37/1.0	0.22/1.70/4.5	10	0.09/58
CY-1-26	0.30/0.55/1.0	0.31/2.46/4.5	15	0.13/53
CY-1-59	2.00/3.68/1.0	2.1/16.6/4.5	110	0.87/54
CY-2-14	1.06/1.95/1.0	1.11/8.78/4.5	50	0.53/62
CY-2-16	1.35/2.48/1.0	1.41/11.18/4.5	65	0.67/62

CY-1-59: Compound **6** (2.0 g, 3.68 mmol) was transferred to a clean, dry RBF containing a magnetic stir bar; acetone (ACS reagent grade, 75 ml) was added to the flask and cooled in an ice bath. Oxalic acid dihydrate (2.1 g, 16.6 mmol) was added to the reaction mixture followed by an additional amount of acetone (35 mL). The reaction mixture was stirred at 0 °C for 45 minutes; then, the reaction mixture was kept at room temperature for 1 hour. The reaction mixture was cooled again in an ice bath for 15 minutes, and the colorless crystalline solid was filtered in a clean sintered funnel. The solid was then washed with anhydrous diethyl ether (20 mL) and vacuum dried to obtain compound **5** as a white solid (0.87 g, 54% yield). 1H NMR (sample label: CY-1-26-H, 400 MHz, and *D*2O): δ 6.30 (s, 1H); 5.67 (dd, *J* = 9.6, 4.8 Hz, and 1H); 5.36 (t, *J* = 9.6 Hz, and 1H); 4.41 (d, *J* = 10.8 Hz, and 2H); 4.19 (d, *J* = 10.4 Hz, and 1H); 4.06 (d, *J* = 3.2 Hz, and 1H); 2.24 (s, 3H); 2.16 (s, 3H); and 2.13 (s, 6H). It was observed that the previously reported 1H NMR signals of compound **5** were about 2 ppm more downfield than those in the 1H NMR spectrum reported in this study. According to previously reported references, [S1] NMR spectra of compound **5** are as follows: 1H NMR (*D*2O:C*D*3O*D*: 2:1, and 400 MHz): δ 4.76 (d, 1H, and J = 1.7); 4.07 (dd, 1H, J = 9.6, and J = 4.7); 3.83 (t, 1H, and J = 9.6); 2.86 (dd, 1H, J = 12.2, and J = 5.0), 2.80 (ddd, 1H, J = 9.9, J = 5.0, and J = 2.3); 2.62 (dd, 1H, J = 12.2, and J = 2.1); 2.48 (dd, 1H, J = 4.7, and J = 1.8); and 0.70, 0.62, 0.59, 0.58 (4s, 3H each). 13C NMR (*D*2O:C*D*3O*D*: 2:1, 100 MHz): δ 174.3, 173.2, 172.7, 171.5, 166.2, 90.6, 70.9, 68.5, 66.2, 62.9, 51.8, 21.0, 20.9 (2C), and 20.8.

1,3,4,6-Tetra-*O*-acetyl-mannosamine-*N*-nitroxide 2: the coupling reaction of the mannose sugar and pyrroline nitroxide was performed using the method reported in the literature [S2].

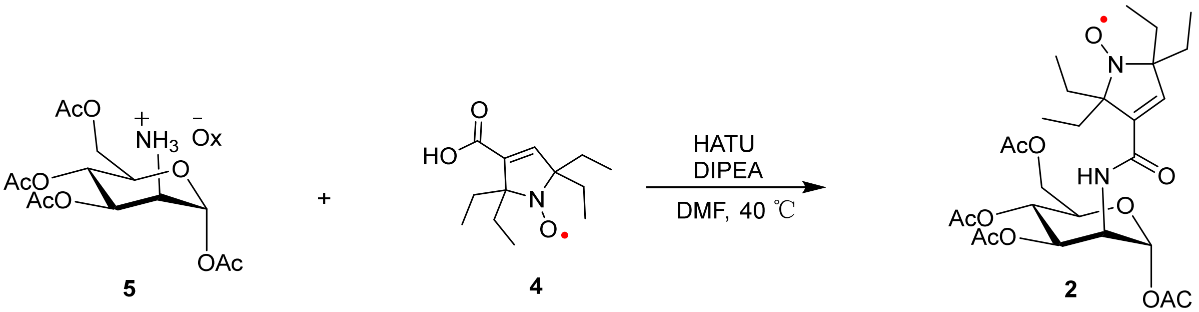

Run	Compound **5** (mg/mmol/eq)	Compound **4** (mg/mmol/eq)	HATU (mg/mmol/eq)	DIPEA (µL/mmol/eq)	DMF (mL)	Yield (mg/%)
CY-2-23	17.1/0.039/1.2	7.8/0.032/1.0	14.8/0.039/1.2	20/0.117/3.6	0.4	16.3/88
CY-2-26	105/0.240/1.2	48.4/0.201/1.0	91.3/0.240/1.2	100/0.574/2.8	1.0	94.2/83
SD-1-11	105/0.240/1.2	48.4/0.201/1.0	92.0/0.241/1.2	100/0.574/2.8	1.5	53.1/45
SD-1-13	105/0.240/1.2	48.7/0.203/1	92.7/0.241/1.2	150/0.861/4.2	1.5	76.7/65
SD-1-34	105.6/0.241/1.2	48.9/0.203/1	92.9/0.244/1.2	150/0.861/4.2	1.5	77.2/66
SD-1-36	105/0.240/1.2	48.5/0.202/1	92.0/0.242/1.2	150/0.861/4.2	1.5	62.3/53
SZ-4-66	17.5/0.039/1.2	7.8/0.032/1.0	14.8/0.039/1.2	30/0.172/5.2	0.4	15.0/83
SZ-4-67	105/0.240/1.2	48.4/0.201/1.0	92.0/0.241/1.2	150/0.861/4.2	1.5	84.7/72

CY-2-26: Compound **5** (105.0 mg, 0.240 mmol) was loaded in Schlenk tube 1 and evacuated under high vacuum overnight and then charged with argon. HATU (91.3 mg, 0.240 mmol) was loaded to Schlenk tube 2. Pyrroline nitroxide **4** (48.4 mg, 0.201 mmol), which was synthesized according to the published literature [S3], was added to Schlenk tube 2, evacuated under a high vacuum line overnight, and then subsequently charged with argon. Anhydrous DMF (1.0 mL) was added to the tube containing compound **4** and HATU and stirred at room temperature. DIPEA (100 µL, 0.574 mmol) was added to the above reaction solution under argon. After stirring for 1 h, the above reaction solution was added to Schlenk tube 1, which contained compound **5**, and then stirred under argon at room temperature for 60 h. The reaction mixture was transferred to a vial and dried under a flow of N_2. The crude mixture was then dried under high vacuum. The crude mixture was then purified via column chromatography using pentane/ ethyl acetate (1/1, R_f = 0.57) to obtain compound **2** as a pale yellow waxy solid (94.2 mg, 83%). EPR (sample label: CY-2-26-col, 1 mM in chloroform, and spin concentration: ~100%). ^{1}H NMR (sample label: CY-2-23-fil-H, 700 MHz, and 0.18 M in acetone-d_6): δ 7.72 (s, 1H); 6.05 (s, 1H); 5.33

(s, 1H); 5.25 (s, 1H); 4.85 (s, 1H); 4.16 (s, 2H); 4.02 (s, 1H); 2.19 (s, 3H); 2.01 (s, 3H); 1.97 (s, 3H); and 1.95 (s, 3H). HR-MS-EI (m/z): For sample CY-2-26-col, $[M]^+$ calculated for $C_{27}H_{41}N_2O_{11}$ 569.2710 was found to be 569.2684 (-4.6 PPM, RA 100%), and $[M+H]^+$ calculated for $C_{27}H_{42}N_2O_{11}$ 570.2789 was found to be 570.2724 (-11.3 PPM, RA 37%).

SZ-4-67: Compound **4** (48.4 mg, 0.201 mmol) was added to HATU (92 mg, 0.241 mmol) in a Schlenk tube and evacuated under a high vacuum line overnight. In a separate vial, compound **5** (105 mg, 0.2397 mmol) was evacuated. The Schlenk tube was charged with argon. Under an argon flow, anhydrous DMF (1.5 mL) and DIPEA (150 μL, 0.861 mmol) were added to the Schlenk tube and then stirred at room temperature for 30 min. Compound **5** was added to the mixture in one portion under argon and stirred at 40 ℃ for 28 h. The yellow homogeneous solution was cooled to room temperature. The crude mixture was then transferred to a vial and concentrated under a flow of N_2. The purification of the crude was performed via column chromatography on a silica gel using pentane/ethyl acetate (1/1, R_f = 0.57) to obtain compound **2** as a pale-yellow solid foam, (84.7 mg, 72%). EPR (sample label: SZ-4-67, 0.922 mM in chloroform/toluene (1:4), and spin concentration: 94%).

1-Hydroxy-3,4,6-*O*-acetyl-mannosamine-*N*-nitroxide 3: compound **3** was synthesized from compound **2** with methylamine using the method for other carbohydrate derivatives reported in the literature [S4].

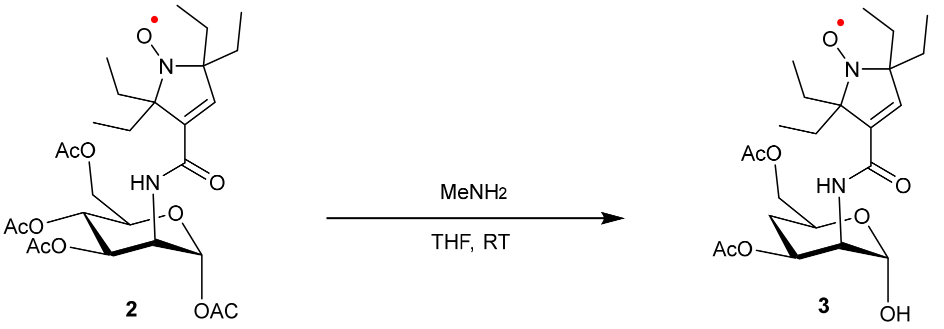

Run	Compound **2** (mg/mmol/eq)	$MeNH_2$ (mg/mmol/eq)	THF (mL)	Yield (mg/%)

SD-1-4	10/0.0176/1	0.709/0.0228/1.3	0.05	5.3/57
SD-1-8	5/0.00878/1	0.354/0.0114/1.3	0.02	1.6/35
SD-1-12	30/0.0526/1	2.127/0.0685/1.3	0.26	17.5/63
SD-1-14	30/0.0526/1	2.127/0.0685/1.3	0.26	16.8/61
SD-1-22	70.7/0.124/1	5.013/0.161/1.3	0.62	31.6/48
SD-1-23	59.6/0.105/1	4.228/0.136/1.3	0.52	28.4/51
SD-1-35	77.2/0.136/1	5.475/0.176/1.3	0.68	22.3/31
SD-1-39	60.9/0.107/1	4.317/0.139/1.3	0.54	37.4/66

SD-1-39: Compound **2** (60.9 mg, 0.107 mmol) was added to a Schlenk tube and evacuated under a high vacuum line for 1 hour. Methylamine (4.3 mg, 0.139 mmol) in THF distilled with sodium/benzophenone (0.54 mL) was added under argon and stirred at room temperature for 17 h. The crude product was concentrated through evaporation of THF and then purified via column chromatography on a silica gel using pentane/ethyl acetate (1/1, $R_f = 0.27$) to obtain compound **3** as yellow oil, (37.4 mg, 66% yield). ^{1}H NMR (sample label: SD-1-35-bottomspot, 700 MHz, 0.027 M in $CDCl_3$): δ 6.15 (br, 1H); 5.46 (s, 1H); 5.21 (s, 1H); 4.88 (s, 1H); 4.32 (s, 2H); 4.14 (s, 1H); 2.13 (s, 3H); 2.09 (s, 3H); 2.01 (s, 3H); and 2.01 (s, 3H). ^{13}C NMR (sample label: SD-1-35-bottomspot, 700 MHz, and 0.027 M in $CDCl_3$): δ 170.62, 170.27, 170.16, 169.93, 169.78, 72.58, 69.93, 68.13, 65.50, 62.48, 62.37, 53.81, 50.92, 38.72, 31.98, 29.42,29.32, 21.45, 21.38, and 20.83. Note: The ^{1}H singlet at 2.83 ppm is suspected to be methylamine and is used as the reagent. According to reference [S5], the ^{1}H and ^{13}C NMR chemical shifts for methylamine in D_2O are 2.59 and 27.52 ppm, respectively. HR-MS-ES (m/z) (sample label: SD-1-40-bottom): $[M+Na]^+$ calculated for $C_{25}H_{39}N_2O_{10}Na$ 550.2502 was found to be found 550.2493 (-1.6 ppm, RA 100%), and for $^{12}C_{24}{}^{13}CH_{39}N_2O_{10}Na$ 551.2536, it was found to be 551.2524 (-2.2 PPM, RA 27%). Note: SD-1-40-bottom was the sample label for the product from run SD-1-39.

Mannosamine Nicotinate 1: Compound **1** was synthesized from compound **3** using nicotinic acid, 4-dimethyl aminopyridine (DMAP), and 1-ethyl-3-3-dimethylaminopropylcarbodiimide (EDC.HCl) according to the procedure in the reported literature [S5]. In addition, HATU-mediated coupling was attempted (run:

SD720).

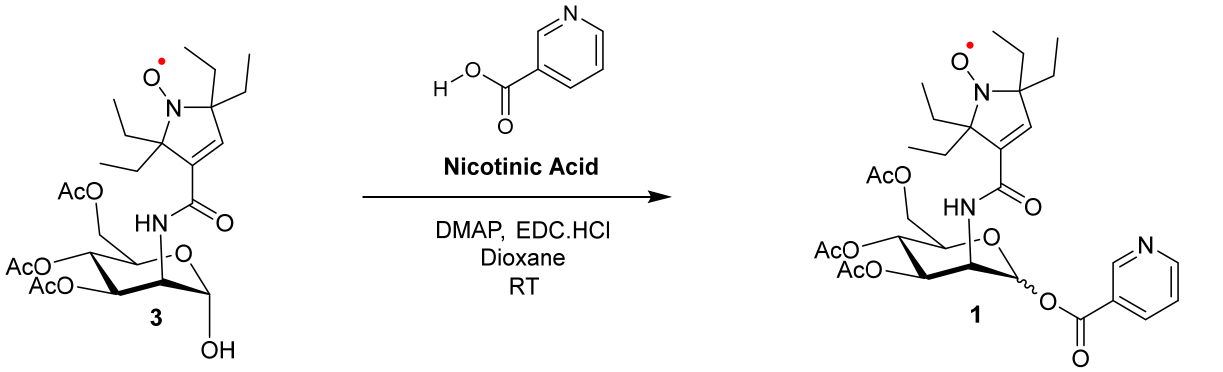

Run	Compound **3** (mg/mmol/eq)	Nicotinic acid (mg/mmol/eq)	DMAP (mg/mmol/eq)	EDC. HCl (mg/mmol/eq)	Dioxane (mL)	Yield (mg/%)	Spot3/A (mg)	Spot 4/B (mg)
SD-1-16	10/0.0189/1	4.65/0.0378/2	1.16/0.0095/0.5	10.9/0.057/3	0.2	5.1/43	2.5	2.6
SD-1-17	10/0.0189/1	4.65/0.0378/2	1.16/0.0095/0.5	10.9/0.057/3	0.2	8.2/69	3.7	4.5
SD-1-26	60/0.1138/1	28.02/0.228/2	6.95/0.057/0.5	65.4/0.34/3	1.0	31.4/44	13.4	18.0
SD-1-42	35.5/0.067/1	16.57/0.135/2	4.13/0.037/0.5	38.7/0.20/3	0.6	35.1/83	14.9	20.2
Run	Compound **3** (mg/mmol/eq)	Nicotinic acid (mg/mmol/eq)	DIPEA (mg/mmol/eq)	HATU (mg/mmol/eq)	DMF (mL)	Yield (mg/%)	Spot3/A (mg)	Spot 4/B (mg)
SD720[a]	6.5/0.0123/1	3.0/0.0243/2.0	4.8/0.037/3.0[a]	8.3/0.022/1.8[a]	0.1[a]	5.6/72	3.2	2.4

[a] In run SD720, DIPEA, HATU, and DMF are used.

SD-1-42: Compound **3** (35.5 mg, 0.067 mmol) was added to a Schlenk tube and evacuated under a high vacuum line for 1 h. Nicotinic acid (17.5 mg, 0.142 mmol), DMAP (4.22 mg, 0.035 mmol), and EDC.HCl (38.7 mg, 0.202 mmol) were added to the Schlenk tube. Under argon, dioxane (0.6 mL) was added. The heterogenous suspension turned to a yellow homogeneous solution after stirring for 1 h. The reaction mixture was stirred at room temperature for 17 h. The solvent was then evaporated under a flow of N_2 to obtain the crude product as yellow oil. The purification of the crude was performed with a preparative TLC plate using regular silica and pentane/ethyl acetate (1/1, R_f = 0.15 and 0.19 for spots **A** and **B**, respectively). Diastereomers of compound **1** were isolated and dried under a high vacuum line overnight. Diastereomeric spots **A** (14.9 mg) and **B** (20.2 mg) of compound **1** (Fig. S1) were obtained as yellow oil, (35.1 mg, 82% yield). EPR (sample label: SD-1-18-spotA, 0.829 mM in dichloromethane/toluene (1:4), spin

concentration: 85%, sample label: SD-1-18-spotB, 0.438 mM in dichloromethane/toluene (1:4), and spin concentration: 89%). ^{1}H NMR (sample label: SD-1-42-spotA, 700 MHz, and 0.041 M in $CDCl_3$): δ 9.35 (s, 1H); 8.91 (s, 1H); 8.40 (d, *J* = 5.6 Hz, 1H); 7.52 (s, 1H); 6.41 (s, 1H); 5.50 (s, 1H); 5.36 (s, 1H); 5.06 (s, 2H); 4.37 (s, 1H); 4.21 (s, 1H); 4.13 (s, 1H); 2.13 (s, 6H); and 2.06 (s, 3H). ^{13}C NMR (sample label: SD-1-42-spotA, 700 MHz, and 0.041 M $CDCl_3$): δ 210.95, 170.39, 169.97, 169.69, 162.70, 154.75, 151.31, 137.66, 124.67, 123.93, 93.08, 70.77, 69.69, 69.58, 65.05, 61.91, 53.98, 48.67, 32.00, 29.78, 29.44, 21.39, and 20.87. ^{1}H NMR (sample label: SD-1-42-spotB, 700 MHz, and 0.053 M $CDCl_3$): δ 9.20 (s, 1H); 8.84 (s, 1H); 8.27 (s, 1H); 7.41 (s, 1H); 6.20 (s, 1H); 5.27 (s, 1H); 5.18 (s, 2H); 4.37 (s, 1H); 4.20 (s, 1H); 3.98 (s, 1H); 2.12 (s, 6H); and 2.06 (s, 3H). ^{13}C NMR (sample label: SD-1-42-spotB, 700 MHz, and 0.053 M in $CDCl_3$): δ 210.96, 170.41, 170.08, 169.72, 162.82, 154.68, 151.46, 137.73, 124.65, 123.71, 91.85, 73.73, 72.04, 69.58, 65.10, 61.79, 53.96, 48.99, 31.98, 29.78, 29.43, 21.53, 21.16, and 20.88.

HR-MS-ES (m/z): (sample label: SD-1-42-spot3/A) $[M+Na]^+$ calculated for $C_{31}H_{42}N_3O_{11}Na$ 655.2717 was found to be 655.2697 (-3.1 ppm, RA 100%), and for $^{12}C_{30}{}^{13}CH_{42}N_3O_{11}Na$ 656.2751, it was found to be 656.2729 (-3.4 ppm, RA 33%).

HR-MS-ES (m/z): (label: SD-1-42-spot4/B) $[M+Na]^+$ calculated for $C_{31}H_{42}N_3O_{11}Na$ 655.2717 was found to be 655.2721 (0.6 ppm, RA 100%), and for $^{12}C_{30}{}^{13}CH_{42}N_3O_{11}Na$ 656.2751, it was found to be 656.2762 (1.7 ppm, RA 37%).

SD720: Compound **3** (6.5 mg, 0.0123 mmol) was added to a Schlenk tube and evacuated under a high vacuum line overnight. In a different Schlenk tube, nicotinic acid (3.0 mg, 0.0243 mmol) and HATU (8.3 mg, 0.022 mmol) were added and evacuated under a high vacuum line for 1 h. Under argon, DMF (0.1 mL) and DIPEA (4.8 mg, 0.037 mmol) were added. The clear homogeneous solution was stirred at room temperature for 1 h. To the reaction mixture, compound **3** in DMF (0.5 mL) was added and stirred at 40 °C for 17 h. The solvent was then evaporated under a flow of N_2 to obtain crude as yellow oil. Crude was applied through a short silica plug, regular silica, and pentane/ethyl acetate

(1/2, R_f = 0.30 and 0.38, for spots **A** and **B**, respectively, Fig. S1) to remove polar impurities. The column fraction (SD720col1, 14.6 mg) was then further purified with a preparative TLC plate using regular silica and pentane/ethyl acetate (1/2). Diastereomers of compound **1** were isolated and dried under a high vacuum line overnight. Diastereomeric spots **A** (3.24 mg) and **B** (2.37 mg) of compound **1** were obtained as yellow oil, (5.61 mg, 72% yield).

1,3,4,6-Tetra-*O*-acetyl-mannosamine-*N*-nitroxide 2a: compound **2a** was synthesized from either compound **8** using 4-dimethyl aminopyridine (DBU) and 1-ethyl-3-3-dimethylaminopropylcarbodiimide (EDC.HCl), followed by per-*O*-acetylation, or by using HATU-mediated coupling with compound **5** (same conditions as for compound **2**).

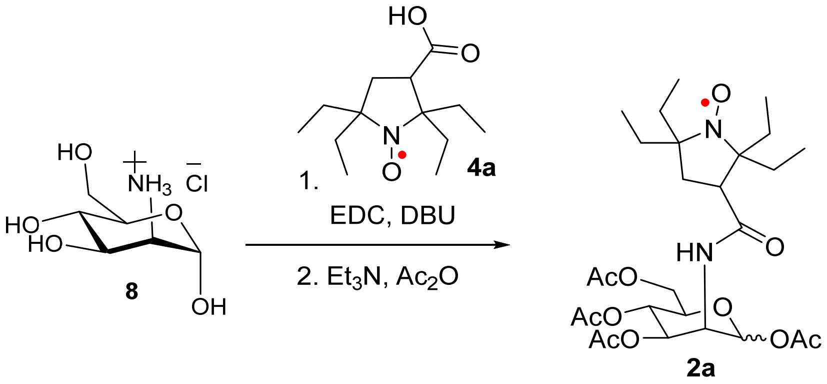

Run[a]	Mannosamine **8** (mg/mmol)	Nitroxide **4a** (mg/mmol)	DMF (mL)	DBU (mL/mmol)	EDC (mg/mmol)	Ac_2O (mL/mmol)	Et_3N (mL/mmol)	Yield (mg/%)
JTP-7-09	14/0.064	12/0.050	1.2	0.030/0.201	31/0.161	0.4/4.23	0.68/4.88	4/14
JTP-7-48	51/0.235	49/0.201	2.4	0.105/0.702	115/0.598	1.5/15.9	2.5/17.9	8.2/7[b]
**JTP-7-72**	56.4/0.262	65.2/0.269	2.5	0.150/1.00	128.0/0.666	1.7/18.3	2.8/19.7	6.7/6
**JTP-7-74**	54.8/0.254	60.8/0.251	2.5	0.114/0.762	128.6/0.669	1.7/18.0	2.7/19.4	18.1/17[c]
JTP10-64	54.6/0.253	63.0/0.260	2.4	0.13/0.870	140.0/0.729	1.7/18.0	2.7/19.4	17.4/17
Run	Compound **5** (mg/mmol/eq)	Nitroxide **4a** (mg/mmol/eq)	DMF (mL)	DIPEA (µL/mmol/eq)	HATU (mg/mmol/eq)	Yield (mg/%)		
SD729	58/0.132/1.3	25/0.103/1	0.8	60/0.344/3.3	51.0/0.134/1.3	42.9/73		

[a] Only successful runs that resulted in isolation of **2a** are shown. [b] Spin concentration of 102% (in chloroform). [c] Spin concentration of 74%.

JTP-7-74: *D*-Mannosamine hydrochloride **8** (55 mg, 0.25 mmol) was dried under high vacuum at 55 °C for two days. Pyrrolidine nitroxide **4a** (61 mg, 0.25 mmol) and

EDC (129 mg, 0.67 mmol) were added and then evacuated for 10 min. DMF (2.5 mL) and DBU (0.1 mL, 0.76 mmol) were added under a flow of argon, resulting in a yellow solution. After nine days, this solution was slightly darker and more orange; then, triethylamine (2.7 mL, 19 mmol) and acetic anhydride (1.7 mL, 18 mmol) were added. After 12 h, the reaction mixture was concentrated under a stream of nitrogen. After dissolving in ethyl acetate (50 mL), the resulting mixture was washed with saturated aqueous sodium bicarbonate (2 × 50 mL). The aqueous layers were extracted with chloroform (7 × 20 mL) and combined with the ethyl acetate. After drying over magnesium sulfate and evaporating, a brown oil (1.5 g) was obtained as the crude product. To remove the remaining DMF, the mixture was dissolved in ethyl acetate (80 mL) and washed with brine (4 × 10 mL); the brine was extracted with ethyl acetate (2 × 5 mL), washed with saturated aqueous sodium bicarbonate (2 × 10 mL), washed again with brine (2 × 10 mL), dried over magnesium sulfate, and evaporated to yield the crude product (189 mg). Washing with brine first seems to help keep the color (possibly due to the product) out of the water layer. The crude was filtered through a silica plug using chloroform/methanol/acetone (48/1/1); a pale brown band stayed at the origin and did not move with the polar solvent (7% methanol in dichloromethane); evaporation yielded a filtered crude product (41 mg). The filtered crude product was purified through preparative TLC (silica gel with chloroform/methanol/acetone (48/1/1) to yield the product **2a** (18.1 mg, 17%)).

SD729: Compound **4a** (25.0 mg, 0.103 mmol) and HATU (51.0 mg, 0.134 mmol) were loaded to a Schlenk tube and evacuated under a high vacuum line overnight. In a separate vial, compound **5** (58 mg, 0.132 mmol) was evacuated under a high vacuum line overnight. The Schlenk tube was degassed with argon. Anhydrous DMF (0.8 mL) and DIPEA (60 μL, 0.344 mmol) were added under argon and stirred at room temperature. After stirring for 1 hour, compound **5** was added to the Schlenk tube and stirred at 40 ℃ for 18 hours. The reaction mixture was transferred to a vial and dried under a N_2 flow.

The crude mixture was then purified via column chromatography using pentane/ethyl acetate (1/1, R_f = 0.60). Compound **2a** was isolated and evacuated under a high vacuum line overnight to obtain a pale yellow waxy solid (42.9 mg, 73%). ^{1}H NMR (sample label: SD729Fr1, 700 MHz, and 0.018 M in $CDCl_3$): δ 6.07 (s, 1H); 5.97 (s, 1H); 5.61 (s, 1H); 5.49 (s, 1H); 5.23 (s, 2H); 4.95 (s, 1H); 4.77 (s, 1H); 4.34 (s, 1H); 4.27 (s, 1H); 4.08 (s, 4H); 2.2 (s, 6H); 2.09 (s, 12H); and 2.06 (s, 6H).^{13}C NMR (sample label: SD729Fr1, 700 MHz, and 0.018 M in $CDCl_3$): δ 170.43, 170.40, 169.94, 169.77, 169.67, 169.42, 168.26, 168.16, 91.64, 91.55, 70.46, 70.29, 70.18, 69.15, 65.18, 64.73, 61.83, 61.57, 29.73, 29.32, 21.47, 21.37, 21.01, 20.96, 20.89, 20.73, and 20.61. HR-MS-ESI (m/z) (sample label: SD729Fr1): $[M+Na]^+$ calculated for $C_{27}H_{43}N_2O_{11}Na$ 594.275906 was found to be 594.275783 (0.2 ppm, RA 100%).

Preparation of samples of 2a for cell incubation: Prior to incubation with Jurkat cells, the sample (label: JTP-7-74) was dissolved in EtOH (0.65 mL), filtered through a 0.2 μm PTFE syringe filter, and then dried (under high vacuum). Analogously, sample JTP-7-72 was treated in the same way.

Reduction kinetics of nitroxide 2a with ascorbate: Equal volumes of 0.4 mM nitroxide radical **2a** (sample label: JTP-7-48) and 8 mM ascorbate, both in PBS containing 0.1 mM DTPA, are vortexed for a few seconds, and the resultant solution of 0.2 mM in **2a** and 4 mM in ascorbate is rapidly loaded into a 0.6 mm ID EPR quartz capillary. EPR spectra at 295.0 K (label: JP835r0 – r40) are obtained every 60 s in the initial decay period (20 min), which is used to obtain the second-order rate constant $k_2 \approx 0.001$ M^{-1} s^{-1}, derived from pseudo-first-order rate constants of 5.80×10^{-6} and 4.07×10^{-6} s^{-1}; the pseudo-first-order rate constants are obtained by measuring the peak height or singly integrated peak height [S3]. After the initial 1.5 h, there is no detectable decay of the radical (up to 3 h).

2. Summary of *in-cell* EPR spectroscopy.

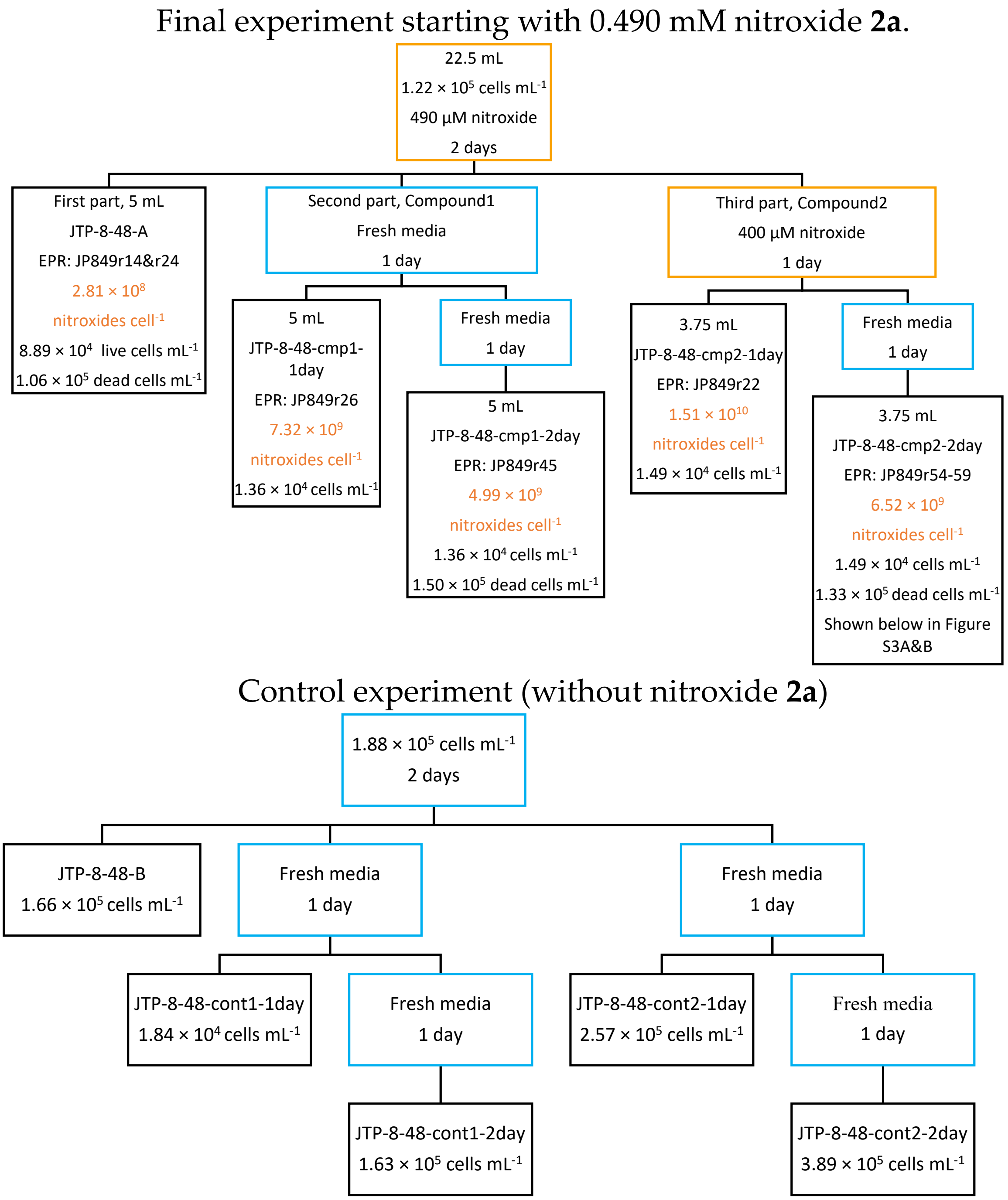

Fig. S2. Flow charts illustrating the design of the *in-cell* EPR experiment with Jurkat cells. Orange and blue squares denote the nitroxide-containing and nitroxide-free media, respectively. Sample labels indicate the sample type: A and B denote the base sample and the control, respectively. Numbers after "cmp" and before "day" denote the number of incubation periods with nitroxide and the number of days after splitting of

the base sample. In the control experiment, EtOH (0.15 mL) is added to the media in place of **2a** in EtOH.

In the final experiment, nitroxide **2a** (sample label: JTP-7-74/JTP-7-72) in EtOH (0.15 mL) is added to the media containing dilute Jurkat cells (1.22×10^5 cells/mL), thus obtaining 0.49 mM nitroxide **2a** (based on the quantitative EPR spectra prior to incubation). Following incubation for two days (Fig. S2), the sample is divided into three parts, with the first part being the work-up for analysis. In the second part, the cells are centrifuged; media are removed, suspended in media containing no nitroxide, and then incubated for one day. The cells in the third part are centrifuged, and the media are removed, suspended in media containing 400 μM nitroxide, and then incubated for one day. For the second and third parts of the cell sample, one sub-part is worked up for analysis and the other sub-part is centrifuged; media are removed, suspended in fresh media with no nitroxide, and then incubated for one more day, after which they were worked up for analysis.

The work-up for analysis involves counting the cells (Trypan blue), followed by five iterations of centrifugation, the removal of media, and suspension in fresh PBS buffer. Five washes employed are more than sufficient because after four washes, the radical concentration of the supernatant PBS (media) is reduced to within the measurement error of fresh PBS buffer (at 243 K), indicating no additional radicals could be removed by washing. Finally, the cells were suspended in a minimal volume of PBS buffer and transferred to a 5 mm OD quartz EPR tube for spin counting.

In addition, for the spectrum at 295 K, a portion of the final third part of the cell sample (label: JTP-8-48-cmp2-2day) is loaded with extra PBS buffer into four stoppered 0.6 mm ID EPR quartz capillaries, which are then placed into a 5 mm OD EPR quartz tube (Fig. S3B). Even though the height of the PBS buffer/cell mixture is ca. 5 cm, which exceeds the height of the EPR cavity, an excellent Q-value of 3100 is obtained, and the tube is positioned so that the mid part of the sample corresponds to the center of the cavity. Note that the alternative spectra at 295 K, using 5 mm OD EPR quartz tubes, which are partially inserted into the cavity, to provide $Q \approx 1500$, give spectra with much lower S/N ratios for the same sample (label: JTP-8-48-cmp2-2day) and other samples with lower spin concentrations (Fig. S3).

Spin counting was accomplished by comparing the radical concentration in cell pellets to a solid sample of TEMPOL of known mass. Solid TEMPOL was loaded into an approx. 5 mm long tube made from a coffee straw sealed with parafilm to prevent electrostatic forces from scattering the pieces of solid TEMPOL. The straw vessel is lowered into a 5 mm O.D. quartz EPR tube and positioned such that the entire TEMPOL sample is in the instrument cavity and would be accounted for in the measurement. Jurkat cells are suspended in a minimal volume of PBS buffer and transferred to a 5 mm OD quartz EPR tube. The resultant mixture possesses smaller dimensions compared to the EPR cavity, implying that the entire sample is analyzed and any small differences in

PBS volume are inconsequential. Spectra are acquired at 243 K to allow for the use of 5 mm OD quartz tubes to maximize the signal-to-noise ratio, e.g., Fig. S3A. Because typical cavity Q-values are 1600 for cells and 3800-4100 for solid TEMPOL, this implies that the actual spin concentrations of nitroxides on cells are significantly higher (by a factor of ca. 2) than the reported ones in Fig. S2.

An example for the computation of the number of nitroxide radicals per cell is provided below for sample JTP-8-48-cmp1-1day. The spin concentration measurement gives 8.26×10^{-7} mmol = 8.26×10^{-10} mol nitroxides on cells; thus, the total number of nitroxides on cells is 8.26×10^{-10} mol * 6.022×10^{23} = 4.97×10^{14}. Because the pelleted cells used for the spin concentration measurement are prepared from a 5 mL culture containing 1.36×10^{4} cells mL^{-1}, the number of nitroxide radicals per cell is $4.97 \times 10^{14} \div (1.36\times10^{4} \times 5) = 7.32 \times 10^{9}$.

For the same sample, starting with a spin concentration of 8.26×10^{-7} mmol, a volume of 5 mL = 0.005 L, and a concentration of radicals in the initial culture of 0.49 mM, we can estimate the efficiency of incorporating nitroxide radicals onto cells: $(8.26 \times 10^{-7} \div 0.005) \div 0.49 = 3.4 \times 10^{-4} = 0.034\%$. In other words, approximately 1 out of about 3000 nitroxide radicals is incorporated into cells.

According to the spin counting results, the greatest concentration of nitroxides per cell was observed after three days of total incubation. For the cells which were incubated with nitroxide the entire time, this is not surprising. However, incubation in nitroxide-free media for one day after the initial incubation period also increased the cellular nitroxide concentration. This result is due to the way the media is exchanged. Cells are pelleted, and the media are removed with a pipette, but a significant volume of media remains; then, new media are added. Apparently, the residual concentration of nitroxide is still sufficient for the cells' metabolism to increase the nitroxide per cell count.

Cell viability tends to decrease with incubation time; that is, after the initial 2 d incubation, the ratio of live-to-dead cells is about 1:1; however, an additional incubation of 2 d, even with fresh media without nitroxide, decreases the ratio to about 1:10.

Spectral simulations are carried out using the EasySpin *pepper* suite (spectra at 243 K) and the *chili* suite (spectra at 295 K).

Table S1. Summary of spectral simulations presented in Figures S3 and S4.

Fig.	T (K)	Freq (GHz)	rmsd	Content (%)	τ_{cor}	A_{zz}[a] (MHz)	g_{zz}	g_{yy}	g_{xx}	Lwpp (mT)	H-strains (MHz) zz	yy	xx
S3A	243	9.6510	0.1030	100	rigid	96.5	2.0026	2.0064	2.0087	na	28.9±0.1	25.6±0.3	32.3±0.9
S3B	295	9.6464	0.02599	94	21.6±0.0 ns	96.9	*b*	*b*	*b*	0.635			
				6	8.4±0.5 ps	97.4	*b*	*b*	*b*	1.123			
S3C	295	9.6518	0.04115	100	15.3±0.0 ps	96.9	2.0030	2.0064	2.0073	0.448			
S4	243	9.6506	1124	100	rigid	96.4	2.0026	2.0066	2.0086	na	29.0±0.1	24.8±0.3	33.6±0.8
S4	295	9.6579	0.04084	97	21.6±0.0 ns	94.4	*b*	*b*	*b*	0.545			
				3	8.7±1.8 ps	98.0	*b*	*b*	*b*	0.361			

[a] Values of ^{14}N tensor component A_{zz} are essentially equal to 97 MHz; other components of the ^{14}N A-tensor were fixed to A_{yy} = 16.8 and A_{xx} = 15.0 MHz to minimize on over-parametrization of the fit. [b] For S3B and S4 (at 295 K), g-tensor components were fixed to g_{xx} = 2.0078, g_{yy} = 2.0068, and g_{zz} = 2.00235 in the final fit iteration to minimize on over-parametrization; values for g-tensor components are not corrected.

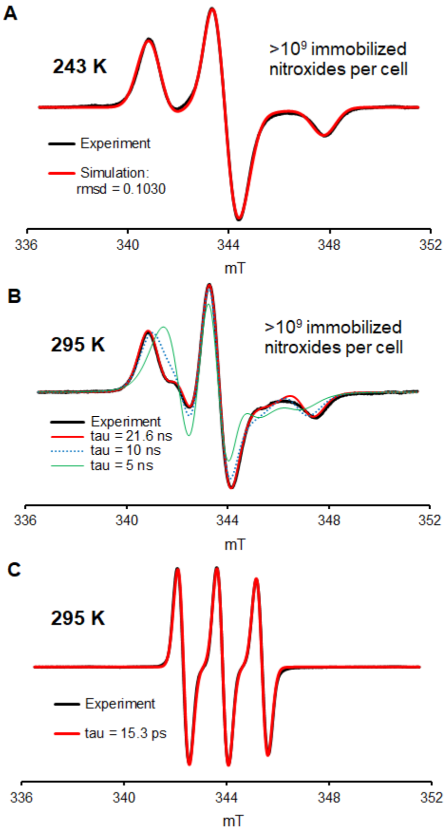

Fig. S3. Enlarged Figure 2 in the main text with a more detailed figure caption. EPR (X-band) spectra of agent **2a** in Jurkat cells. (A) Spectrum (EPR label: JP849r59, sample label: JTP-8-48-cmp2-2day) of washed cells at 243 K, which were used for the quantitation of nitroxide radicals. (B) Spectrum (EPR label: JP849r60, sample label: JTP-8-48-cmp2-2day) of washed cells resuspended in extra PBS buffer at 295 K showing the

predominant presence of highly immobilized nitroxide radicals, with a slow-to-fast component ratio = 1.00/0.06. (C) Spectrum (EPR label: JP845r28, sample label: JTP-8-44-B) of **2a** in cell media at 295 K. Spectral simulations are carried out with the EasySpin *pepper* (A) and *chili* (B and C) suites; spectral parameters are summarized in Table S1.

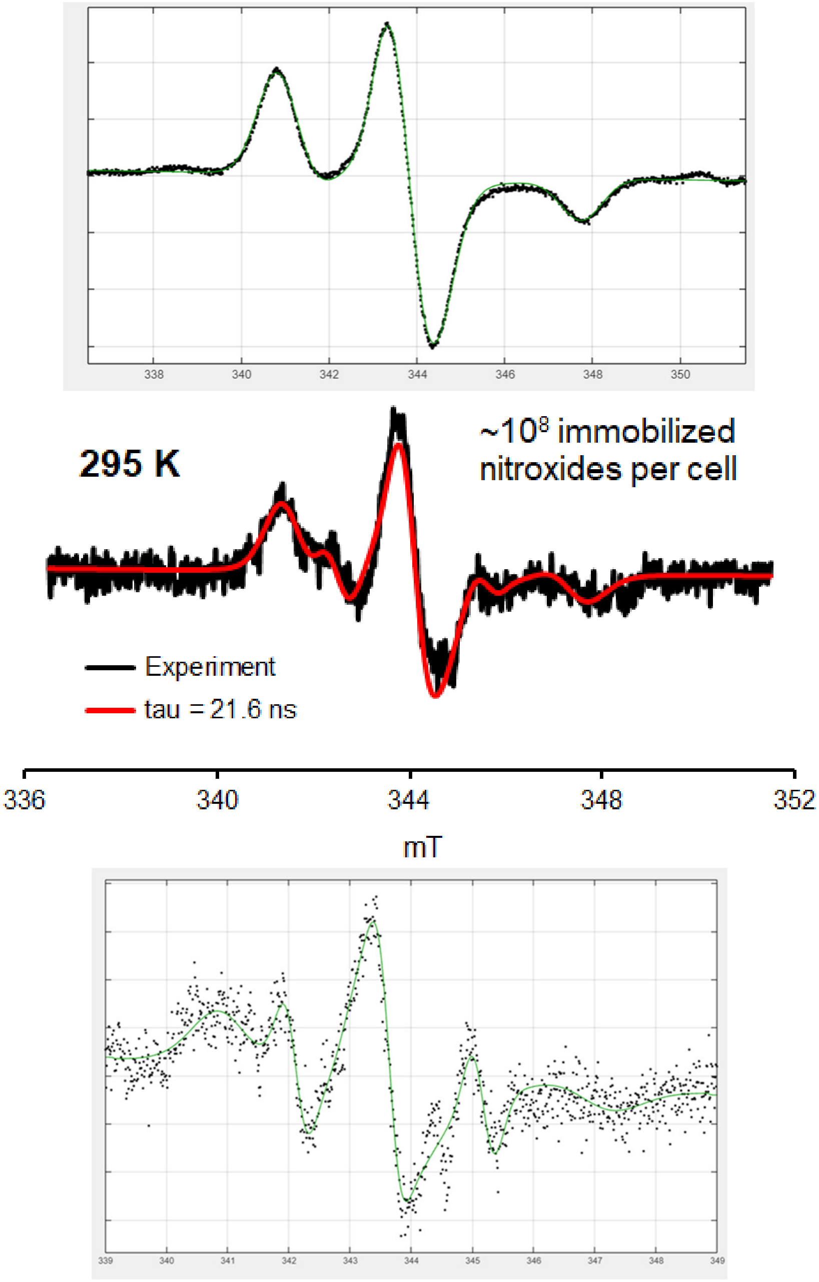

Fig. S4. EPR (X-band) spectra of agent **2a** in Jurkat cells. Same Jurkat cell experiment as in Fig. S3, showing a sample of washed (5x) cells resuspended in PBS buffer (label: JTP-8-48-A, Fig. S2). These cells are incubated with 0.49 mM **2a** for only 2 d and show approx. a 1:1 ratio of viable-to-dead cells. Converged simulations in the two top panels

are summarized in Table S1. Top panel: spectrum (black dots, label: JP849r30) at 243 K; spin count gives ca. 10^8 radicals per cell. Green line: simulation. Middle panel: spectrum (label: JP849r24) in a 5-mm OD EPR quartz tube, which is partially inserted into the cavity (Q = 1400); slow-to-fast component ratio = 1.00/0.03. Bottom panel: spectrum (black dots, label: JP849r14) recorded in a single 0.6 mm ID EPR quartz capillary (Q = 3300); simulation (green line) is not converged because of poor S/N and the presence of artifacts.

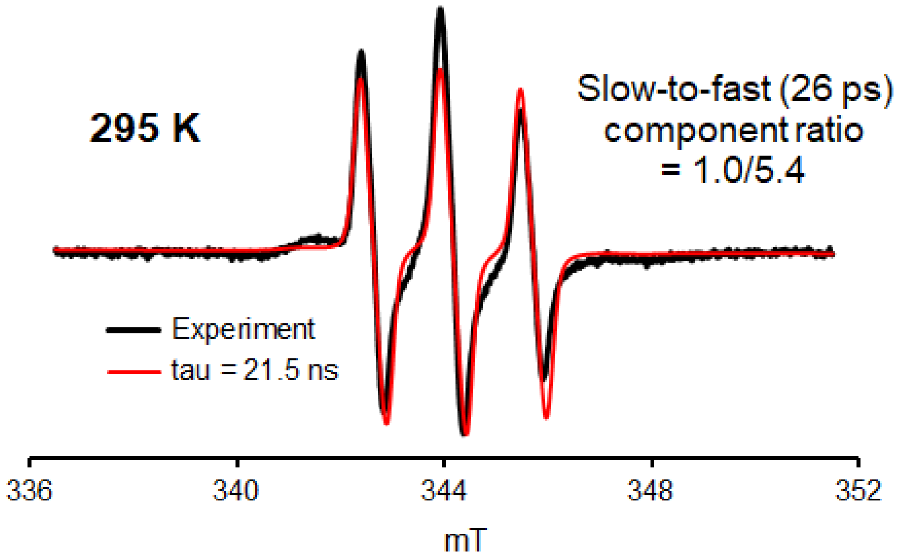

Fig. S5. Another Jurkat cell experiment with 24 h of incubation: EPR (X-band) spectrum (EPR label: JP845r17, sample label: JP-8-44-ASW) of agent **2a** in washed (5x) Jurkat cells resuspended in PBS buffer at 295 K showing two orders of magnitude smaller ratios of the slow-to-fast component, compared to 1.0/0.06 in Fig. S3B. The spectrum is recorded in a 3 mm OD EPR quartz tube partially inserted into cavity (Q-value = 2300). Simulation (rmsd = 0.01122) is carried out similarly to that in Fig. S3B and Table S1. In this experiment, nitroxide **2a** (sample label: JTP-7-74) in EtOH (0.15 mL) is added to 25 mL of media containing Jurkat cells (2.10×10^6 cells/mL), thus obtaining 0.56 mM nitroxide **2a** (based on quantitative EPR prior to incubation); the cell medium is incubated for only 24 h. After 24 h of incubation, the spin concentration of the medium (0.58 mM) is unchanged within the experimental error (<5%), and the density of cells is increased to 3.16×10^6 cells/mL. The control experiment (and background EPR spectra) was carried out by adding EtOH (0.15 mL) to 25 mL of media containing Jurkat cells (1.94×10^6 cells/mL) and then incubated for 24 h.

3. Summary of in vivo MRI.

Table S2. Summary of mice used for in vivo MRI.

mORCA	Run (mouse label)	Mouse 1 (M1)	Figures	Mouse 2 (M2)	Figures	Notes
**2**	SZ 1st		S37		S37	Heart T1 and T2 do not have day 1 pre
**2**	SZ 2nd		S8, S36		S35	Liver T2 M1 files damaged
**2**	SZ 3rd	dead	-	SZ497	S6, S7, S20, S34	For M1, a concentration of **2** was too high (120 mg/ml)
**2**	SZ 4th	SZ527	S10, S13, S33	SZ530	S11, S12, S21, S22, S32	M2 3rd i.v. failed; used i.p. instead
**1**	SD 1st	SD129	S16, S17, S29	SD130	S25, S26, S28	
**2**	SZ 5th	SZ538	S14, S15, S23, S24, S27, S31	SZ540	S9, S30	In this run, imaging was performed at t0, t0.5, and t72 only. M1 3rd i.v. failed; used i.p. instead

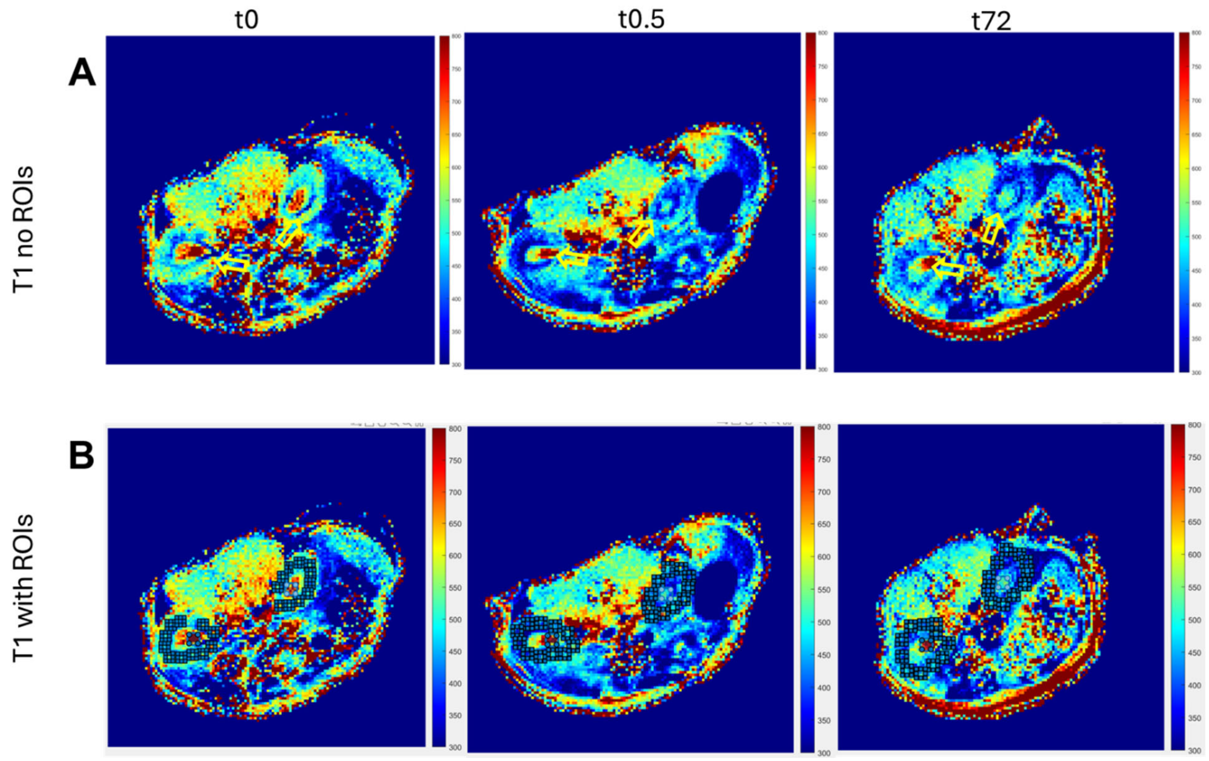

Fig. S6. Kidney MRI T_1 maps without ROIs (panel A) and T_1 maps with 11 square ROIs and 1 circular ROI (each kidney) (panel B) for slice 5 (mouse label: SZ_3rd_mouse2)

injected with mORCA **2** at 0 h (pre-injection), 0.5 h (post-injection), and 72 h (final image). Small, yellow arrows point to kidneys, and color-bar ranges are 300–800 ms, from blue to red. Panel A is identical to Figure 3 (panel A) in the main text.

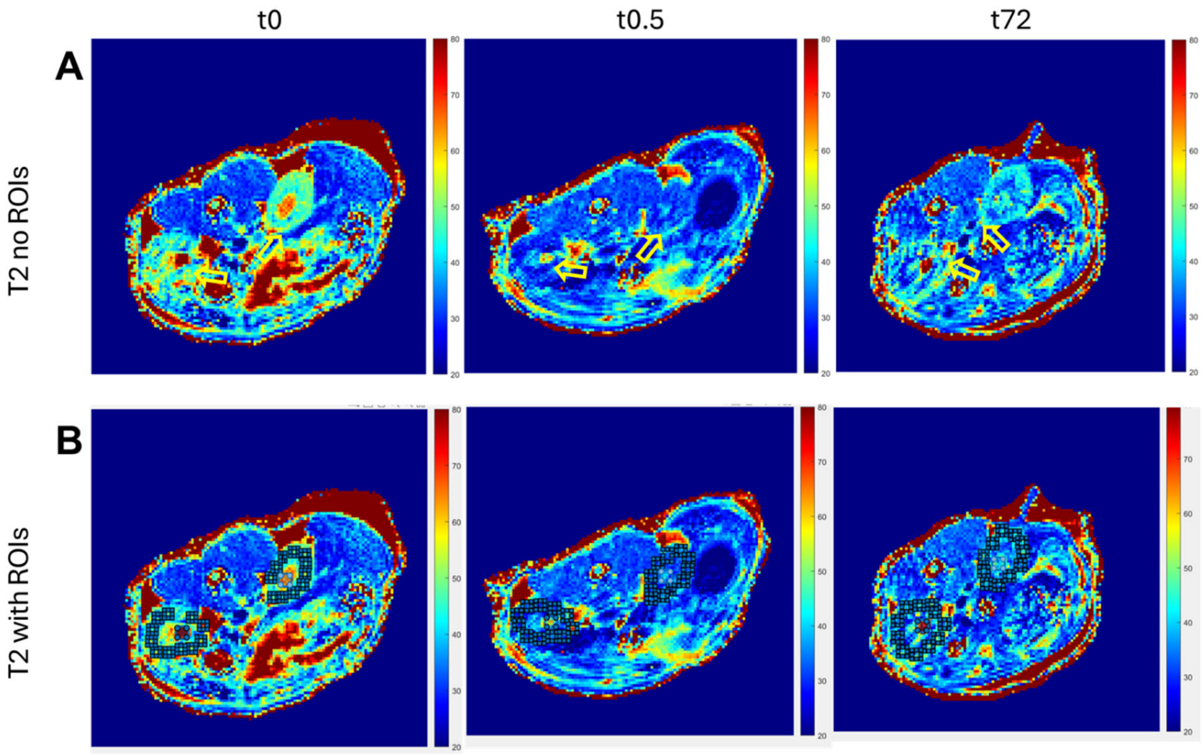

Fig. S7. Kidney MRI T_2 maps without ROIs of (panel A) and T_2 maps with 11 square ROIs and 1 circular ROI (each kidney) (panel B) for slice 5 (mouse label: SZ_3rd_mouse2) injected with mORCA **2** at 0 h (pre-injection), 0.5 h (post-injection), and 72 h (final image). Small, yellow arrows point to kidneys, and color-bar ranges are 20–80 ms, from blue to red. Panel A is identical to Figure 3 (panel B) in the main text.

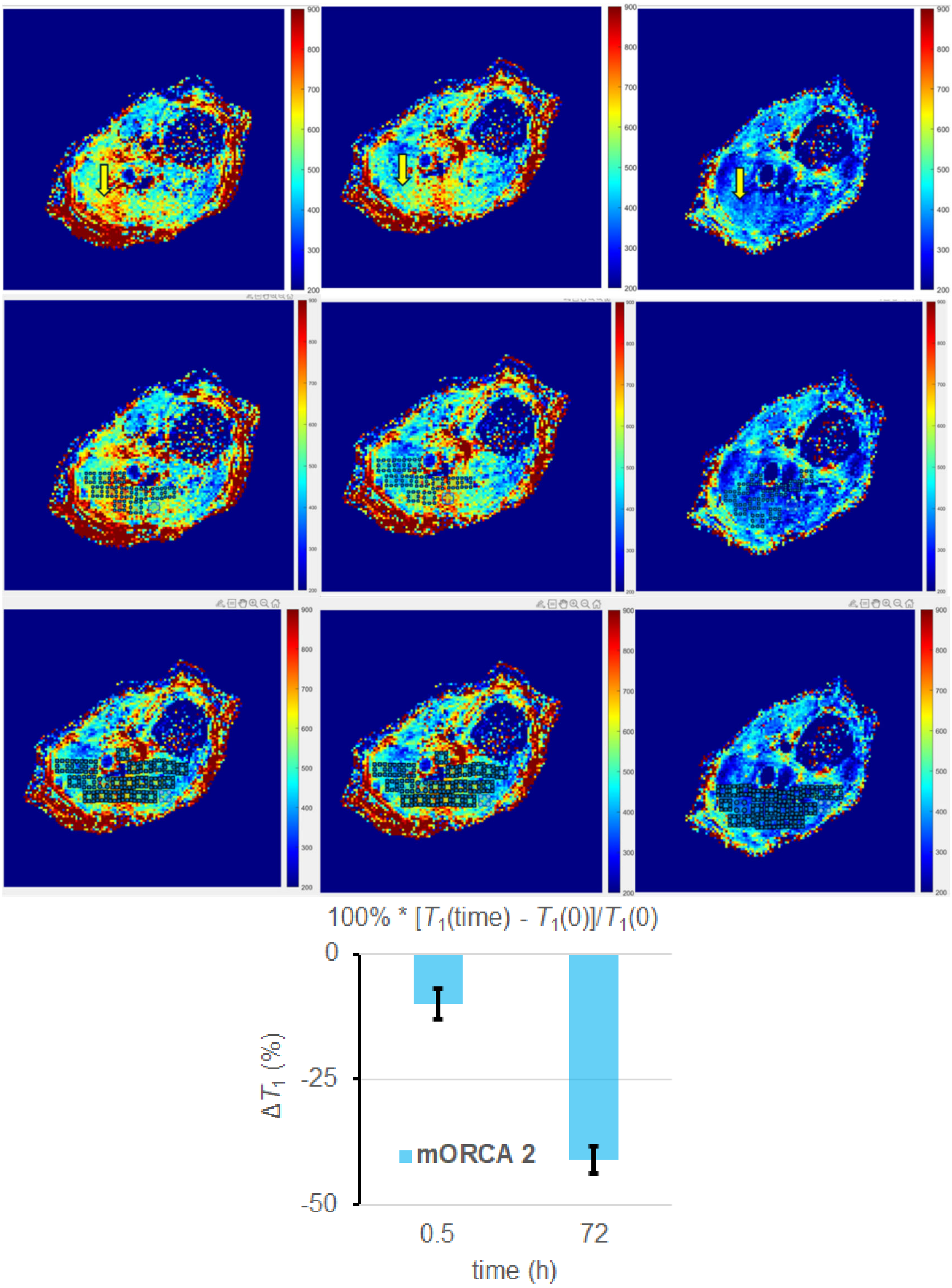

Fig. S8. Liver MRI T_1 maps for mORCA **2** with 11 square ROIs and 24 square ROIs for slice 3 (mouse label: SZ_2nd_mouse1). Color-bar ranges are 200–900 ms, from blue to red. <u>Bottom panel</u>: summary of T_1 relative vs. pre-injection at 0 h, post-injection at 0.5 h, and finally at 72 h; the decreases (mean ± SE) are –10 ± 3% (0.5 h) and –41± 3% (72 h) for

the entire mouse (all slices showing the liver, with the total number of ROIs in the 100–141 range), with SEs obtained through error propagation.

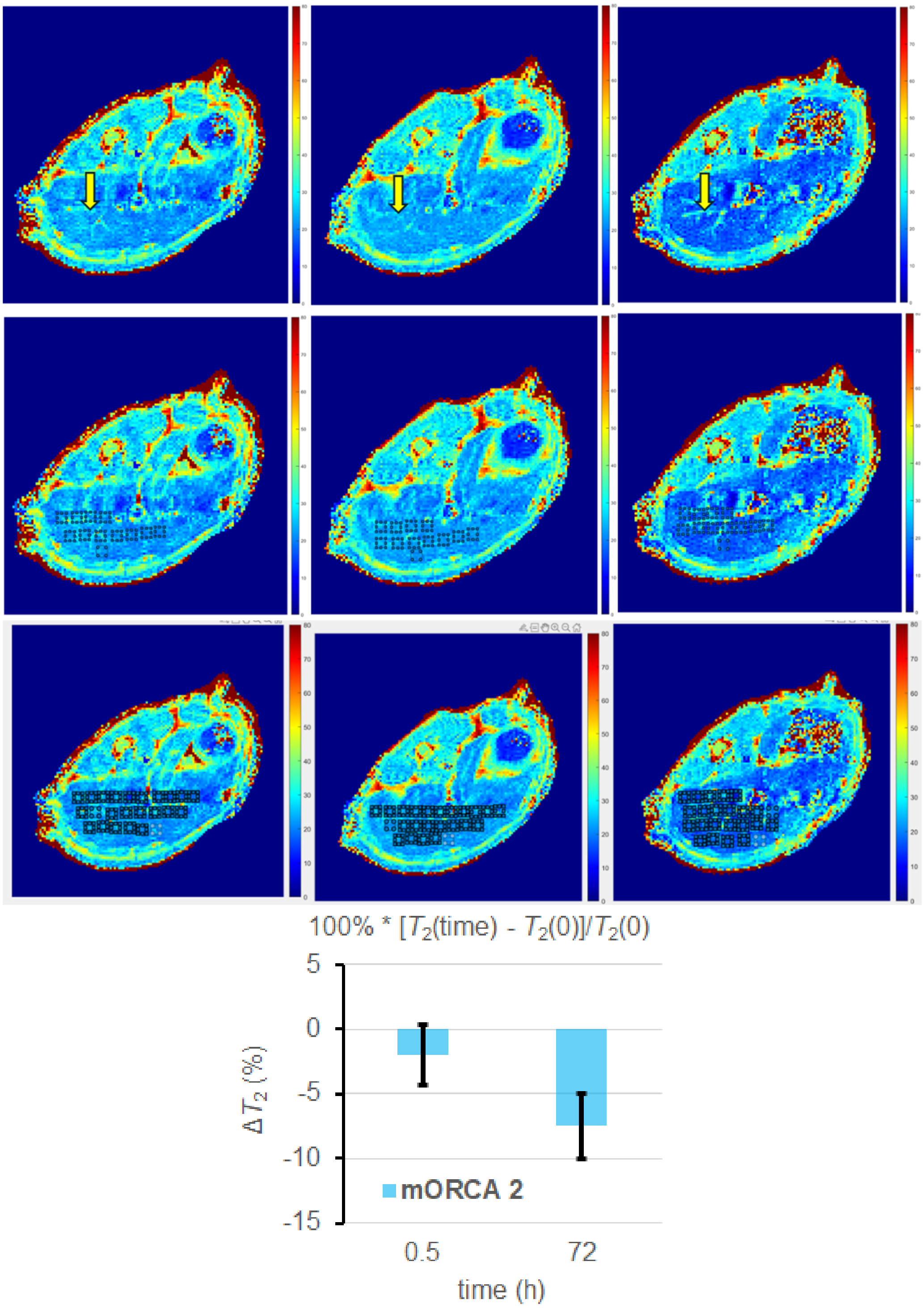

Fig. S9. Liver MRI T_2 maps for mORCA **2** with 11 square ROIs and 24 square ROIs for slice 8 (mouse label: SZ_5th_mouse2). Color-bar ranges are 0–80 ms, from blue to red.

Bottom panel: summary of T_2 relative vs. pre-injection post-injection at 0.5 h and finally at 72 h; the decreases (mean ± SE) are –2 ± 2% (0.5 h) and –7 ± 3% (72 h) for the entire mouse (all slices showing the liver, with the total number of ROIs in the 168–192 range), with SEs obtained through error propagation.

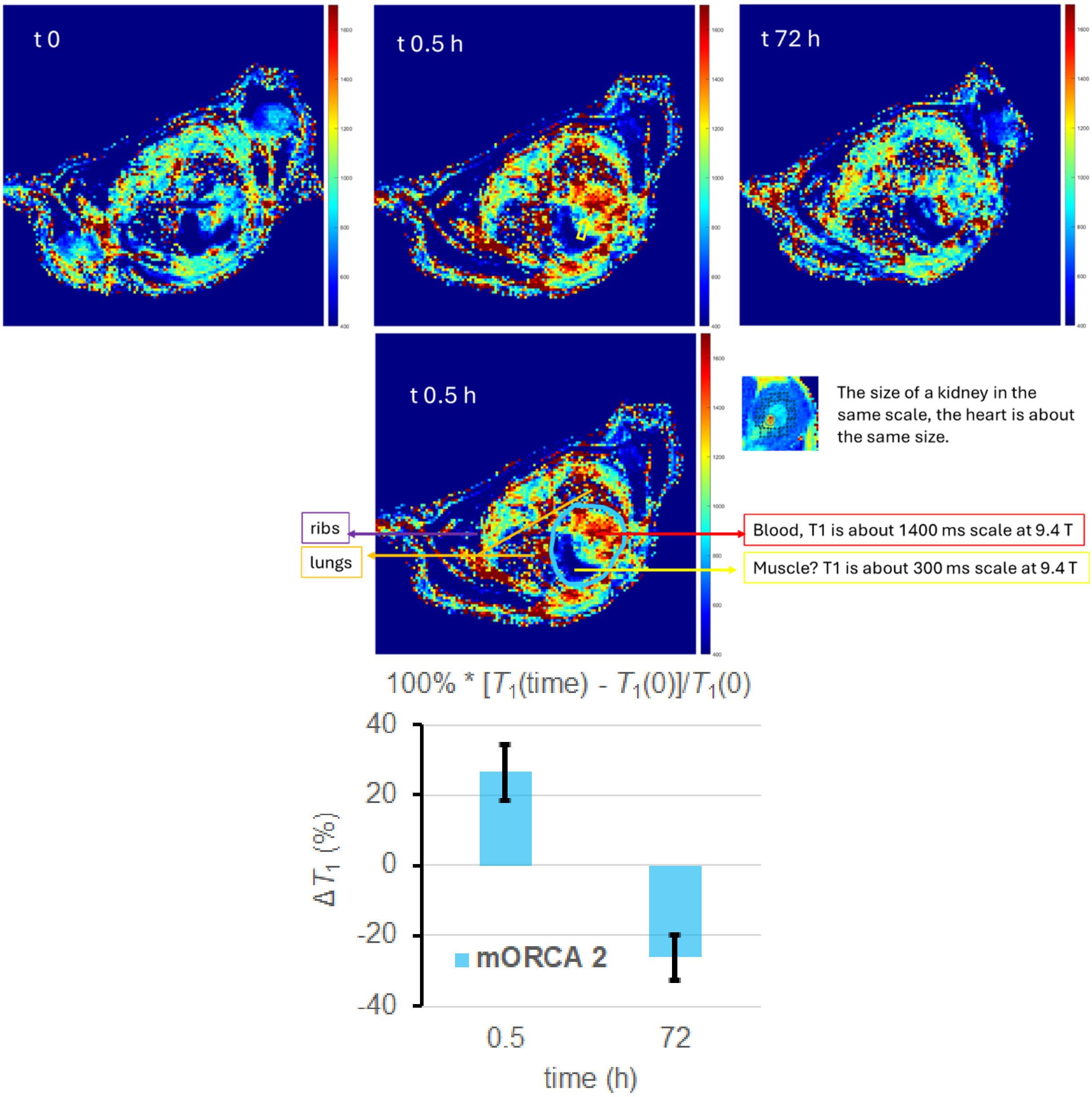

Fig. S10. Heart MRI T_1 maps for mORCA **2** for slice 5 (mouse label: SZ_4th_mouse1). Color-bar ranges are 400–1600 ms, from blue to red. Bottom panel: summary of T_1 relative vs. pre-injection post-injection at 0.5 h and finally at 72 h; the increase and decrease (mean ± SE) are +27 ± 8% (0.5 h) and -26 ± 7% (72 h) for the entire mouse (all slices showing the heart, with the total number of ROIs in the 71–79 range), with SEs obtained via error propagation.

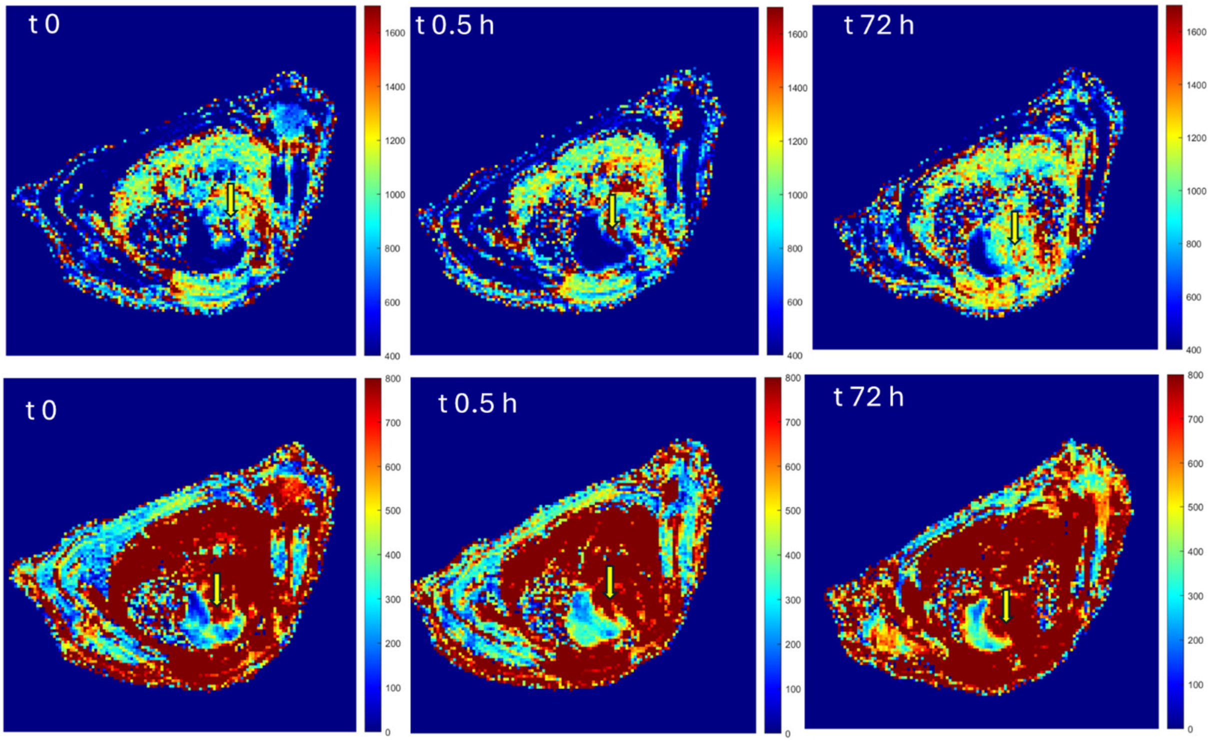

Fig. S11. Heart MRI T_1 maps for mORCA **2** for slice 5 (mouse label: SZ_4th_mouse2). Color-bar ranges are 400–1600 ms (top panel) and 0–800 ms (bottom panel), from blue to red.

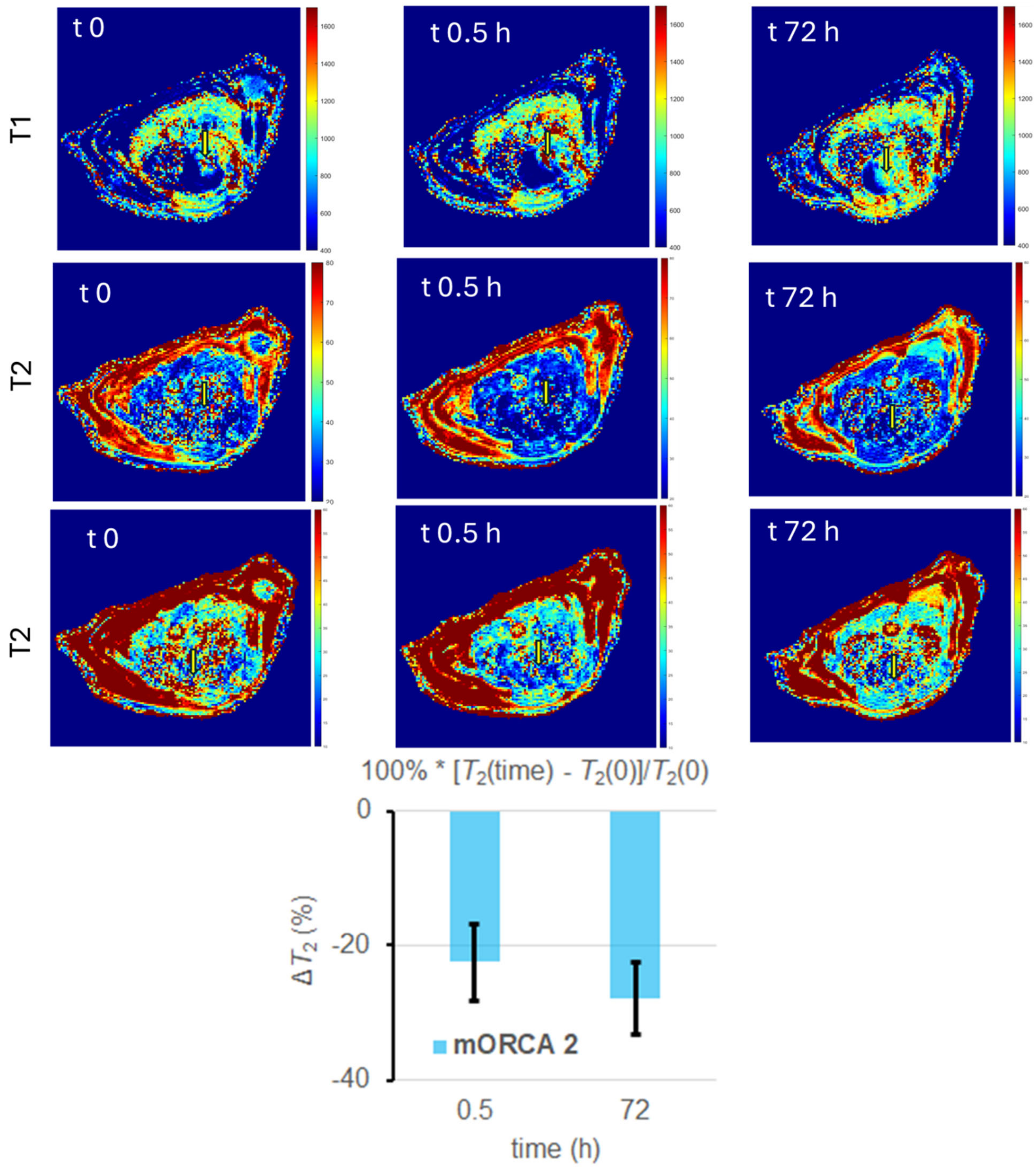

Fig. S12. Heart MRI T_1 (top panel) and T_2 maps (2nd and 3rd panels) for mORCA **2** for slice 5 (mouse label: SZ_4th_mouse2). Color-bar ranges are 400-1600 ms (top panel), 20-80 ms (middle panel), and 10-60 ms (bottom panel), from blue to red. Bottom panel: summary of T_2 relative vs. pre-injection at 0 h, post-injection at 0.5 h, and finally at 72 h; the decreases (mean ± SE) are -23 ± 6% (0.5 h) and -28 ± 5% (72 h) for the entire mouse (all slices showing the heart, with the total number of ROIs in the 79 – 80 range), with SEs obtained by error propagation.

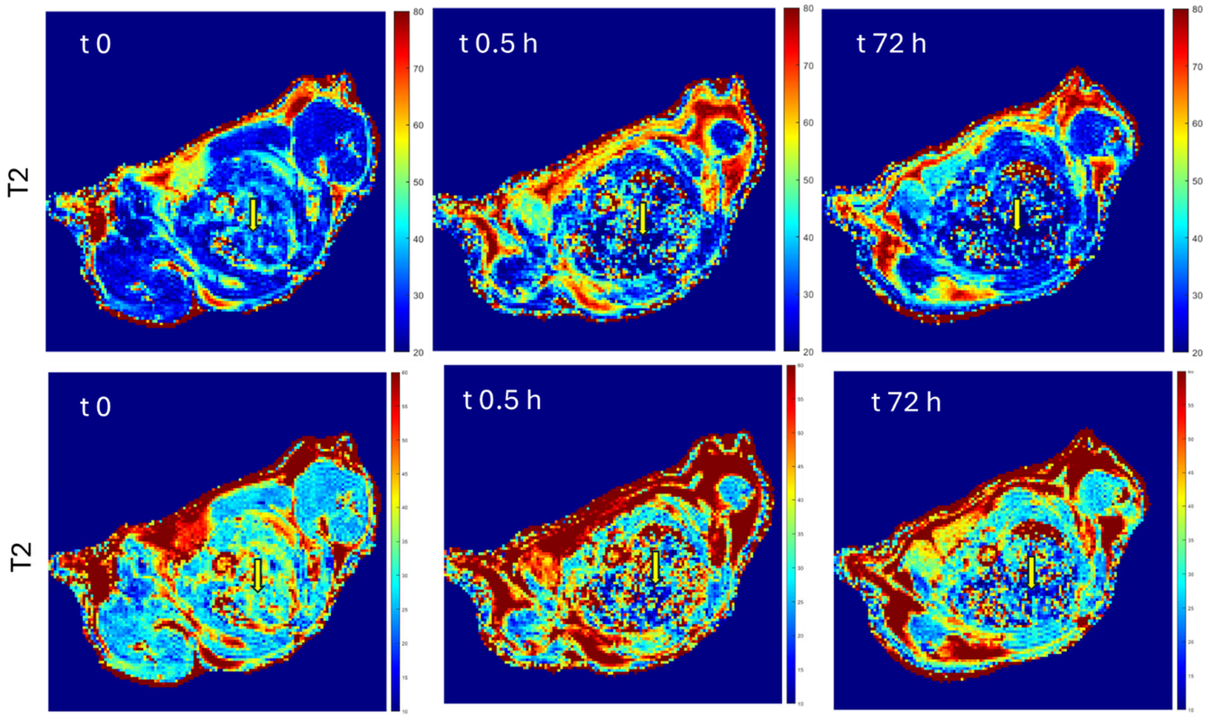

Fig. S13. Heart MRI T_2 maps for mORCA **2** for slice 3 (mouse label: SZ_4th_mouse1). Color-bar ranges are 20-80 ms (top panel) and 10-60 ms (middle panel), from blue to red.

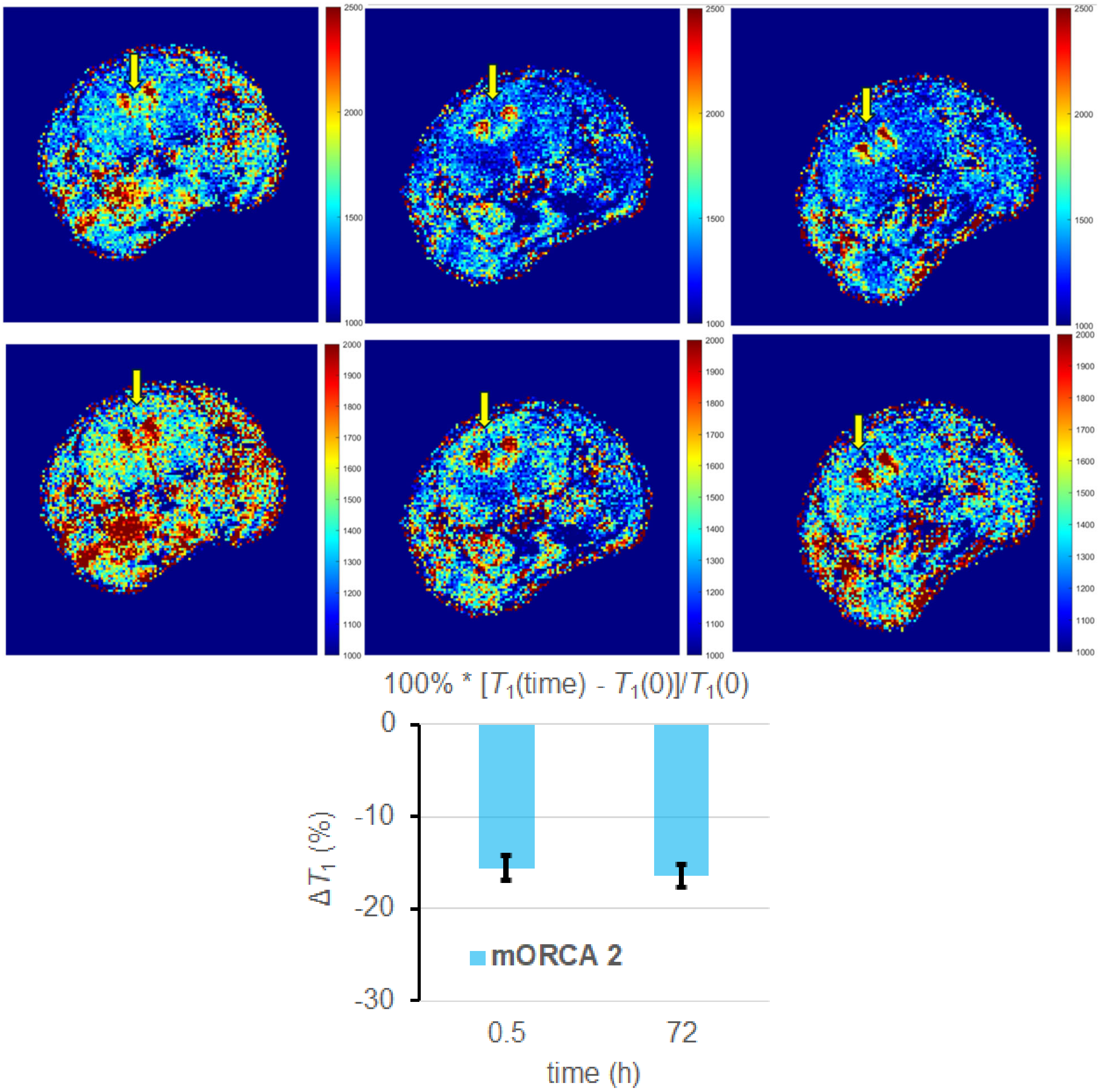

Fig. S14. Brain MRI T_1 maps for mORCA **2** for slice 4 (mouse label: SZ_5th_mouse1). Color-bar ranges are 1000–2500 ms, from blue to red. Bottom panel: summary of T_1 relative vs. pre-injection at 0 h, post-injection at 0.5 h, and finally at 72 h; the decreases (mean ± SE) are -16 ± 1% (0.5 h) and -16 ± 1% (72 h) for the entire mouse (all slices showing the brain, with the total number of ROIs equal to 168), with SEs obtained through error propagation.

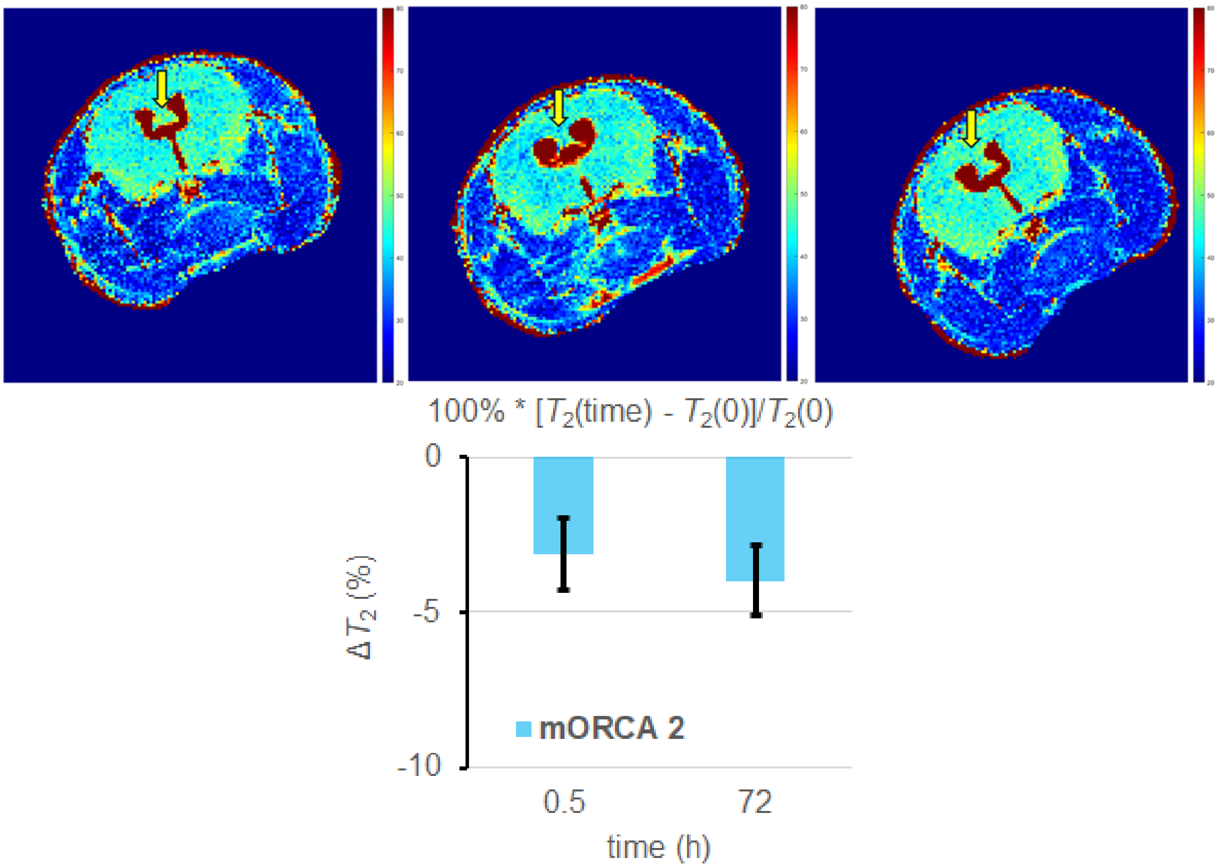

Fig. S15. Brain MRI T_2 maps for mORCA **2** for slice 4 (mouse label: SZ_5th_mouse1). Color-bar ranges are from 20 to 80 ms, from blue to red. Bottom panel: summary of T_2 relative vs. pre-injection at 0 h, post-injection at 0.5 h, and finally at 72 h; the decreases (mean ± SE) are -3 ± 1% (0.5 h) and -4 ± 1% (72 h) for the entire mouse (all slices showing the brain, with the total number of ROIs in the 129-152 range), with SEs obtained through error propagation.

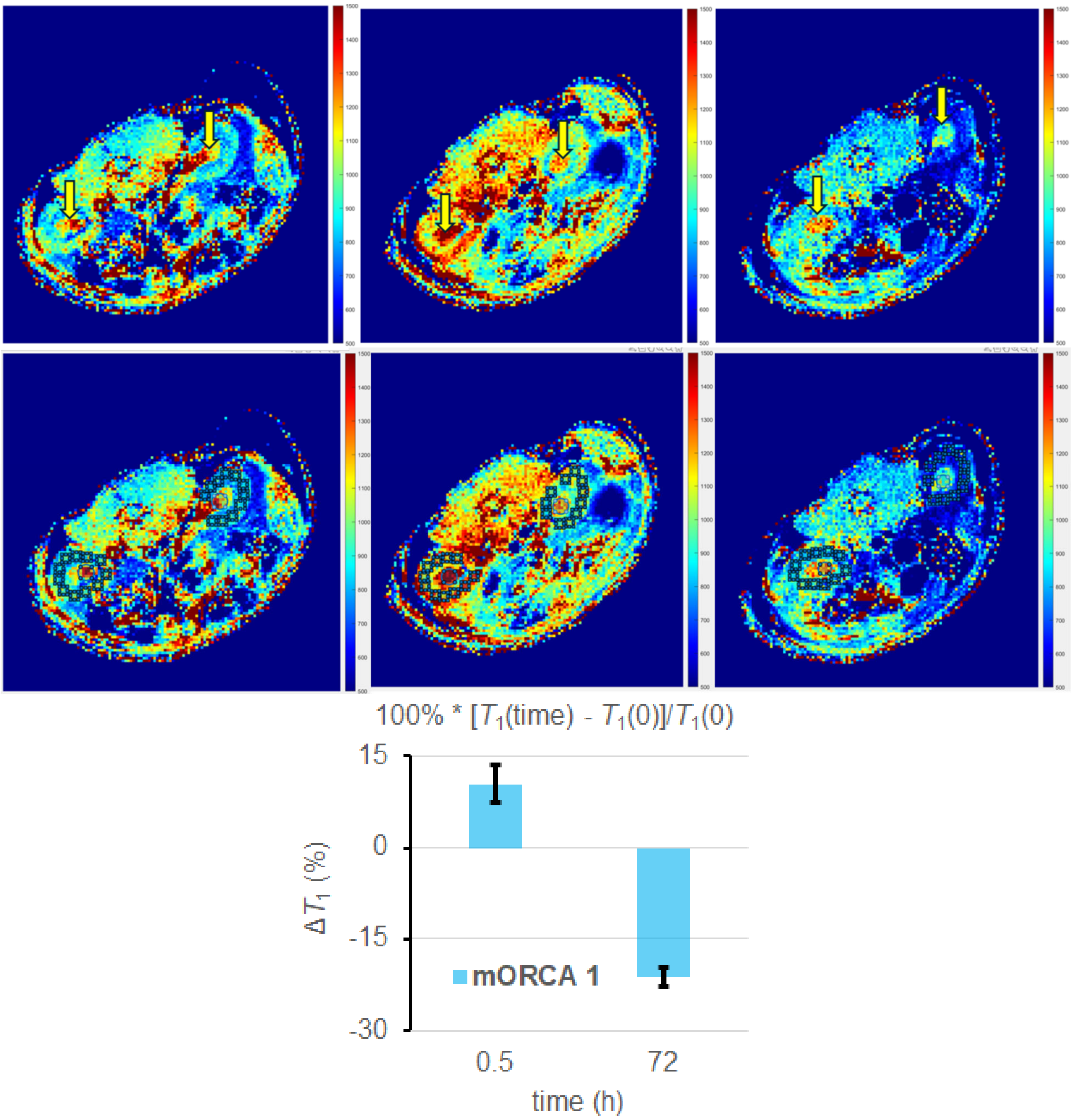

Fig. S16. Kidney MRI T_1 maps for mORCA **1** with 11 square ROIs and 1 circular ROI (each kidney) for slice 7 (mouse label: SD_1st_mouse1). Color-bar ranges are from 500 to 1500 ms, from blue to red. Bottom panel: summary of T_1 relative vs. pre-injection at 0 h, post-injection at 0.5 h, and finally at 72 h; the increase and decrease (mean ± SE) are +10 ± 3% (0.5 h) and -21 ± 2% (72 h) for the entire mouse (all slices showing the kidney, with the total number of ROIs in the 124-130 range), with SEs obtained through error propagation.

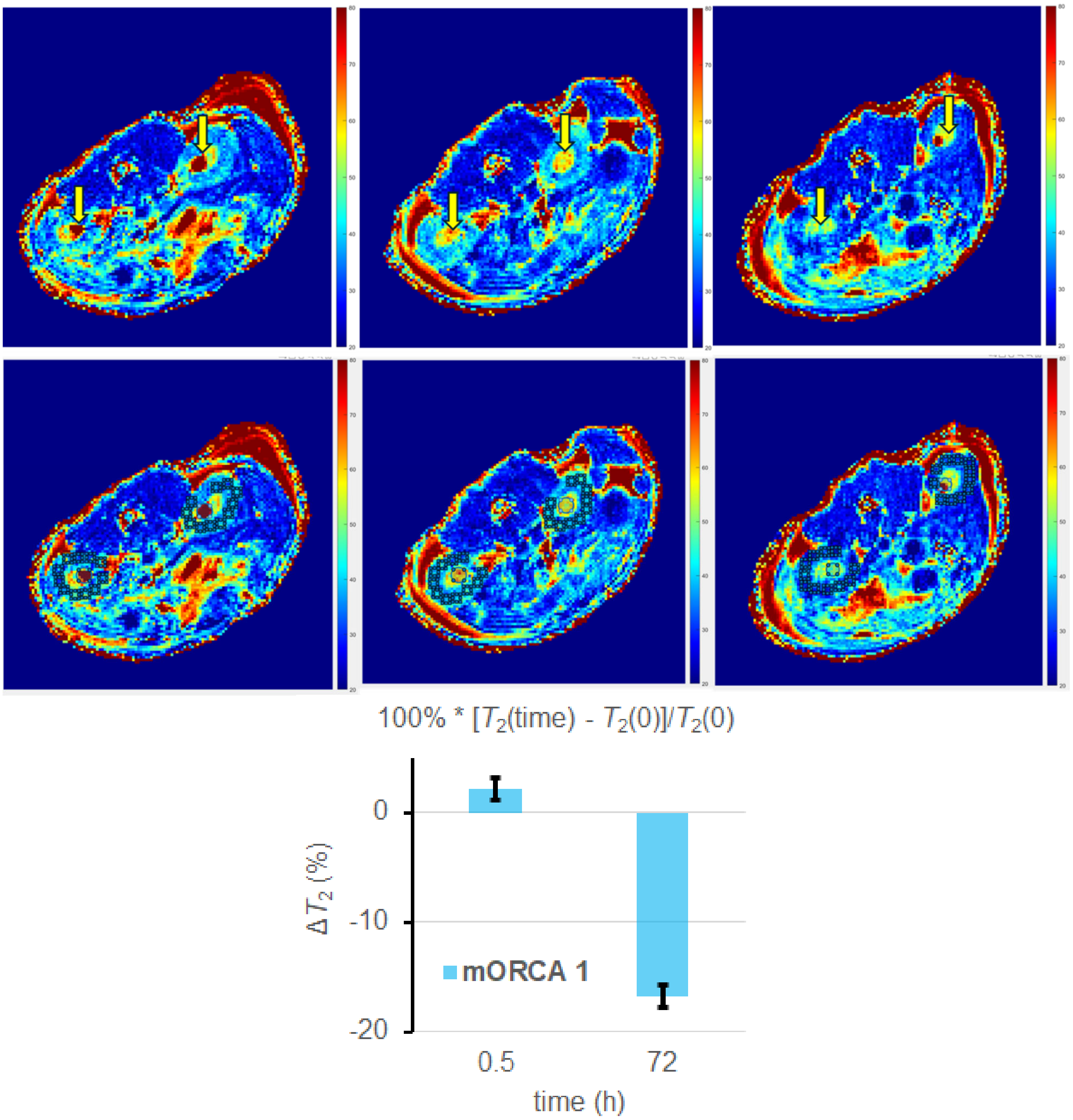

Fig. S17. Kidney MRI T_2 maps for mORCA **1** with 11 square ROIs and 1 circular ROI (each kidney) for slice 7 (mouse label: SD_1st_mouse1). Color-bar ranges are from 20 to 80 ms, from blue to red. Bottom panel: summary of T_2 relative vs. pre-injection at 0 h, post-injection at 0.5 h, and finally at 72 h; the increase and decrease (mean ± SE) are +2 ± 1% (0.5 h) and -17 ± 1% (72 h) for the entire mouse (all slices showing the kidney, with the total number of ROIs in the 147–171 range), with SEs obtained via error propagation.

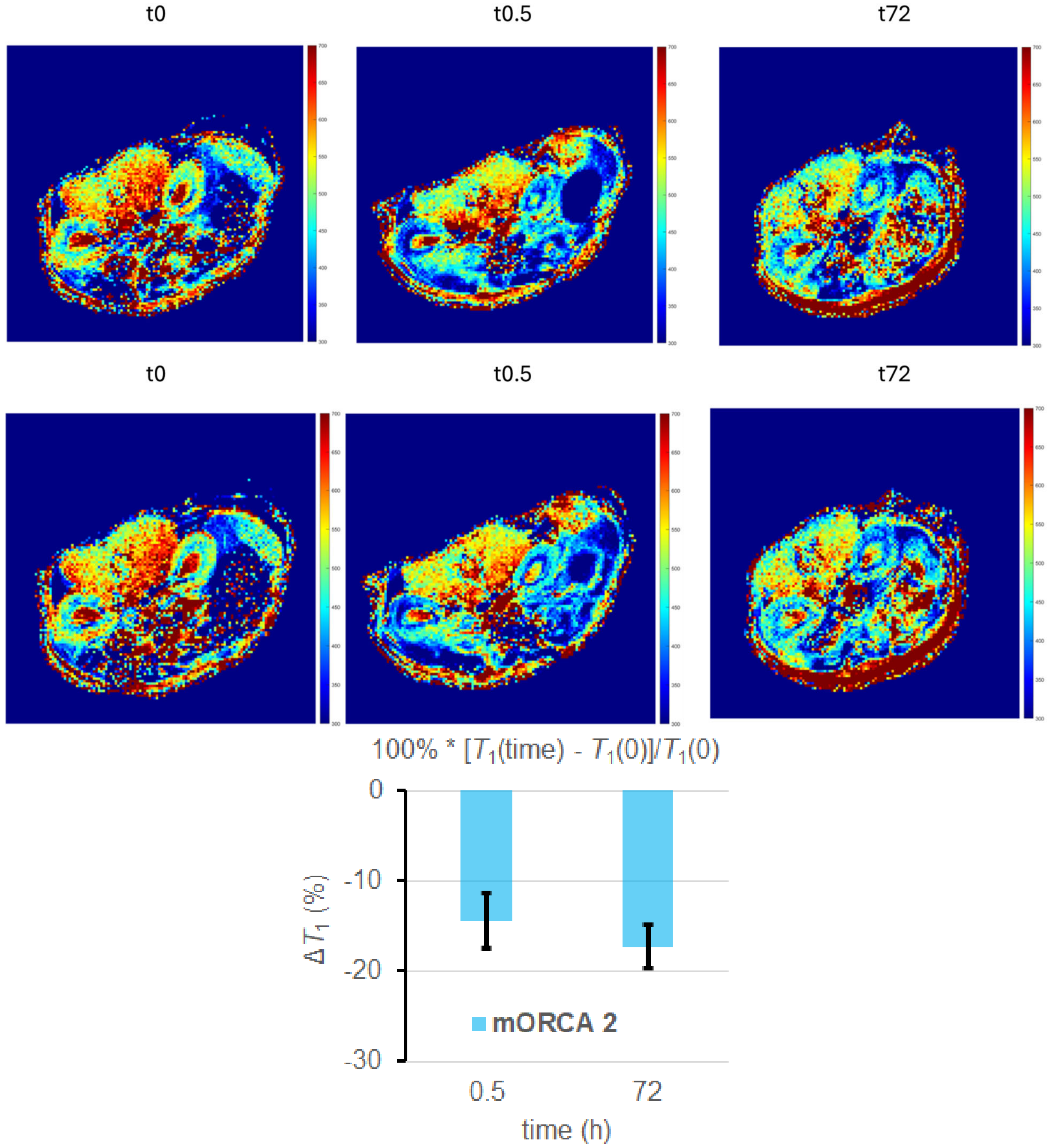

Fig. S18. Modified figure showing two slices at a different color-bar range, from blue to red, that is, 300–700 ms, compared to 300–800 ms in the main text (Figure 3). Kidney MRI T_1 maps for mORCA **2** showing slices 5 (top panel) and 6 (middle panel); mouse label: SZ_3rd_M2. Bottom panel: summary of T_1 relative vs. pre-injection at 0 h, post-injection at 0.5 h, and finally at 72 h; the decreases (mean ± SE) are $\Delta T_1 = -14 \pm 3\%$ (0.5 h) and $\Delta T_1 = -17 \pm 2\%$ (72 h) for the entire mouse (all slices showing the kidneys, with the total number of ROIs in the 109–154 range), with SEs obtained via error propagation.

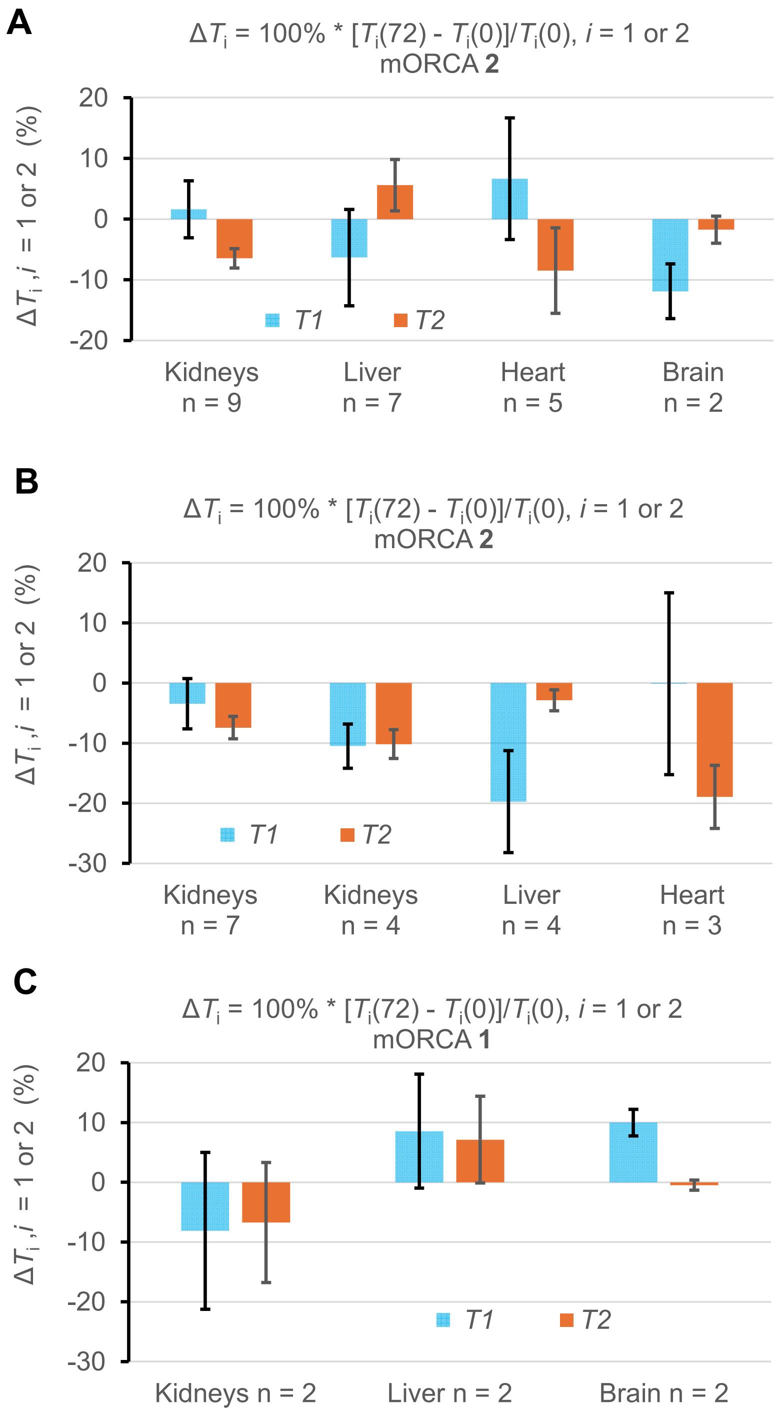

Fig. S19. Modified figure showing additional panel C for mORCA **1**, compared to that in the main text (Figure 4). Summary of relative ΔT_1 and ΔT_2 (%) for T_1 and T_2 maps at 72 h vs. 0 h (pre-injection) for mice injected with the mORCA. Panel A: maps acquired for all studied animals using mORCA **2**. Panel B: maps for selected animals (n = 3–7) using mORCA **2**. Panel C: maps for all animals (n = 2) using mORCA **1**.

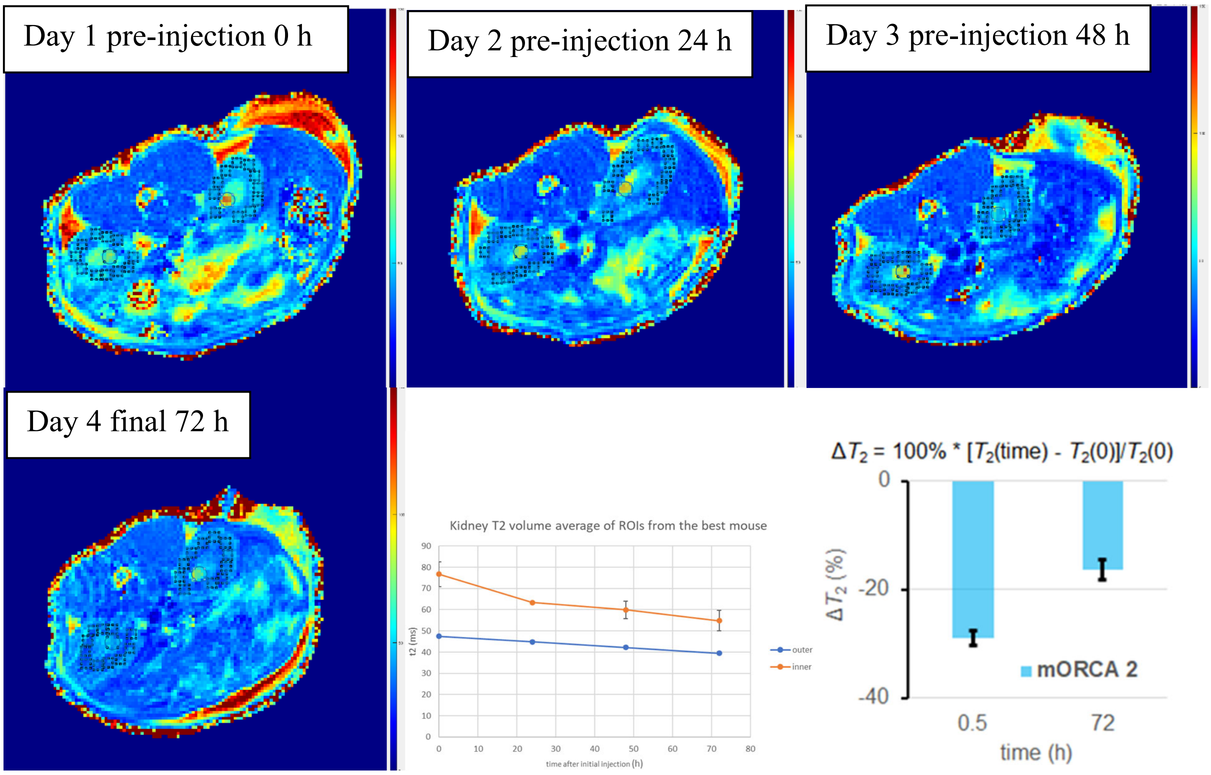

Fig. S20. Kidney MRI T_2 maps for mORCA **2** with 22 squares for the outer part of the kidneys and 2 circles for the inner part of the kidneys for slice 6 (mouse label: SZ_3rd_mouse2 – same mouse), as shown in Figure 3 (main text). Color-bar ranges are from 0 to 150 ms, from blue to red. Bottom middle panel: summary of T_2 (ms) changes (mean ± SE) for the maps shown above for both outer and inner parts of the kidneys (few slices showing the kidneys). Bottom right panel: summary of T_2 relative vs. pre-injection at 0 h, post-injection at 0.5 h (map not shown), and finally at 72 h (only outer part of kidneys); the decreases (mean ± SE) are –23 ± 6% and –28 ± 5% for the entire mouse (few slices showing the kidney, with the total number of ROIs in the 132–154 range for the outer part of the kidneys), with SEs obtained through error propagation.

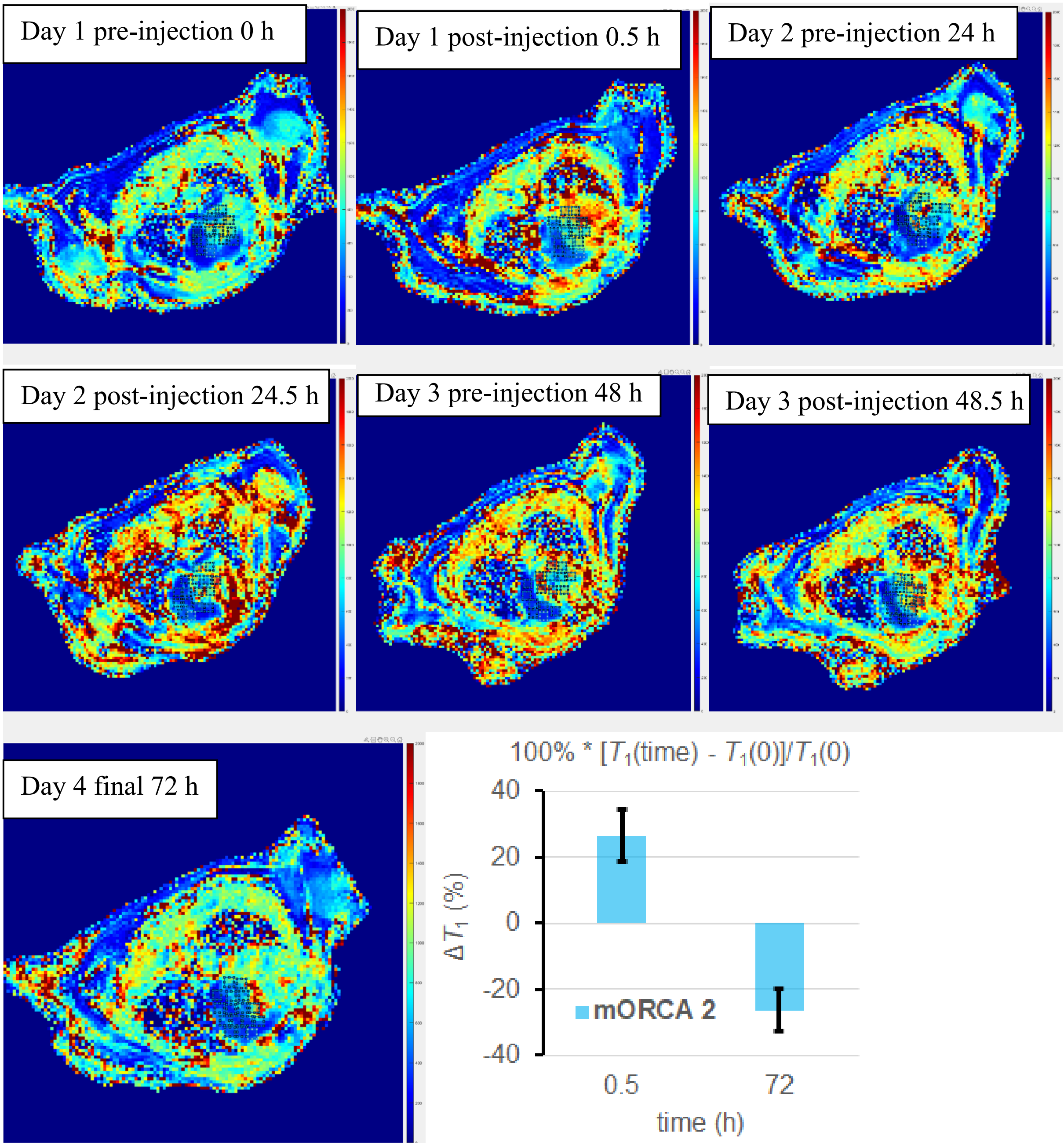

Fig. S21. Heart MRI T_1 maps for mORCA **2** with 16 square ROIs for slice 5 (mouse label: SZ_4th_mouse2). Color-bar ranges are 0–2000 ms, from blue to red. Bottom right panel: summary of T_1 relative vs. pre-injection at 0 h, post-injection at 0.5 h, and finally at 72 h; the increase and decrease (mean ± SE) are +27 ± 8% (0.5 h) and –26 ± 7% (72 h) for the entire mouse (all slices showing the heart, with the total number of ROIs in the 71–79 range), with SEs obtained via error propagation.

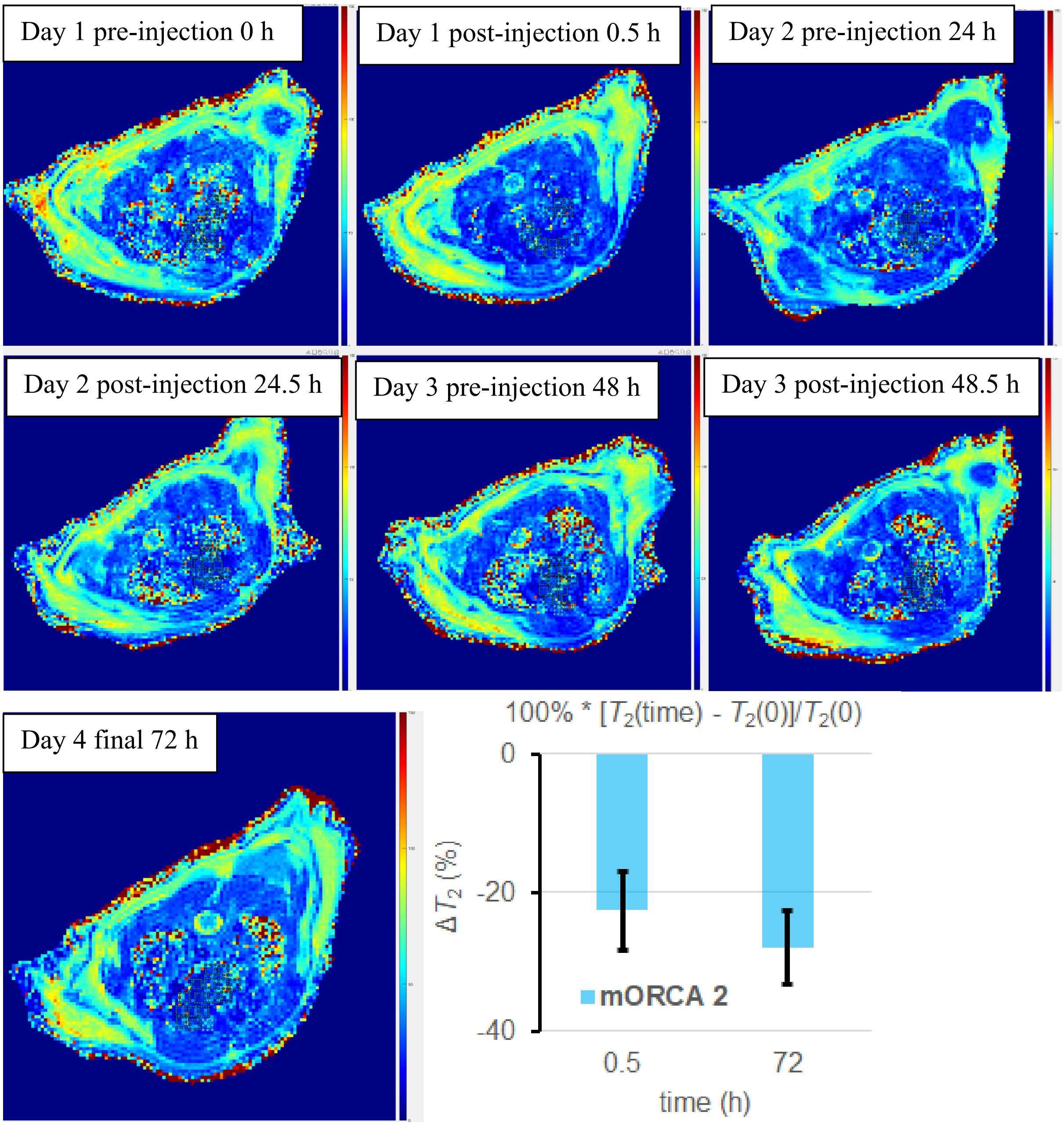

Fig. S22. Heart MRI T_2 maps for mORCA **2** with 16 square ROIs for slice 5 (mouse label: SZ_4th_mouse2). Color-bar ranges are from 0 to 150 ms, from blue to red. Bottom right panel: summary of T_2 relative vs. pre-injection at 0 h, post-injection at 0.5 h, and finally at 72 h; the decreases (mean ± SE) are –23 ± 6% and –28 ± 5% for the entire mouse (few slices showing the heart, with the total number of ROIs in the 79–80 range), with SEs obtained via error propagation.

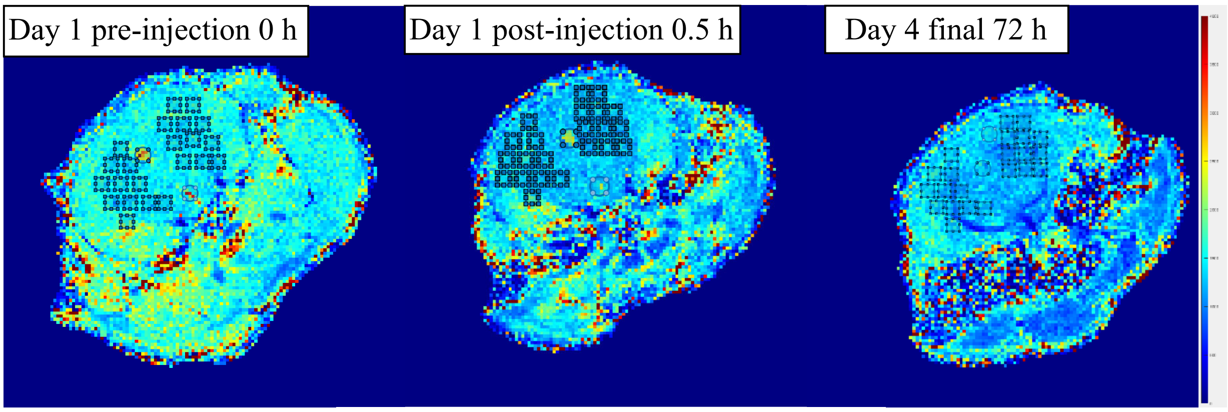

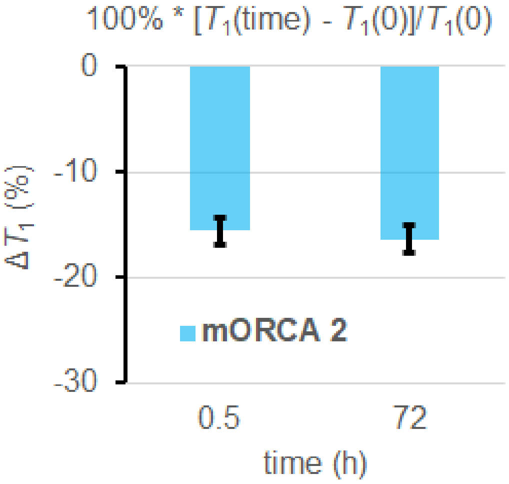

Fig. S23. Brain MRI T_1 maps for mORCA **2** with 22 square ROIs and 2 circular ROIs for slice 6 (mouse label: SZ_5th_mouse1). Color-bar ranges are 0–4000 ms, from blue to red. Bottom panel: summary of T_1 relative vs. pre-injection at 0 h, post-injection at 0.5 h, and finally at 72 h; the decreases (mean ± SE) are $\Delta T_1 = -16 \pm 1\%$ (0.5 h) and $\Delta T_1 = -16 \pm 1\%$ (72 h) for the entire mouse (all slices showing the brain, with the total number of ROIs equal to 168), with SEs obtained through error propagation.

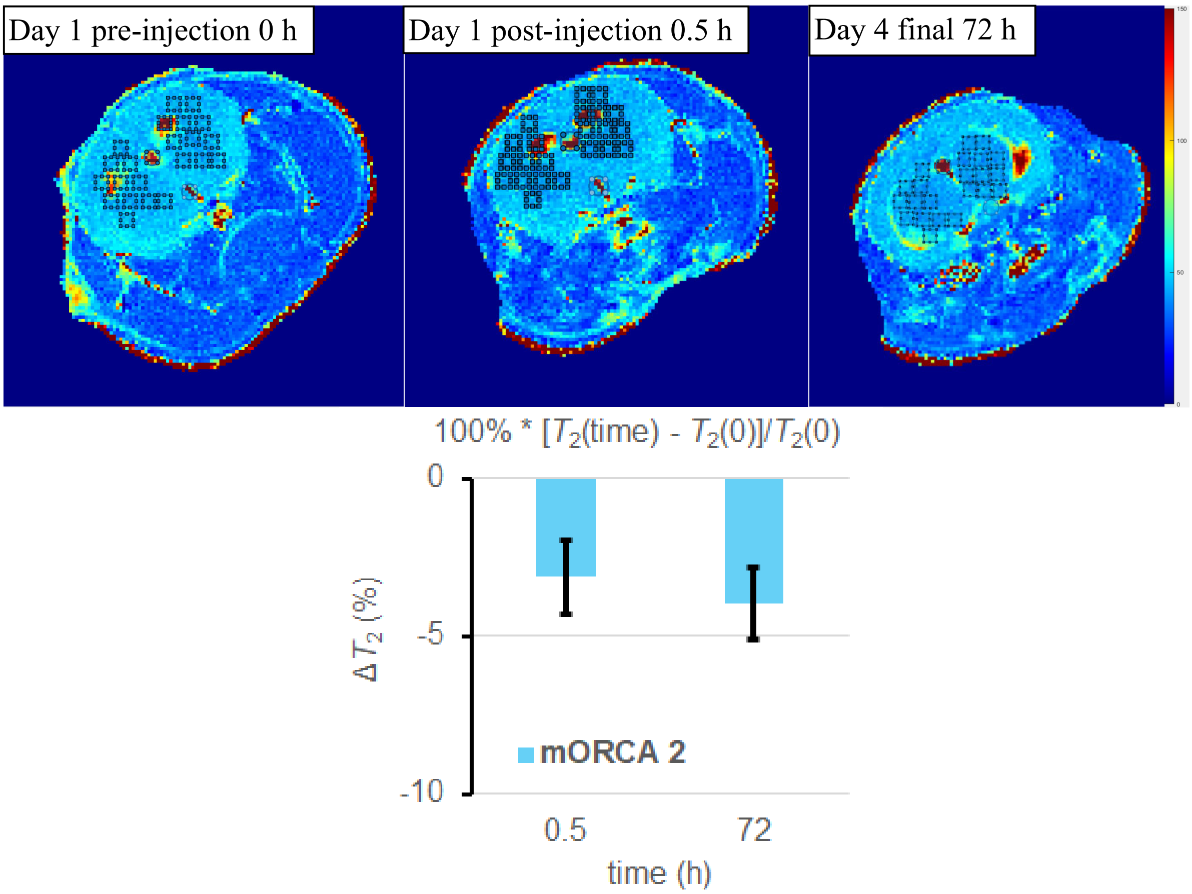

Fig. S24. Brain MRI T_2 maps for mORCA **2** with 22 square ROIs and 2 circular ROIs for slice 5 (mouse label: SZ_5th_mouse1). Color-bar ranges are from 0 to 150 ms, from blue to red. Bottom panel: summary of T_2 relative vs. pre-injection at 0 h, post-injection at 0.5 h, finally at 72 h; the decreases (mean ± SE) are $\Delta T_2 = -3.1 \pm 1.2\%$ (0.5 h) and $\Delta T_2 = -4.0 \pm 1.1\%$ (72 h) for the entire mouse (all slices showing the brain, with the total number of ROIs in the 129–152 range), with SEs obtained via error propagation.

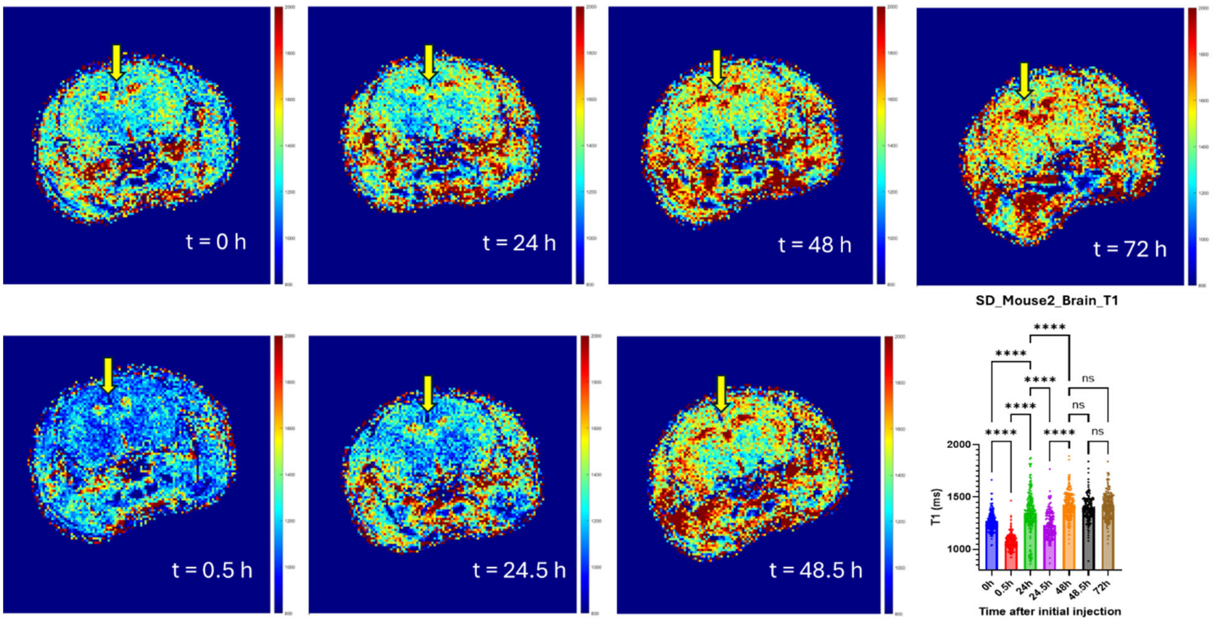

Fig. S25. Brain MRI T_1 maps for mORCA **1** (mouse label: SD_Mouse2_Brain). Color-bar ranges are from 800 to 2000 ms, from blue to red. Bottom right panel: T_1 relaxation times before and after injections based on in vivo MRI imaging for the brain (mouse label: SD_mouse2). Three injections were administered at 0 h, 24 h, and 48 h, with MRI measurements taken before and after each injection, followed by a final measurement at 72 h. The bars represent the volume-averaged T_1 for the brain. Each dot within a bar corresponds to the average T_1 value of a region of interest (ROI, a voxel in the MRI image). Absolute T_1 values were extracted directly from MRI T_1 maps using a self-developed MATLAB code. Statistical significance was accessed using ordinary one-way ANOVA to compare means across time points, with significance levels denoted (*, $P < 0.05$; **, $P < 0.01$; ***, $P < 0.001$; and ***, $P < 0.0001$). All statistical analyses and graphical representations were performed using GraphPad Prism 10.

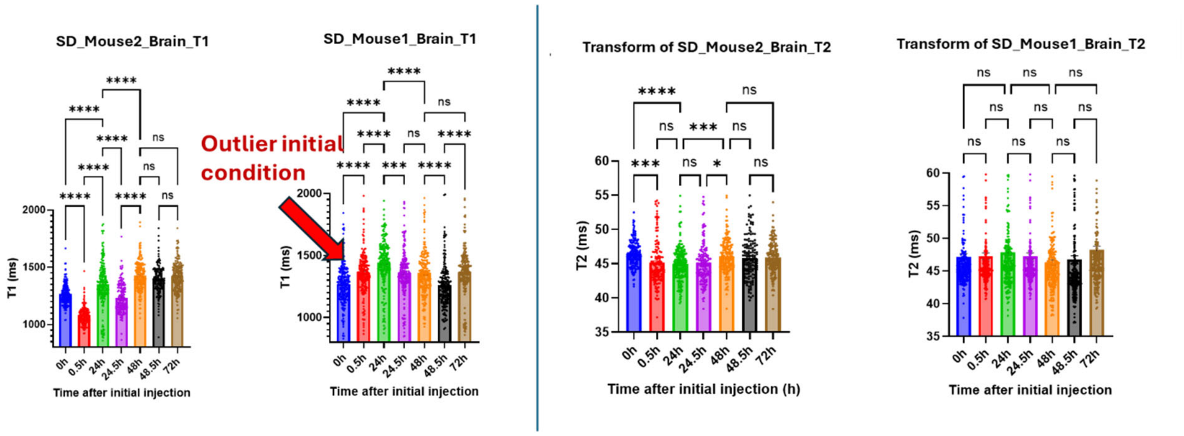

Fig. S26. T_1 and T_2 relaxation times before and after injections of mORCA **1** based on in vivo MRI imaging for the brain (mouse label: SD_mouse2). Three injections were administered at 0 h, 24 h, and 48 h, with MRI measurements taken before and after each injection, followed by a final measurement at 72 h. The bars represent the volume-averaged T_1 and T_2 values for the brain. Each dot within a bar corresponds to the average T_1 or T_2 value of a region of interest (ROI, a voxel in the MRI image). Absolute T_1 and T_2 values were extracted directly from MRI T_1 and T_2 maps using a self-developed MATLAB code. Statistical significance was accessed using ordinary one-way ANOVA to compare means across time points, with significance levels denoted (*, P < 0.05; **, P < 0.01; ***, P < 0.001; and ***, P < 0.0001). All statistical analyses and graphical representations were performed using GraphPad Prism 10.

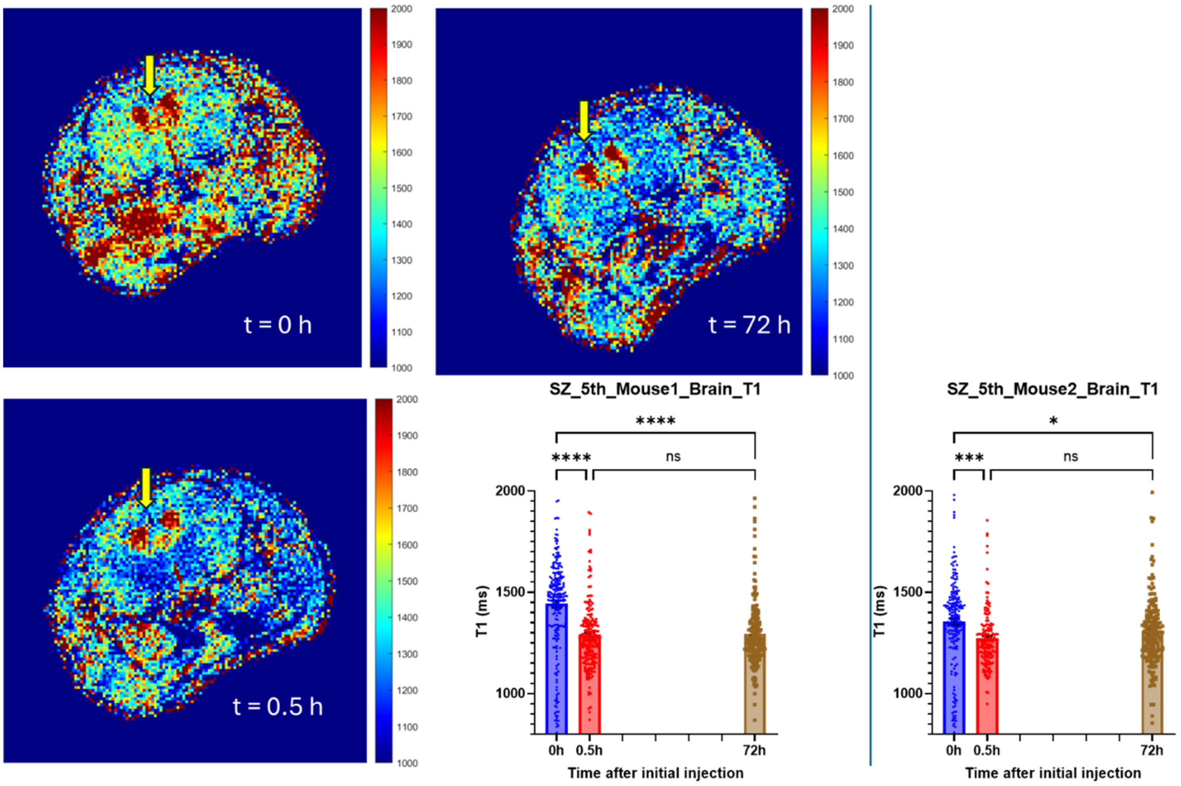

Fig. S27. Brain MRI T_1 maps for mORCA **2** (mouse label: SZ_5th_Mouse1). Color-bar ranges are from 1000 to 2000 ms, from blue to red. Bottom middle and right panel: T_1 relaxation times for pre-injection at 0 h, post-injection at 0.5 h, and finally at 72 h based on in vivo MRI imaging for the brain (mouse label: SZ_5th_Mouse1 and SZ_5th_Mouse2). The bars represent the volume-averaged T_1 for the brain. Each dot within a bar corresponds to the average T_1 value of a region of interest (ROI, a voxel in the MRI image). Absolute T_1 values were extracted directly from MRI T_1 maps using a self-developed MATLAB code. Statistical significance was accessed using ordinary one-way ANOVA to compare means across time points, with significance levels denoted (*, $P < 0.05$; **, $P < 0.01$; ***, $P < 0.001$; and ***, $P < 0.0001$). All statistical analyses and graphical representations were performed using GraphPad Prism 10.

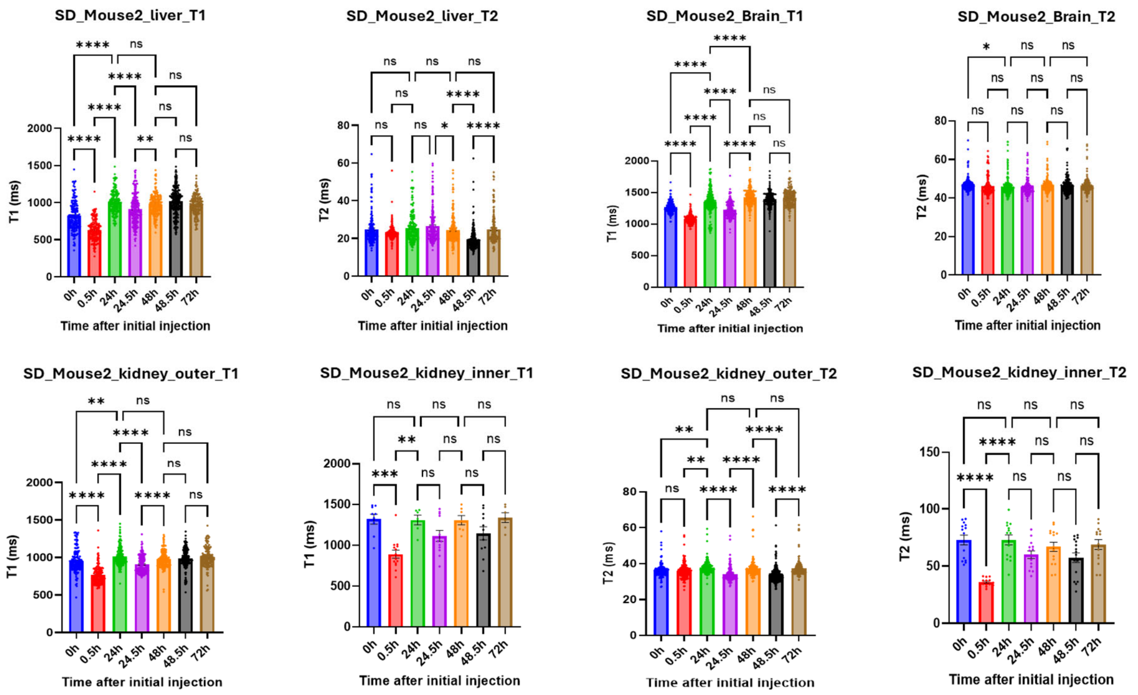

Fig. S28. T_1 and T_2 relaxation times before and after injections of mORCA **1** based on in vivo MRI imaging for the liver, brain, and kidney (mouse label: SD_mouse2). Three injections were administered at 0 h, 24 h, and 48 h, with MRI measurements taken before and after each injection, followed by a final measurement at 72 h. The bars represent the volume-averaged T_1 and T_2 values for each organ. Each dot within a bar corresponds to the average T_1 or T_2 value of a region of interest (ROI, a voxel in the MRI image). For each organ at each time point, data were collected from 5 to 8 transverse plane MRI slices, with 12–24 ROIs analyzed per slice, resulting in a total of 60–192 ROIs per organ. Absolute T_1 and T_2 values were extracted directly from MRI T_1 and T_2 maps using a self-developed MATLAB code. Statistical significance was accessed using ordinary one-way ANOVA to compare means across time points, with significance levels denoted (*, $P < 0.05$; **, $P < 0.01$; ***, $P < 0.001$; and ***, $P < 0.0001$). All statistical analyses and graphical representations were performed using GraphPad Prism 10.

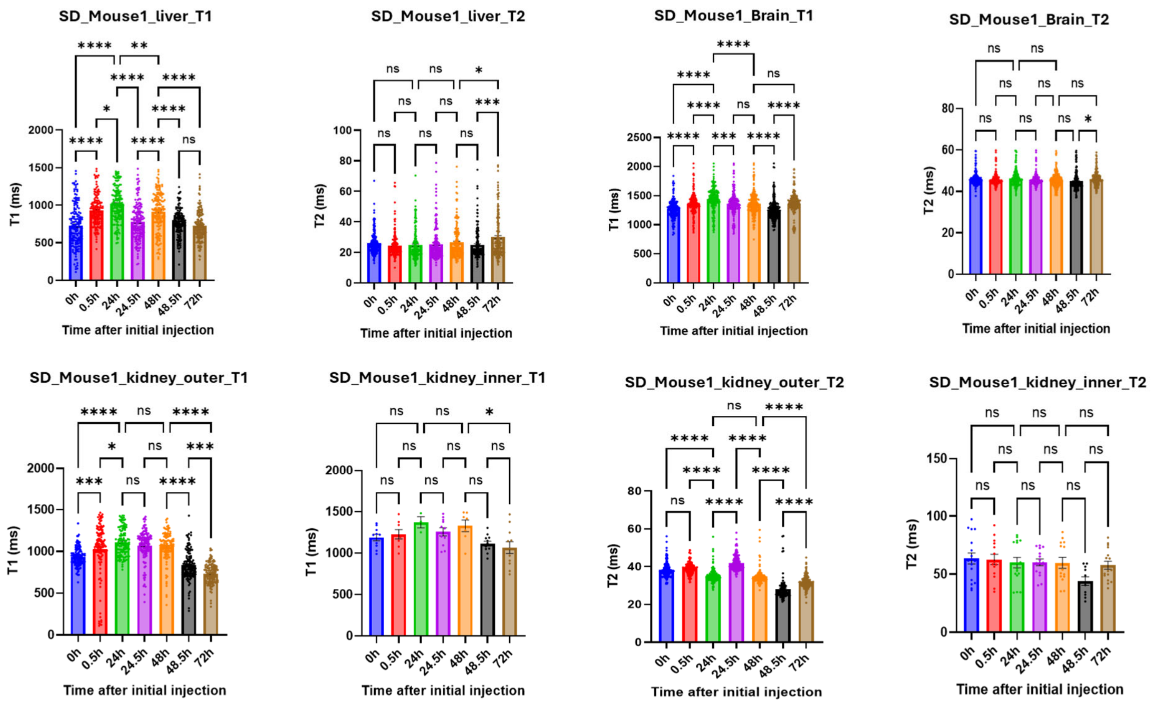

Fig. S29. T_1 and T_2 relaxation times before and after injections of mORCA **1** based on in vivo MRI imaging for the liver, brain, and kidney (mouse label: SD_mouse1). Three injections were administered at 0 h, 24 h, and 48 h, with MRI measurements taken before and after each injection, followed by a final measurement at 72 h. The bars represent the volume-averaged T_1 and T_2 values for each organ. Each dot within a bar corresponds to the average T_1 and T_2 values of a region of interest (ROI, a voxel in the MRI image). For each organ at each time point, data were collected from 5 to 8 transverse plane MRI slices, with 12–24 ROIs analyzed per slice, resulting in a total of 60–192 ROIs per organ. Absolute T_1 and T_2 values were extracted directly from MRI T_1 and T_2 maps using a self-developed MATLAB code. Statistical significance was accessed using ordinary one-way ANOVA to compare means across time points, with significance levels denoted (*, $P < 0.05$; **, $P < 0.01$; ***, $P < 0.001$; and ***, $P < 0.0001$). All statistical analyses and graphical representations were performed using GraphPad Prism 10.

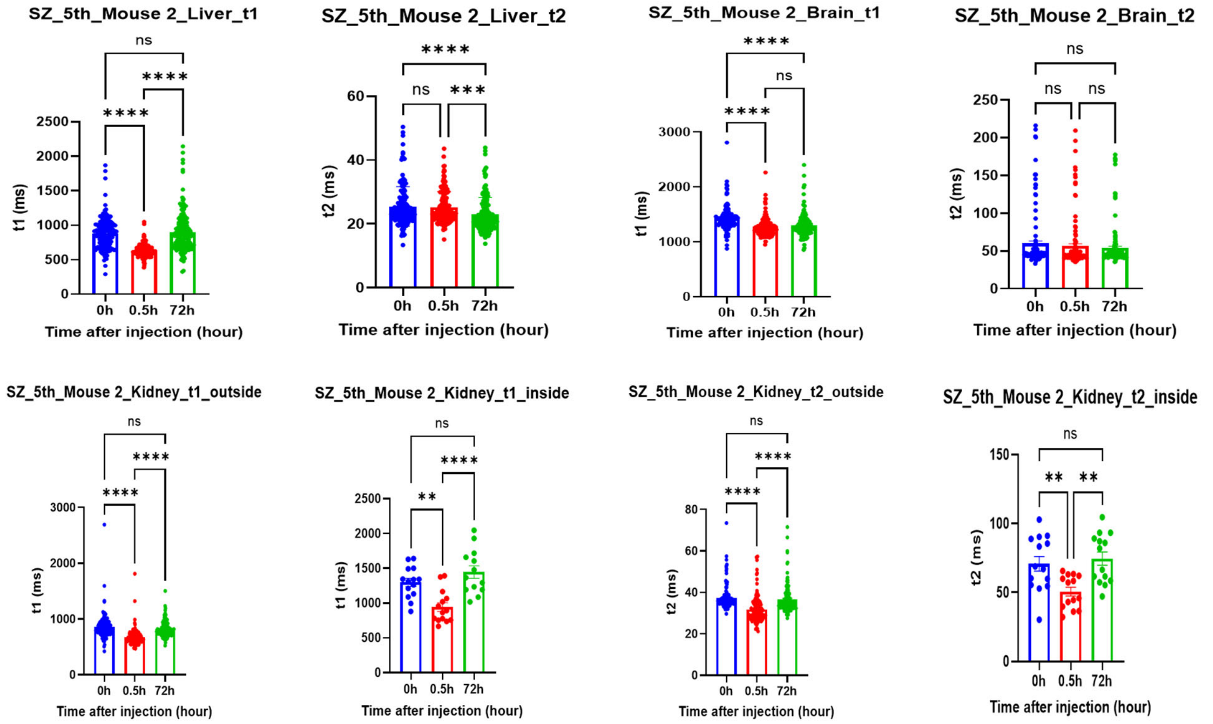

Fig. S30. T_1 and T_2 relaxation times before and after injections of mORCA **2** based on in vivo MRI imaging for the liver, brain, and kidney (mouse label: SZ_5th_Mouse2). Three injections were administered at 0 h, 24 h, and 48 h, with MRI measurements taken pre-injection at 0 h, post-injection at 0.5 h, and finally at 72 h. The bars represent the volume-averaged T_1 and T_2 times for each organ. Each dot within a bar corresponds to the average T_1 or T_2 time of a region of interest (ROI, a voxel in the MRI image). For each organ at each time point, data were collected from 5 to 8 transverse plane MRI slices, with 12–24 ROIs analyzed per slice, resulting in a total of 60–192 ROIs per organ. Absolute T_1 and T_2 values were extracted directly from MRI T_1 and T_2 maps using a self-developed MATLAB code. Statistical significance was accessed using ordinary one-way ANOVA to compare means across time points, with significance levels denoted (*, $P < 0.05$; **, $P < 0.01$; ***, $P < 0.001$; and ***, $P < 0.0001$). All statistical analyses and graphical representations were performed using GraphPad Prism 10.

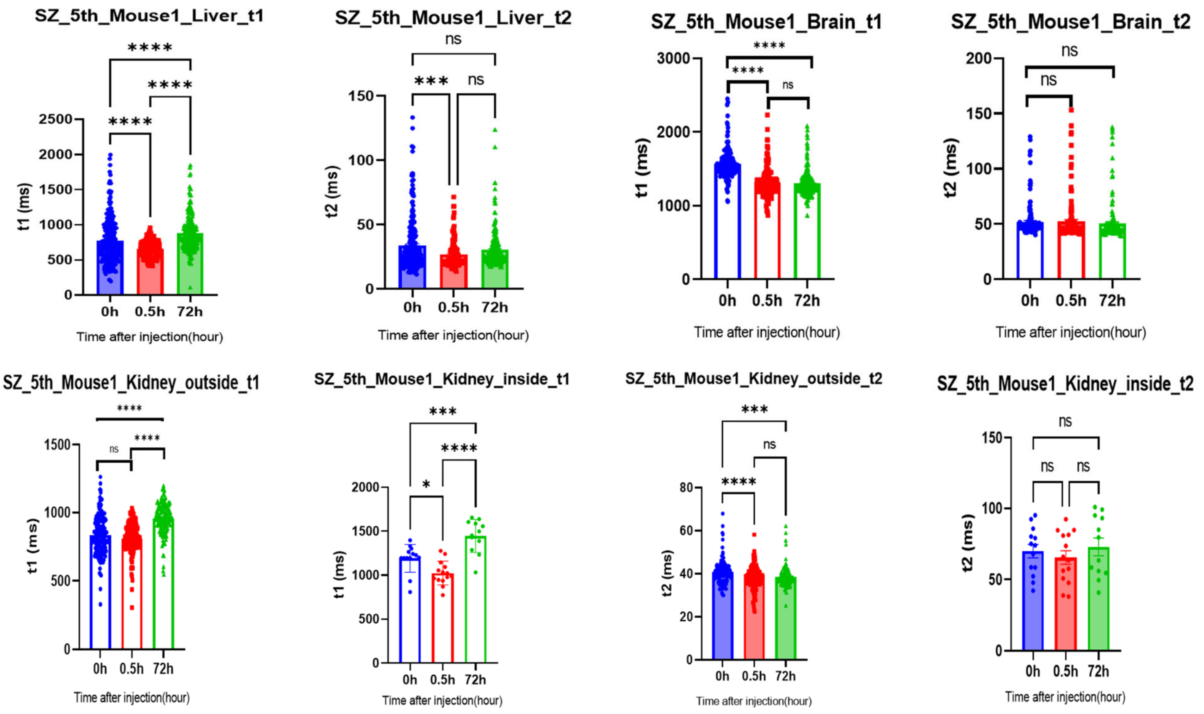

Fig. S31. T_1 and T_2 relaxation times before and after injections of mORCA **2** based on in vivo MRI imaging for the liver, brain, and kidney (mouse label: SZ_5th_mouse1). Three injections were administered at 0 h, 24 h, and 48 h, with MRI measurements taken pre-injection at 0 h, post-injection at 0.5 h, and finally at 72 h. The bars represent the volume-averaged T_1 and T_2 times for each organ. Each dot within a bar corresponds to the average T_1 or T_2 time of a region of interest (ROI, a voxel in the MRI image). For each organ at each time point, data were collected from 5 to 8 transverse plane MRI slices, with 12–24 ROIs analyzed per slice, resulting in a total of 60–192 ROIs per organ. Absolute T_1 and T_2 values were extracted directly from MRI T_1 and T_2 maps using a self-developed MATLAB code. Statistical significance was accessed using ordinary one-way ANOVA to compare means across time points, with significance levels denoted (*, $P < 0.05$; **, $P < 0.01$; ***, $P < 0.001$; and ***, $P < 0.0001$). All statistical analyses and graphical representations were performed using GraphPad Prism 10.

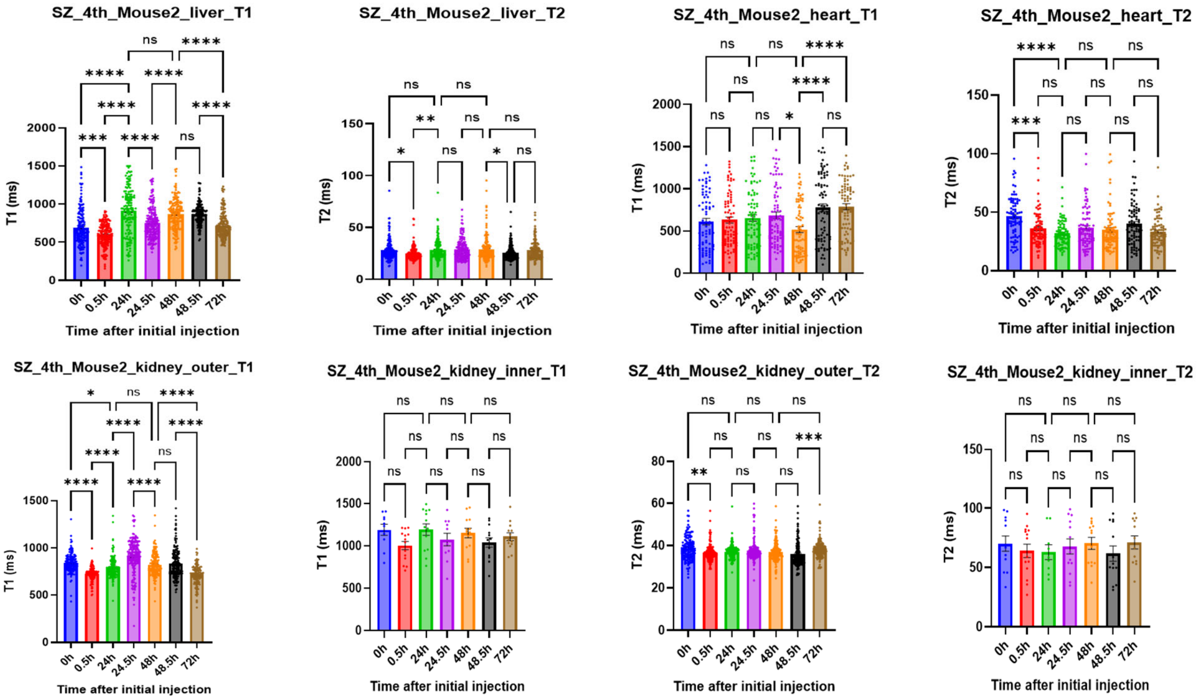

Fig. S32. T_1 and T_2 relaxation times before and after injections of mORCA **2** based on in vivo MRI imaging for the liver, heart, and kidney (mouse label: SZ_4th_mouse2). Three injections were administered at 0 h, 24 h, and 48 h, with MRI measurements taken pre-injection at 0 h, post-injection at 0.5 h, and finally at 72 h. The bars represent the volume-averaged T_1 and T_2 times for each organ. Each dot within a bar corresponds to the average T_1 or T_2 time of a region of interest (ROI, a voxel in the MRI image). For each organ at each time point, data were collected from 5 to 8 transverse plane MRI slices, with 12–24 ROIs analyzed per slice, resulting in a total of 60–192 ROIs per organ. Absolute T_1 and T_2 values were extracted directly from MRI T_1 and T_2 maps using a self-developed MATLAB code. Statistical significance was accessed using ordinary one-way ANOVA to compare means across time points, with significance levels denoted (*, $P < 0.05$; **, $P < 0.01$; ***, $P < 0.001$; and ***, $P < 0.0001$). All statistical analyses and graphical representations were performed using GraphPad Prism 10.

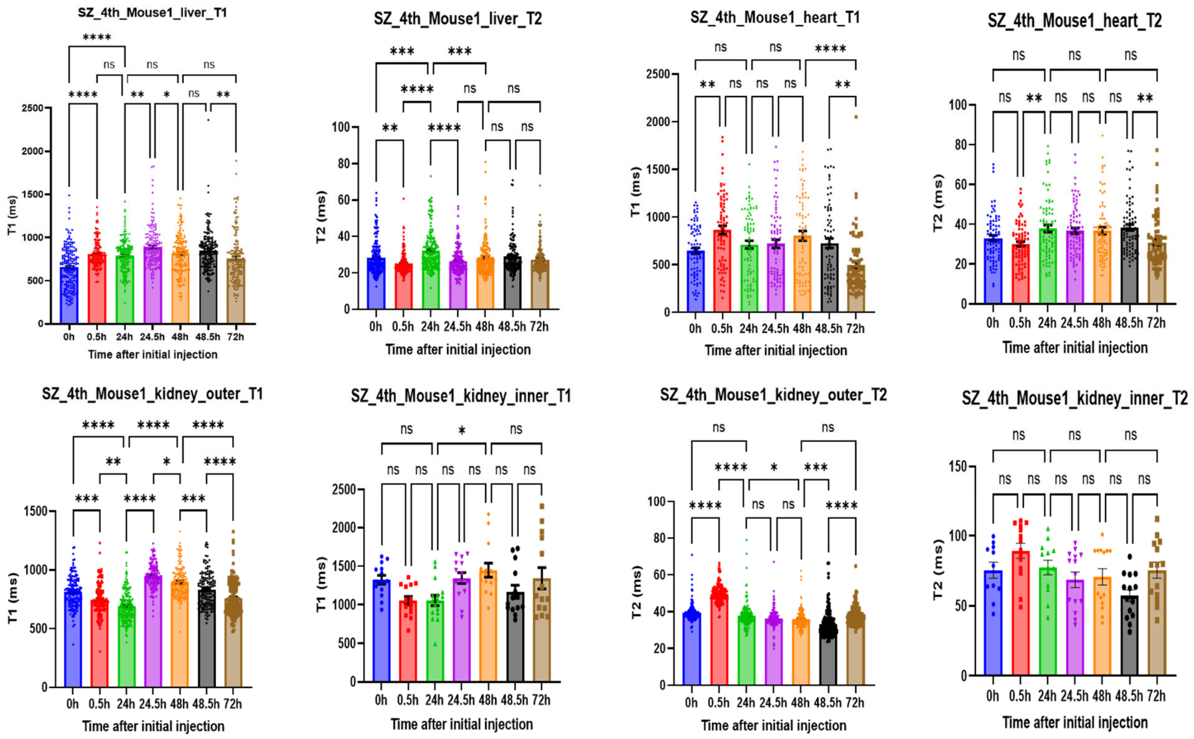

Fig. S33. T_1 and T_2 relaxation times before and after injections of mORCA **2** based on in vivo MRI imaging for the liver, heart, and kidney (mouse label: SZ_4th_mouse1). Three injections were administered at 0 h, 24 h, and 48 h, with MRI measurements taken pre-injection at 0 h, post-injection at 0.5 h, and finally at 72 h. The bars represent the volume-averaged T_1 and T_2 times for each organ. Each dot within a bar corresponds to the average T_1 or T_2 time of a region of interest (ROI, a voxel in the MRI image). For each organ at each time point, data were collected from 5 to 8 transverse plane MRI slices, with 12–24 ROIs analyzed per slice, resulting in a total of 60–192 ROIs per organ. Absolute T_1 and T_2 values were extracted directly from MRI T_1 and T_2 maps using a self-developed MATLAB code. Statistical significance was accessed using ordinary one-way ANOVA to compare means across time points, with significance levels denoted (*, $P < 0.05$; **, $P < 0.01$; ***, $P < 0.001$; and ***, $P < 0.0001$). All statistical analyses and graphical representations were performed using GraphPad Prism 10.

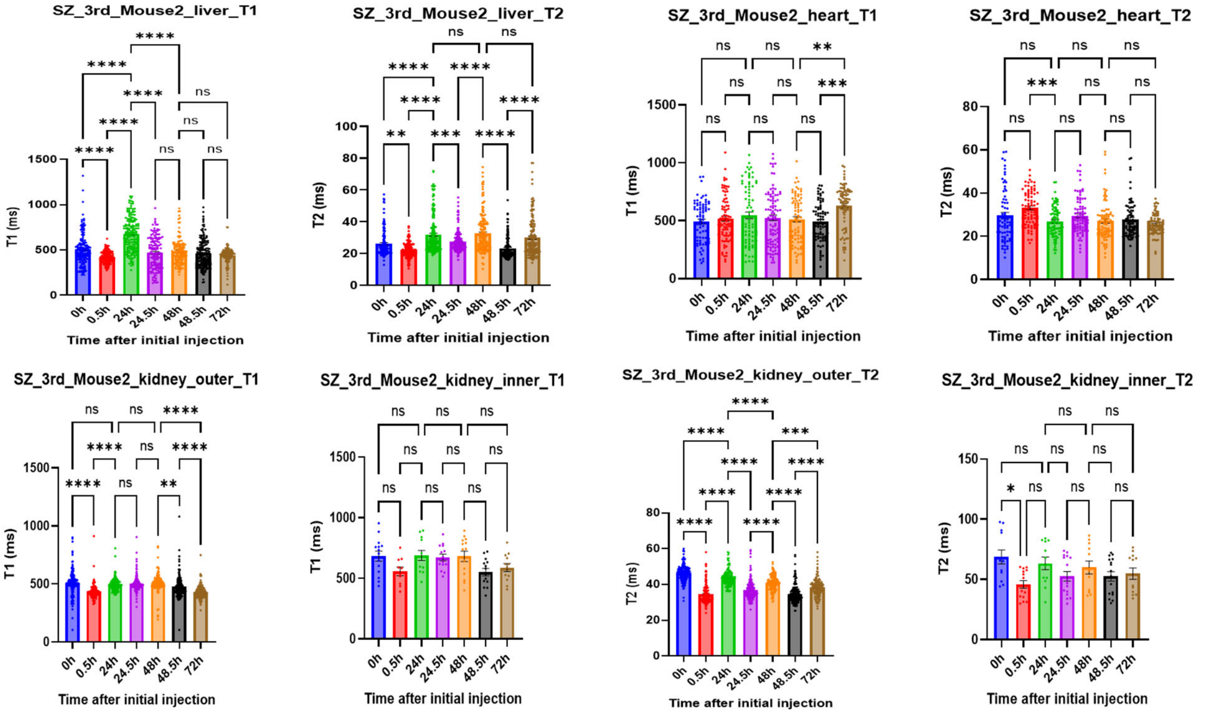

Fig. S34. T_1 and T_2 relaxation times before and after injections of mORCA **2** based on in vivo MRI imaging for the liver, heart, and kidney (mouse label: SZ_3rd_mouse2). Three injections were administered at 0 h, 24 h, and 48 h, with MRI measurements taken pre-injection at 0 h, post-injection at 0.5 h, and finally at 72 h. The bars represent the volume-averaged T_1 and T_2 times for each organ. Each dot within a bar corresponds to the average T_1 or T_2 time of a region of interest (ROI, a voxel in the MRI image). For each organ at each time point, data were collected from 5 to 8 transverse plane MRI slices, with 12–24 ROIs analyzed per slice, resulting in a total of 60–192 ROIs per organ. Absolute T_1 and T_2 values were extracted directly from MRI T_1 and T_2 maps using a self-developed MATLAB code. Statistical significance was accessed using ordinary one-way ANOVA to compare means across time points, with significance levels denoted (*, $P < 0.05$; **, $P < 0.01$; ***, $P < 0.001$; and ***, $P < 0.0001$). All statistical analyses and graphical representations were performed using GraphPad Prism 10.

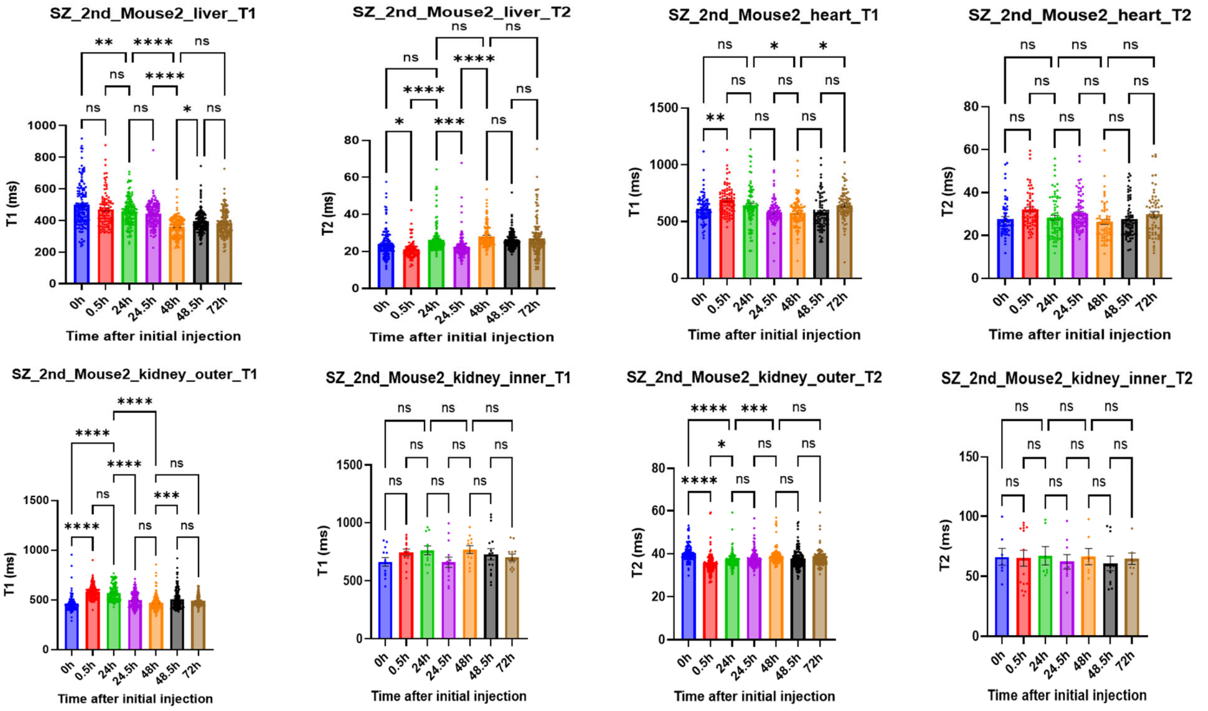

Fig. S35. T_1 and T_2 relaxation times before and after injections of mORCA **2** based on in vivo MRI imaging for the liver, heart, and kidney (mouse label: SZ_2nd_mouse2). Three injections were administered at 0 h, 24 h, and 48 h, with MRI measurements taken pre-injection, post-injection, and finally at 72 h. The bars represent the volume-averaged T_1 and T_2 times for each organ. Each dot within a bar corresponds to the average T_1 or T_2 time of a region of interest (ROI, a voxel in the MRI image). For each organ at each time point, data were collected from 5 to 8 transverse plane MRI slices, with 12–24 ROIs analyzed per slice, resulting in a total of 60–192 ROIs per organ. Absolute T_1 and T_2 values were extracted directly from MRI T_1 and T_2 maps using a self-developed MATLAB code. Statistical significance was accessed using ordinary one-way ANOVA to compare means across time points, with significance levels denoted (*, $P < 0.05$; **, $P < 0.01$; ***, $P < 0.001$; ***, $P < 0.0001$). All statistical analyses and graphical representations were performed using GraphPad Prism 10.

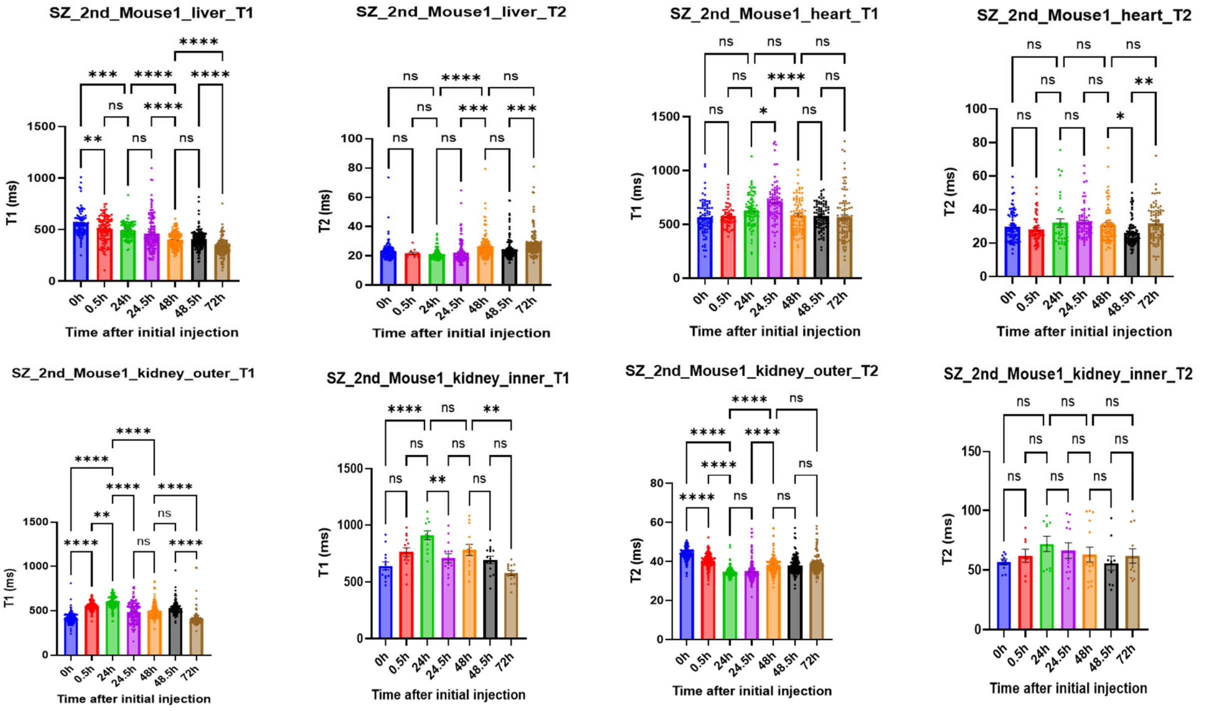

Fig. S36. T_1 and T_2 relaxation times before and after injections of mORCA **2** based on in vivo MRI imaging for the liver, heart, and kidney (mouse label: SZ_2nd_mouse1). Three injections were administered at 0 h, 24 h, and 48 h, with MRI measurements taken pre-injection, post-injection, and finally at 72 h. The bars represent the volume-averaged T_1 and T_2 times for each organ. Each dot within a bar corresponds to the average T_1 or T_2 time of a region of interest (ROI, a voxel in the MRI image). For each organ at each time point, data were collected from 5 to 8 transverse plane MRI slices, with 12–24 ROIs analyzed per slice, resulting in a total of 60–192 ROIs per organ. Absolute T_1 and T_2 values were extracted directly from MRI T_1 and T_2 maps using a self-developed MATLAB code. Statistical significance was accessed using ordinary one-way ANOVA to compare means across time points, with significance levels denoted (*, $P < 0.05$; **, $P < 0.01$; ***, $P < 0.001$; and ***, $P < 0.0001$). All statistical analyses and graphical representations were performed using GraphPad Prism 10.

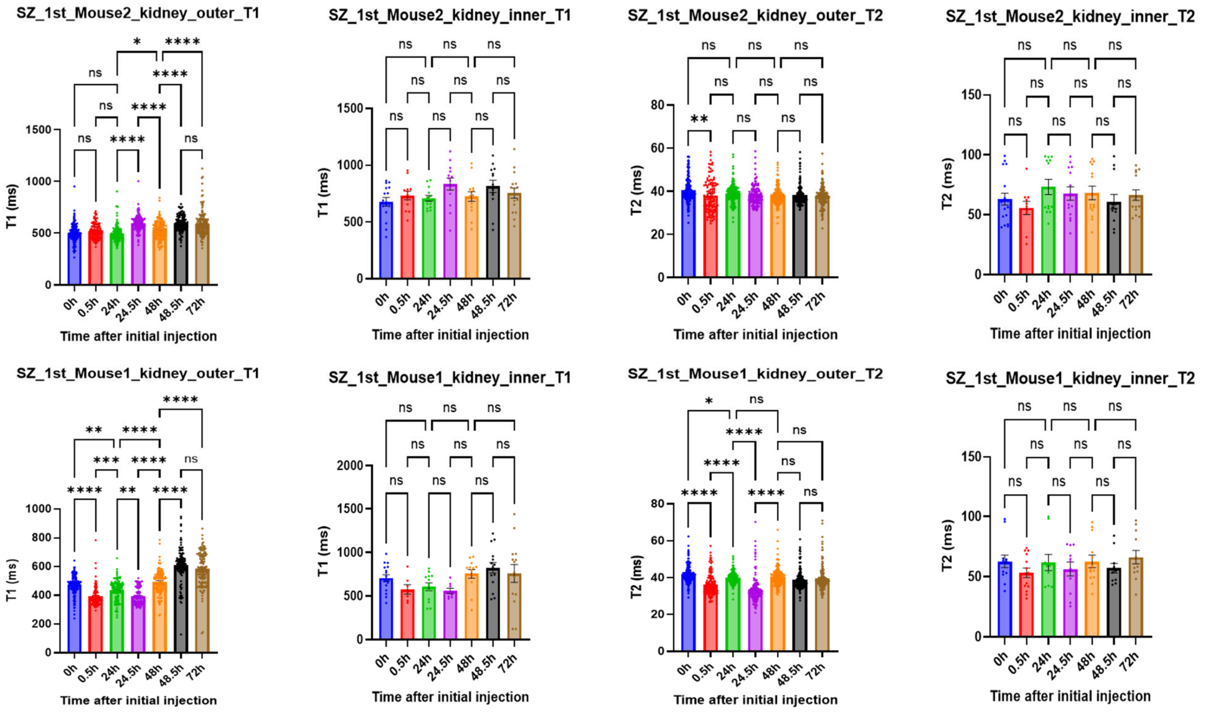

Fig. S37. T_1 and T_2 relaxation times before and after injections of mORCA **2** based on in vivo MRI imaging for the kidney (mouse label: SZ_1st_mouse1 and SZ_1st_mouse2). Three injections were administered at 0 h, 24 h, and 48 h, with MRI measurements taken pre-injection, post-injection, and finally at 72 h. The bars represent the volume-averaged T_1 and T_2 times for each organ. Each dot within a bar corresponds to the average T_1 or T_2 time of a region of interest (ROI, a voxel in the MRI image). For each organ at each time point, data were collected from 5 to 8 transverse plane MRI slices, with 12–24 ROIs analyzed per slice, resulting in a total of 60–192 ROIs per organ. Absolute T_1 and T_2 values were extracted directly from MRI T_1 and T_2 maps using a self-developed MATLAB code. Statistical significance was accessed using ordinary one-way ANOVA to compare means across time points, with significance levels denoted (*, $P < 0.05$; **, $P < 0.01$; ***, $P < 0.001$; and ***, $P < 0.0001$). All statistical analyses and graphical representations were performed using GraphPad Prism 10.

4. Summary of ex vivo EPR spectroscopy.

The ex vivo quantitative EPR spectroscopic analysis of the biodistribution for most tissues (Figure 6, main text; Figs. S38 and S39, SI) is performed on four mice injected with agent **2** (SZ527, SZ530, SZ538, and SZ540) and two mice (SD129 and SD130) injected with agent **1** (with the nicotinate moiety). The mice are sacrificed 72 h after the initial agent injection (and 24 h after the final 3rd injection) and immediately following the final imaging through MRI (Table S3). The spectra are obtained at 243 K in a frozen buffer in a 4 mm OD EPR quartz tube to provide sufficient S/N; selected spectra are simulated as summarized in Table S4.

Another set of spectra are obtained at 294 K, i.e., in a fluid buffer. The simulated spectra show the presence of two components: the major one is the slow-moving nitroxide radical with τ_{cor} in the ns range, and the minor one is simulated as the fast-moving nitroxide radical with τ_{cor} fixed to 5 ps. Notably, blood is simulated with the slow-moving nitroxide radical only. Typical simulations used *g*-and *A*-tensors from simulations at 243 K. Results are summarized in Table S5.

Table S3. Summary of mice used for in vivo MRI, which are sacrificed for ex vivo EPR spectroscopy.

mORCA	Initial injection of the agent	Mouse 1 (M1)	Mouse 2 (M2)	Notes
**2**	June 15th	SZ527	SZ530	M2 3rd i.v. failed; i.p. instead
**1**	July 12th	SD129	SD130	
**2**	July 26th	SZ538	SZ540	M1 3rd i.v. failed; i.p. instead

Table S4. Summary of spectral simulations (EasySpin *pepper* suite) for selected spectra of tissues at 243 K, presented in Figures 5A-C (main text) and S41 – S45, SI.

Figures	Mouse label	Tissue	mORCA	EPR label	Freq (GHz)	rmsd	τ_{cor}	A_{zz}[a] (MHz)	g_{zz}	g_{yy}	g_{xx}	*H*-strains (MHz)		
												zz	yy	xx
S41A	SZ540M2	Blood	**2**	SZ540r18	9.3187	0.001917	rigid	96.8	2.0030	2.0053	2.0090	30±0	22±1	22±1
S42A	SZ530M2	Kidney	**2**	SZ530r01	9.3200	0.004809	rigid	99±1	2.0039	2.0046	2.0069	56±1	26±4	23±2
S43A	SD129M1	Brain	**1**	SD129r05	9.3263	0.001637	rigid	95±2	2.0043	2.0054	2.0065	27±3	28±11	32±14
S44A	SD129M1	Liver	**1**	SD129r20	9.3242	0.008340	rigid	94±1	2.0042	2.0047	2.0064	50±1	25±4	21±3
S45A	SD129M1	Lung	**1**	SD129r07	9.3227	0.002104	rigid	93±1	2.0040	2.0044	2.0077	43±1	21±4	20±1

[a] Other components of the ^{14}N *A*-tensor were fixed to A_{yy} = 16.8 and A_{xx} = 15.0 MHz to minimize the over-parametrization of the fit.

Table S5. Summary of EPR spectral simulations (EasySpin *chili* suite) for spectra of tissues at 294 K, with focus on τ_{cor}.

Tissue	Agent	Animal label	EPR label	τ_{cor} (ns)	Slow-to-fast (5 ps) ratio	Rmsd	Figure
Blood	**1**	SD129M1	SD132R15	3.8±0.1	1.00/0.00	0.001289	S46
Blood	**1**	SD129M1	SD132R16	4.7±0.1	1.00/0.00	0.000664	S47
Blood	**1**	SD130M2	SD132R14	2.6±0.1	1.00/0.00	0.000195	S48
Blood	**2**	SZ527M1	SZ528R06	3.8±0.0	1.00/0.00	0.000544	S49
Blood	**2**	SZ540M2	SZ550R01	4.1±0.1	1.00/0.00	0.001346	S50
Blood	**2**	SZ540M2	SZ550R02	4.8±0.1	1.00/0.00	0.001397	S51
Blood	**2**	SZ540M2	SZ550R04	4.4±0.1	1.00/0.00	0.001300	5D, main text, S41B, S52
Kidney	**1**	SD129M1	SD130R06	3.1±0.0	1.00/0.32	0.001164	S53
Kidney	**1**	SD130M2	SD132R01	6.5±0.9	0.64	0.001050	S54
Kidney	**1**	SD130M2	SD132R02	4.8±0.0	0.10	0.000415	S55
Kidney	**1**	SD130M2	SD132R38	6.3±0.8	0.65	0.003322	S56
Kidney	**1**	SD130M2	SD132R40	6.4±1.0	0.52	0.003369	S57
Kidney	**1**	SD130M2	SD132R41	5.0±0.7	0.58	0.001874	S58
Kidney	**2**	SZ530M2	SZ531R02	7.3±0.5	0.23	0.000830	5E, main text, S42B, S59
Kidney	**2**	SZ530M2	SZ531R03	4.6±0.5	0.49	0.000167	S60
Kidney	**2**	SZ540M2	SZ550R09	7.5±1.8	0.25	0.001625	S61
Kidney	**2**	SZ540M2	SZ550R11	8.4±2.2	0.21	0.001763	S62
Liver	**1**	SD129M1	SD130R13	8.8±0.9	0.40	0.002189	S44B, S63
Liver	**1**	SD129M1	SD130R14	9.8±0.0	0.45	0.001769	S64
Liver	**1**	SD130M2	SD132R11	8.4±1.3	0.22	0.000783	S65
Liver	**2**	SZ530M2	SZ531R22	9.8±0.7	0.40	0.002176	S66
Lung	**1**	SD129M1	SD130R07	4.2±0.0	0.53	0.001337	S45B, S67
Lung	**1**	SD129M1	SD130R08	4.7±0.3	0.44	0.000738	S68
Lung	**1**	SD130M2	SD132R05	6.5±0.2	0.57	0.000495	S69
Lung	**1**	SD130M2	SD132R44	6.7±1.8	0.49	0.001912	S70
Brain	**1**	SD129M1	SD130R18	3.9±0.2	0.73	0.000603	5F, main text; S43B

D

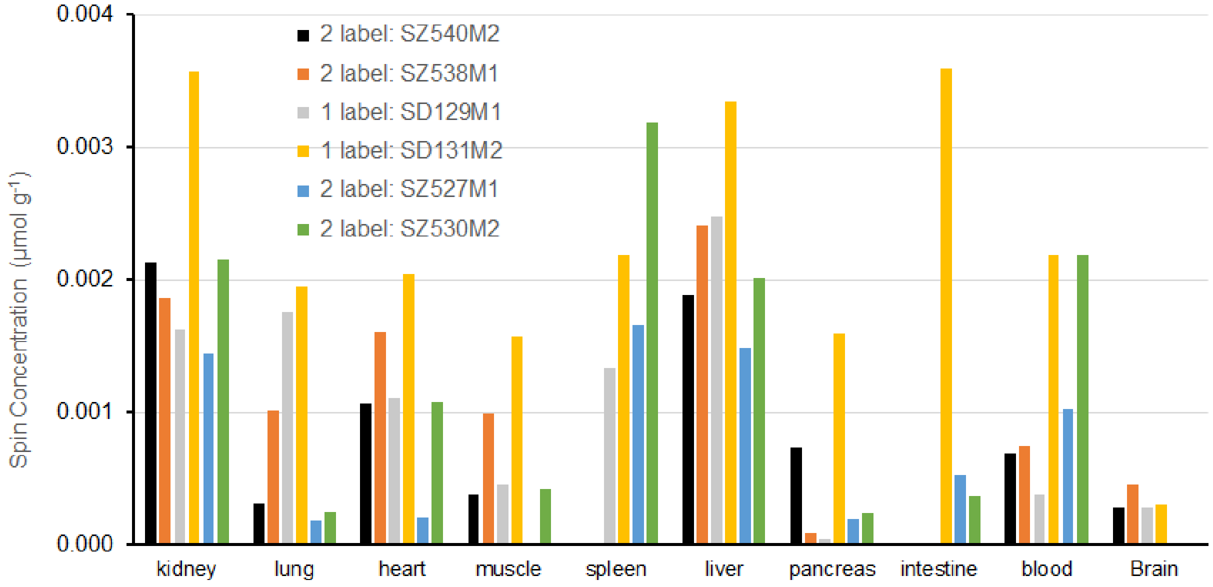

Fig. S38. Ex vivo EPR spectroscopic quantification is carried out 72 h after the initial injection of contrast agents **1** (2 mice) and **2** (4 mice). Spin concentrations in tissues (µmol/g) in individual mice with $n = 2$ for **1** and $n = 4$ for **2**, except for the brain, intestine, and spleen where $n = 2$ for both **1** and **2**. Mouse labels are summarized in Table S3.

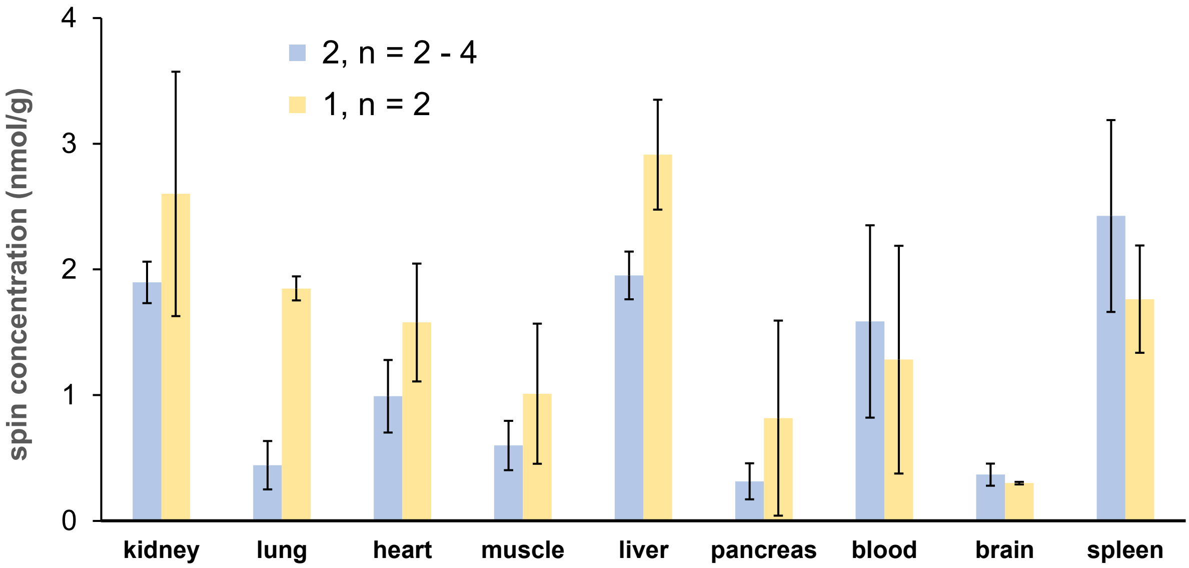

Fig. S39. Biodistribution of nitroxide radical as the spin concentration in tissues (nmol/g), based on ex vivo EPR spectroscopic quantification, which is carried out 72 h after the initial injection and 24 h after the last injection of mORCA **1** (orange bars) or **2** (blue bars). Each animal received three 6 mg doses of the mORCA spaced by 24 h. The

reported values represent the mean ± SEM, with n = 2 for **1** and n = 4 for **2**, except for the muscle, brain, and spleen for **2** where n = 3, n = 2, and n = 2, respectively.

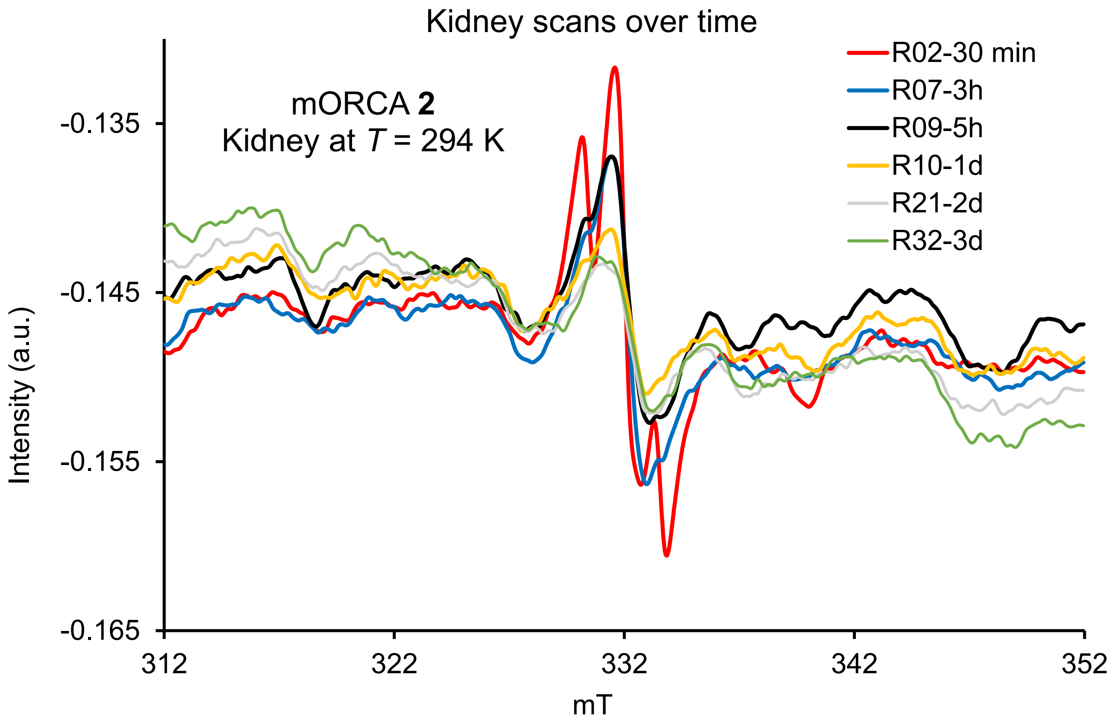

Fig. S40. Ex vivo EPR spectra (raw data) for the kidney homogenate/PBS at 294 K vs. time at room temperature (mouse label: SZ530M2; EPR labels: SZ531R02, R07, R09, R10, R21, and R32). Initial EPR spectrum (30 min, EPR label: SZ531R02) following background subtraction and baseline correction is summarized in Table S5 and presented in Figure 5E (main text) and Figs. S42B and S59.

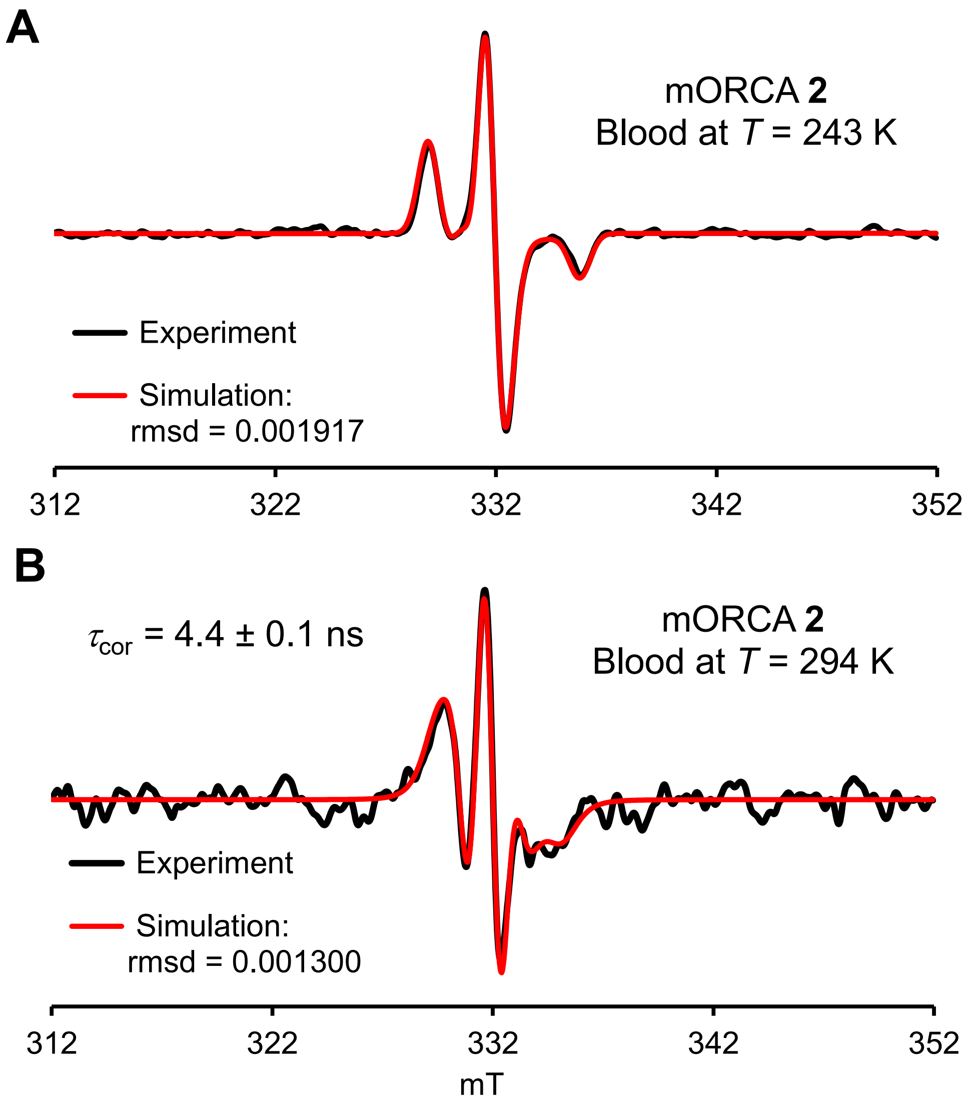

Fig. S41. This figure is identical to that in the main text (Figure 5A and D). Ex vivo EPR spectra of homogenized blood (diluted with PBS). **A**: Blood at 243 K (mouse label: SZ540M2; EPR label: SZ540R18). **B**: Blood at 294 K (mouse label: SZ540M2; EPR label: SZ550R04). Spectra at 243 K and 294 K are obtained in 4 mm OD EPR quartz tubes and 0.6 mm ID EPR quartz capillaries, respectively. Spectral simulations are carried out with the EasySpin *pepper* (A) and *chili* (B) suites; key simulation parameters are summarized in Tables S4 and S5.

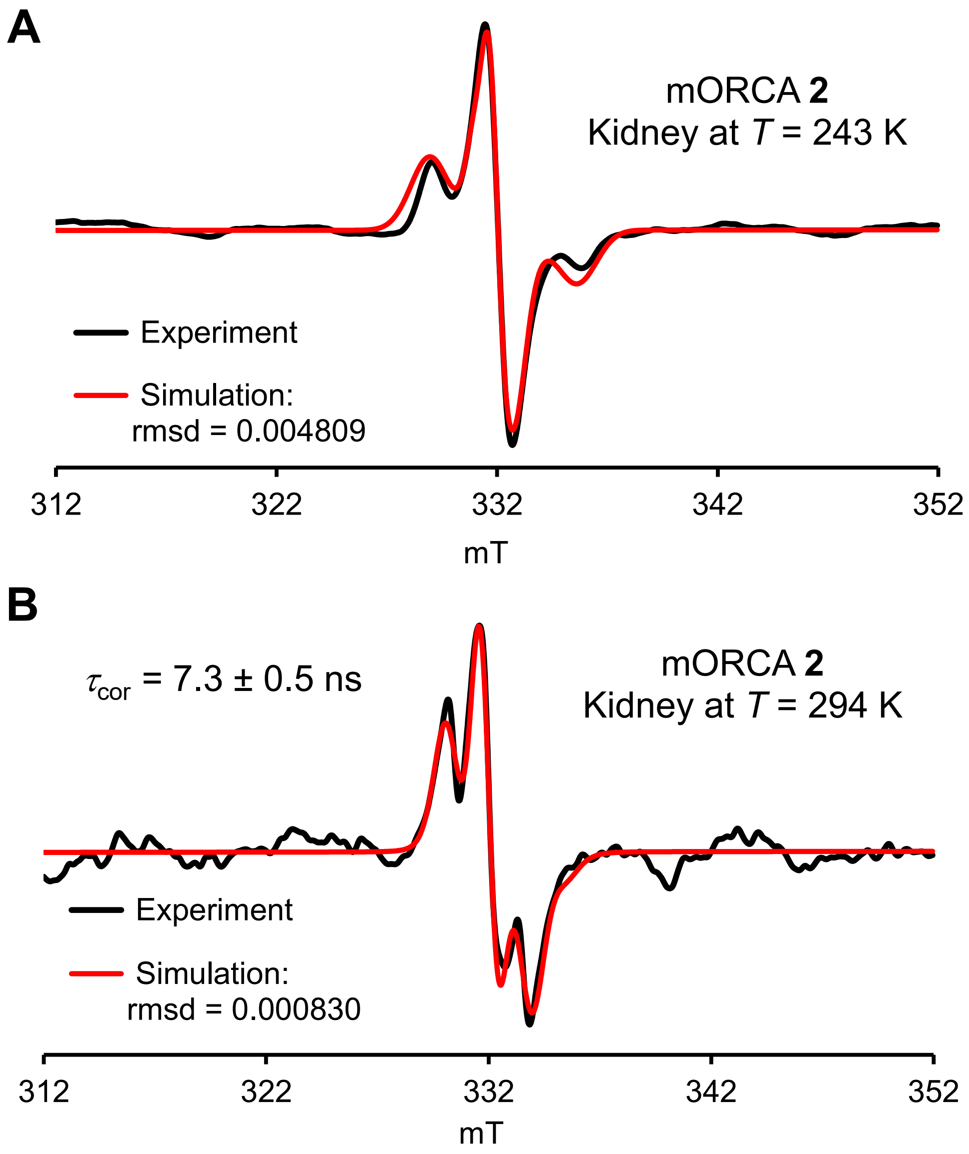

Fig. S42. This figure is identical to that in the main text (Figure 5B and E). Ex vivo EPR spectra of the homogenized kidney (diluted with PBS). **A**: The kidney at 243 K (mouse label: SZ530M2; EPR label: SZ530R1). **B**: The kidney at 294 K (mouse label: SZ530M2; EPR label: SZ531R02). Spectra at 243 K and 294 K are obtained in 4 mm OD EPR quartz tubes and 0.6 mm ID EPR quartz capillaries, respectively. Spectral simulations are carried out with the EasySpin *pepper* (A) and *chili* (B) suites; key simulation parameters are summarized in Tables S4 and S5.

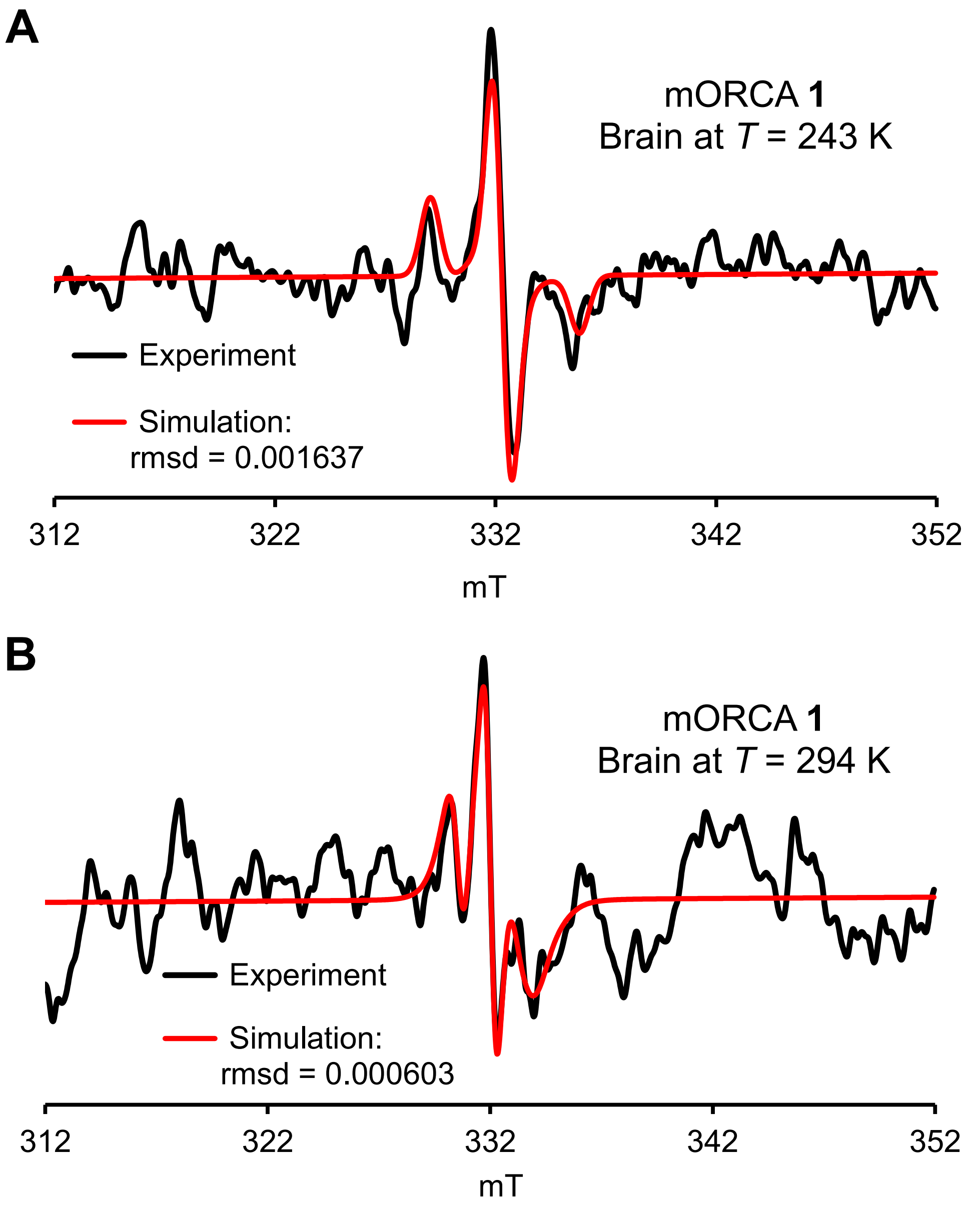

Fig. S43. This figure is identical to that in the main text (Figure 5C and F). Ex vivo EPR spectra of the homogenized brain (diluted with PBS). **A**: The brain at 243 K (mouse label: SD129M1; EPR label: SD129r05). **B**: The brain at 294 K (mouse label: SD129M1; EPR label: SD130R18). Spectra at 243 K and 294 K are obtained in 4 mm OD EPR quartz tubes and 0.6 mm ID EPR quartz capillaries, respectively. Spectral simulations are carried out with the EasySpin *pepper* (A) and *chili* (B) suites; key simulation parameters are summarized in Tables S4 and S5.

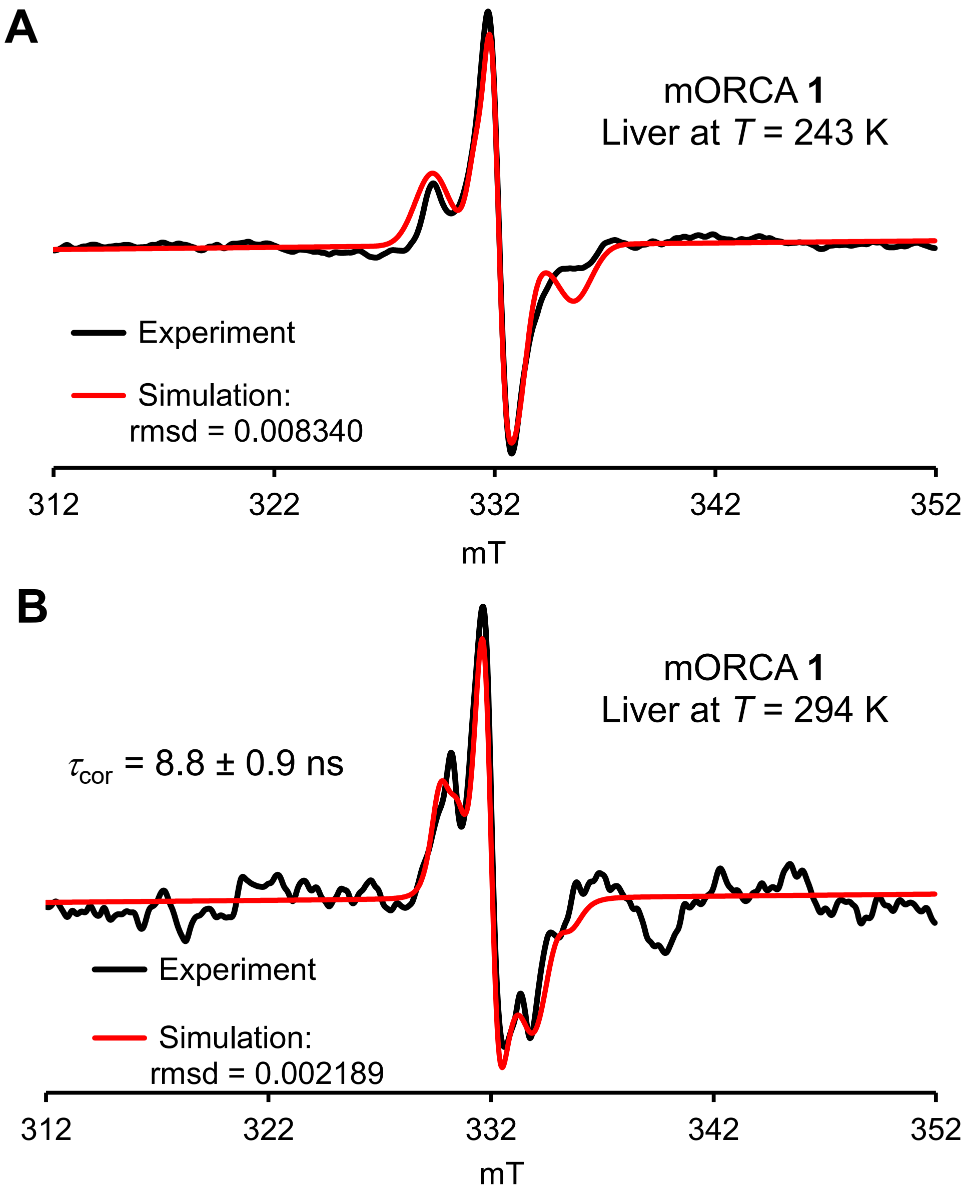

Fig. S44. Ex vivo EPR spectra of the homogenized liver (diluted with PBS). **A**: The liver at 243 K (mouse label: SD129M1; EPR label: SD129r20). **B**: The liver at 294 K (mouse label: SD129M1; EPR label: SD130r13). Spectra at 243 K and 294 K are obtained in 4 mm OD EPR quartz tubes and 0.6 mm ID EPR quartz capillaries, respectively. Spectral simulations are carried out with the EasySpin *pepper* (A) and *chili* (B) suites; key simulation parameters are summarized in Tables S4 and S5.

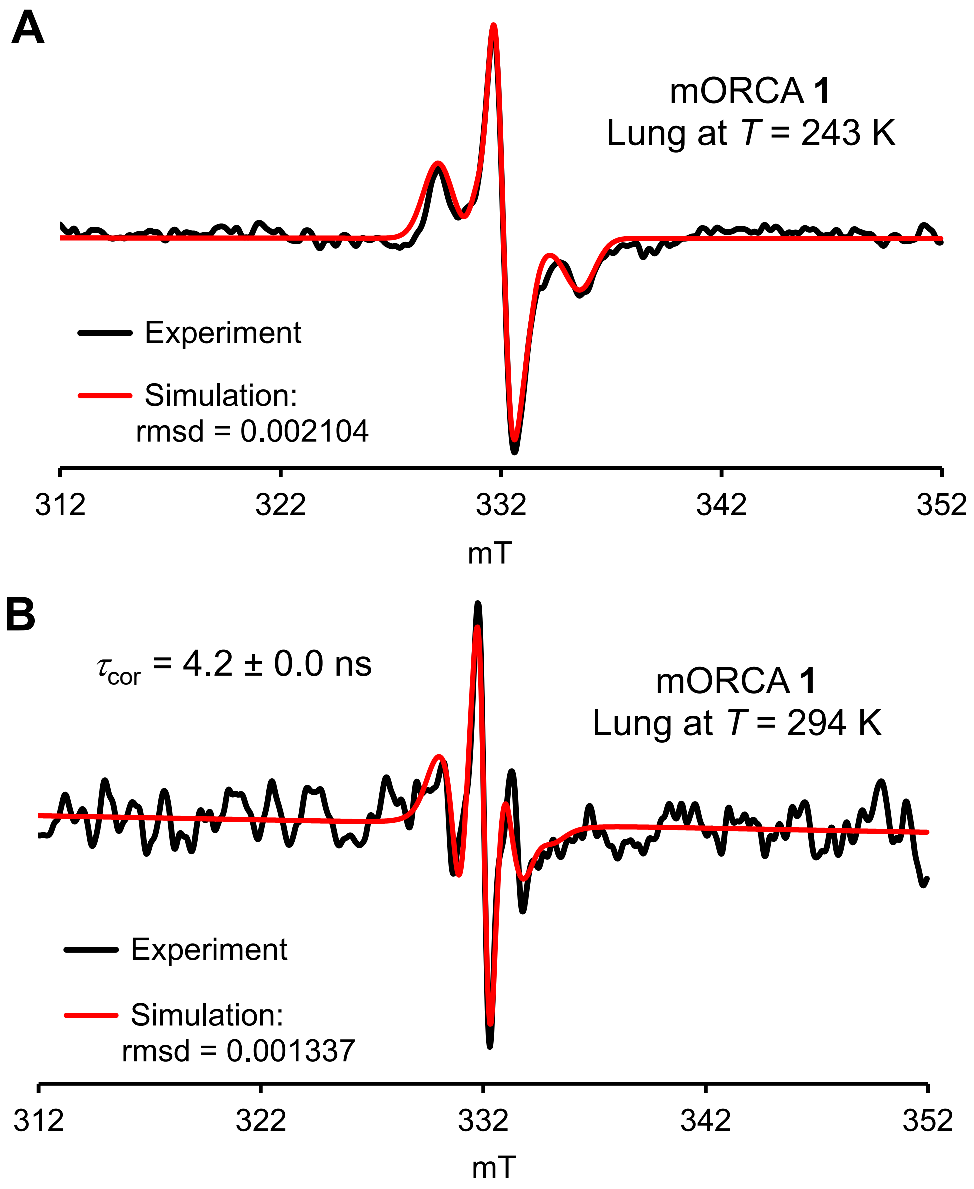

Fig. S45. Ex vivo EPR spectra of the homogenized lung (diluted with PBS). **A**: The lung at 243 K (mouse label: SD129M1; EPR label: SD129r07). **B**: The lung at 294 K (mouse label: SD129M1; EPR label: SD130r07). Spectra at 243 K and 294 K are obtained in 4 mm OD EPR quartz tubes and 0.6 mm ID EPR quartz capillaries, respectively. Spectral simulations are carried out with the EasySpin *pepper* (A) and *chili* (B) suites; key simulation parameters are summarized in Tables S4 and S5.

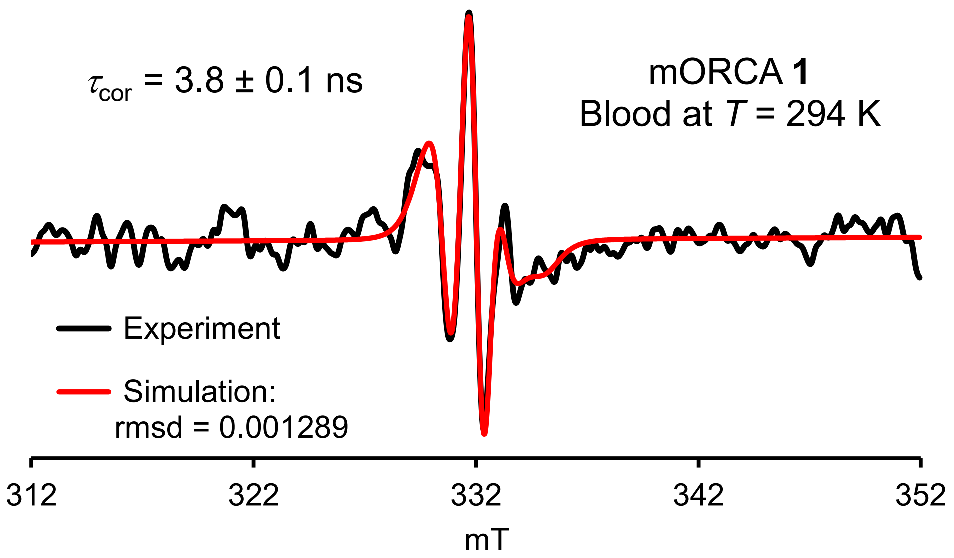

Fig. S46. Ex vivo EPR spectrum of homogenized blood (diluted with PBS) at 294 K (mouse label: SD129M1; EPR label: SD130R15). A 0.6 mm ID EPR quartz capillary is employed. Spectral simulations are carried out with the EasySpin *chili* suite; key simulation parameters are summarized in Table S5.

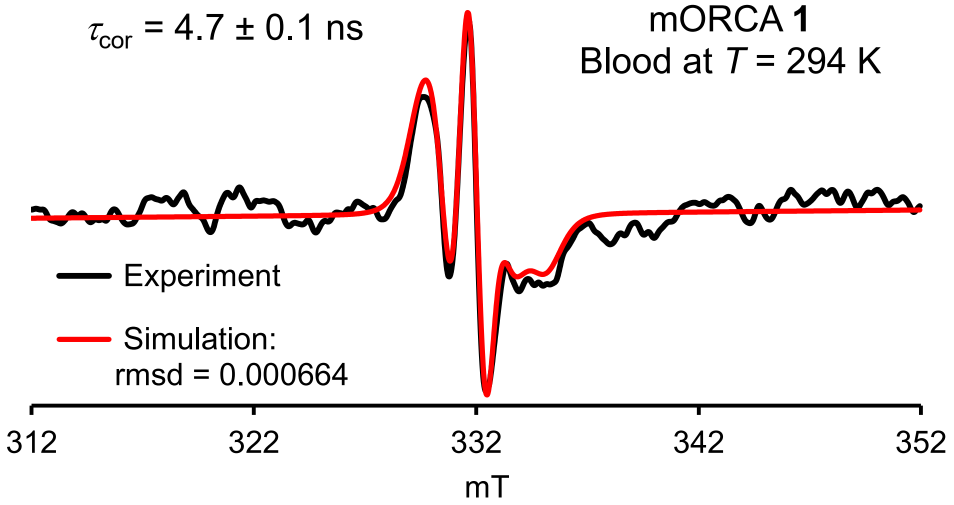

Fig. S47. Ex vivo EPR spectrum of homogenized blood (diluted with PBS) at 294 K (mouse label: SD129M1; EPR label: SD130R16). A 0.6 mm ID EPR quartz capillary is employed. Spectral simulations are carried out with the EasySpin *chili* suite; key simulation parameters are summarized in Table S5.

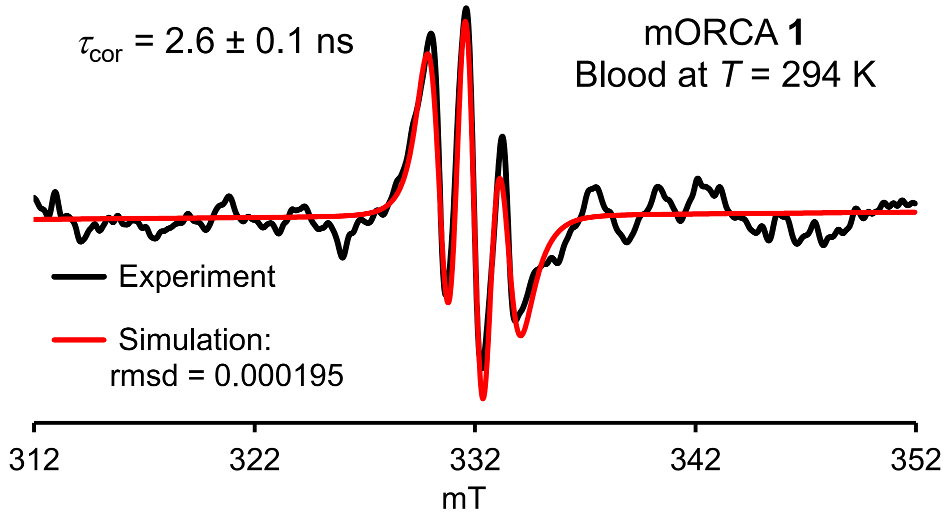

Fig. S48. Ex vivo EPR spectrum of homogenized blood (diluted with PBS) at 294 K (mouse label: SD130M2; EPR label: SD132R14). A 0.6 mm ID EPR quartz capillary is employed. Spectral simulations are carried out with the EasySpin *chili* suite; key simulation parameters are summarized in Table S5.

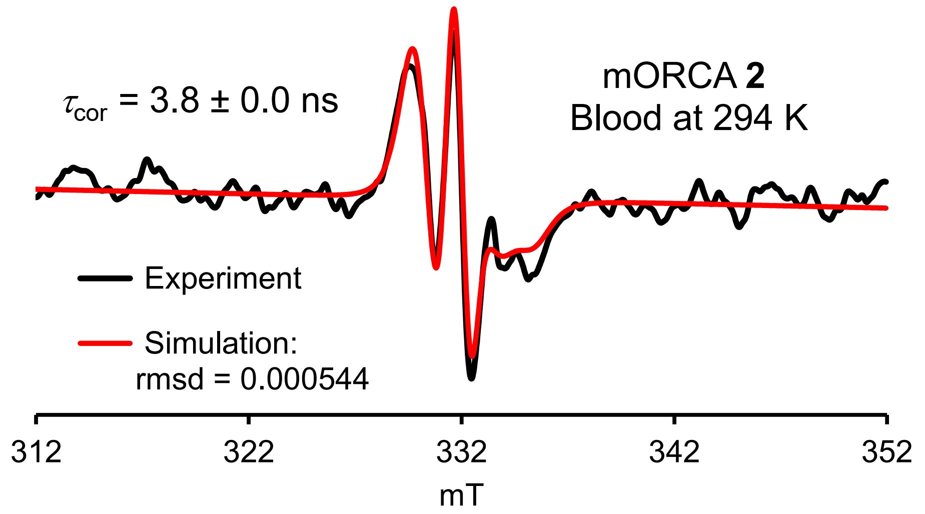

Fig. S49. Ex vivo EPR spectrum of homogenized blood (diluted with PBS) at 294 K (mouse label: SZ527M1; EPR label: SZ528r06). A 0.6 mm ID EPR quartz capillary is employed. Spectral simulations are carried out with the EasySpin *chili* suite; key simulation parameters are summarized in Table S5.

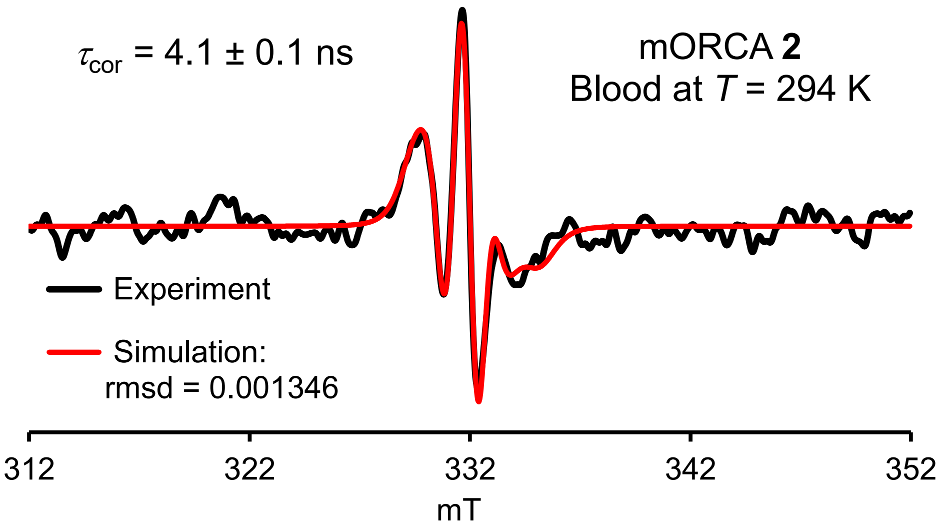

Fig. S50. Ex vivo EPR spectrum of homogenized blood (diluted with PBS) at 294 K (mouse label: SZ540M2; EPR label: SZ550R01). A 0.6 mm ID EPR quartz capillary is employed. Spectral simulations are carried out with the EasySpin *chili* suite; key simulation parameters are summarized in Table S5.

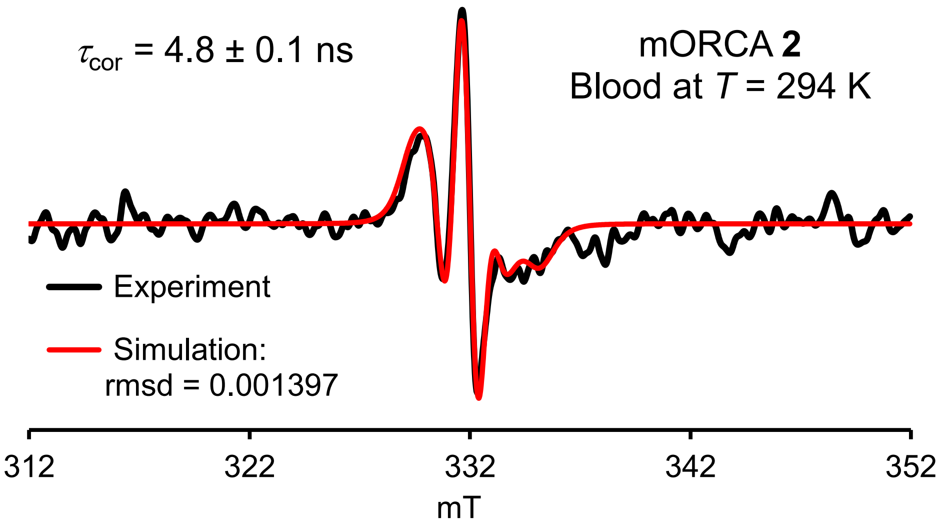

Fig. S51. Ex vivo EPR spectrum of homogenized blood (diluted with PBS) at 294 K (mouse label: SZ540M2; EPR label: SZ550R02). A 0.6 mm ID EPR quartz capillary is employed. Spectral simulations are carried out with the EasySpin *chili* suite; key simulation parameters are summarized in Table S5.

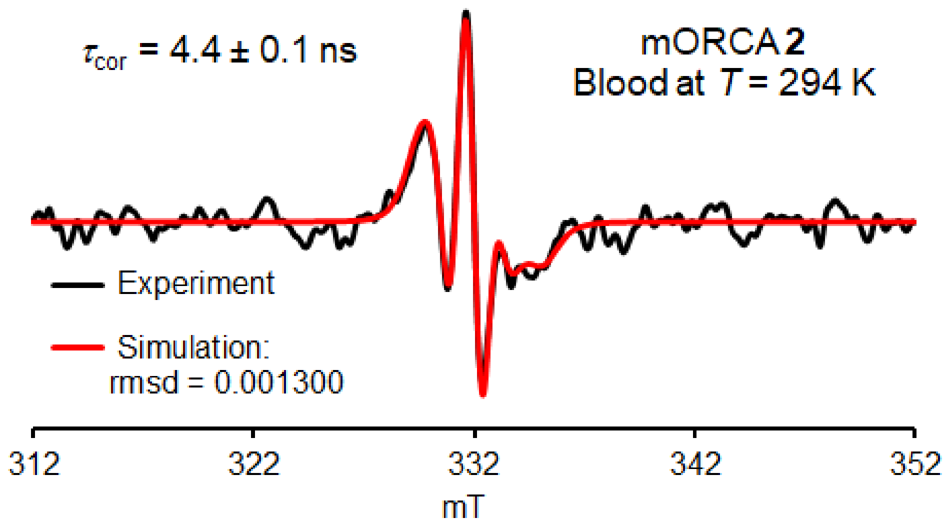

Fig. S52. This figure is identical to that in the main text (Figure 5D). Ex vivo EPR spectrum of homogenized blood (diluted with PBS) at 294 K (mouse label: SZ540M2; EPR label: SZ550R04). Spectral simulations are carried out with the EasySpin *chili* suite; key simulation parameters are summarized in Table S5.

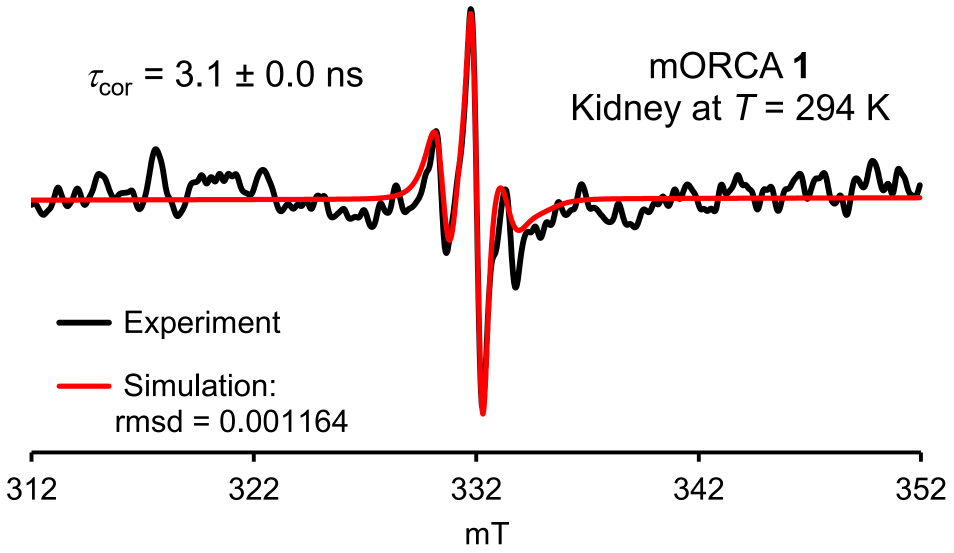

Fig. S53. Ex vivo EPR spectrum of the homogenized kidney (diluted with PBS) at 294 K (mouse label: SD129M1; EPR label: SD130R06). A 0.6 mm ID EPR quartz capillary is employed. Spectral simulations are carried out with the EasySpin *chili* suite; key simulation parameters are summarized in Table S5.

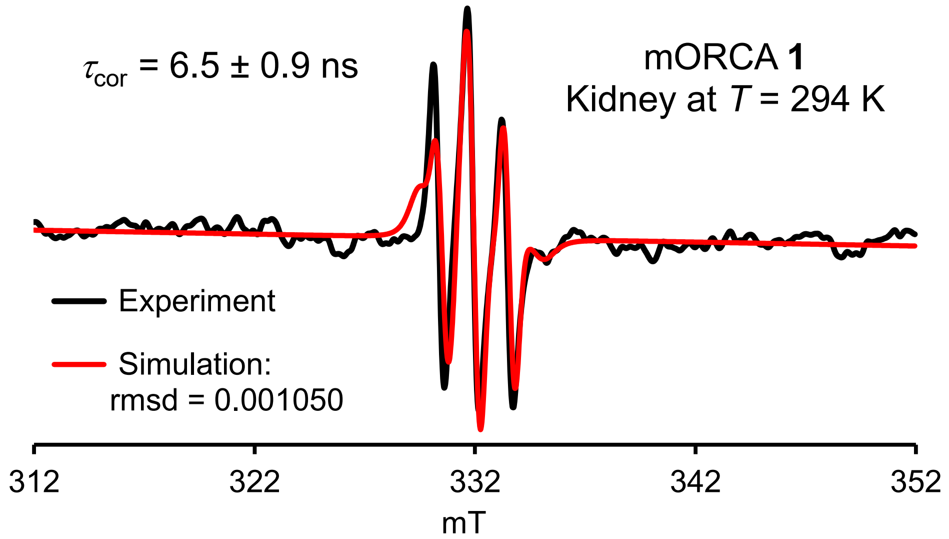

Fig. S54. Ex vivo EPR spectrum of the homogenized kidney (diluted with PBS) at 294 K (mouse label: SD130M2; EPR label: SD132R01). A 0.6 mm ID EPR quartz capillary is employed. Spectral simulations are carried out with the EasySpin *chili* suite; key simulation parameters are summarized in Table S5.

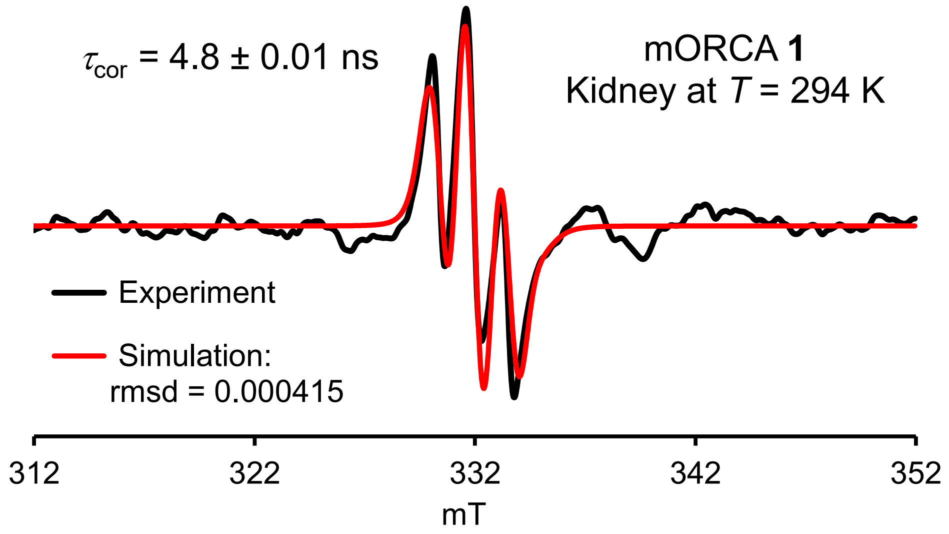

Fig. S55. Ex vivo EPR spectrum of the homogenized kidney (diluted with PBS) at 294 K (mouse label: SD130M2; EPR label: SD132R02). A 0.6 mm ID EPR quartz capillary is employed. Spectral simulations are carried out with the EasySpin *chili* suite; key simulation parameters are summarized in Table S5.

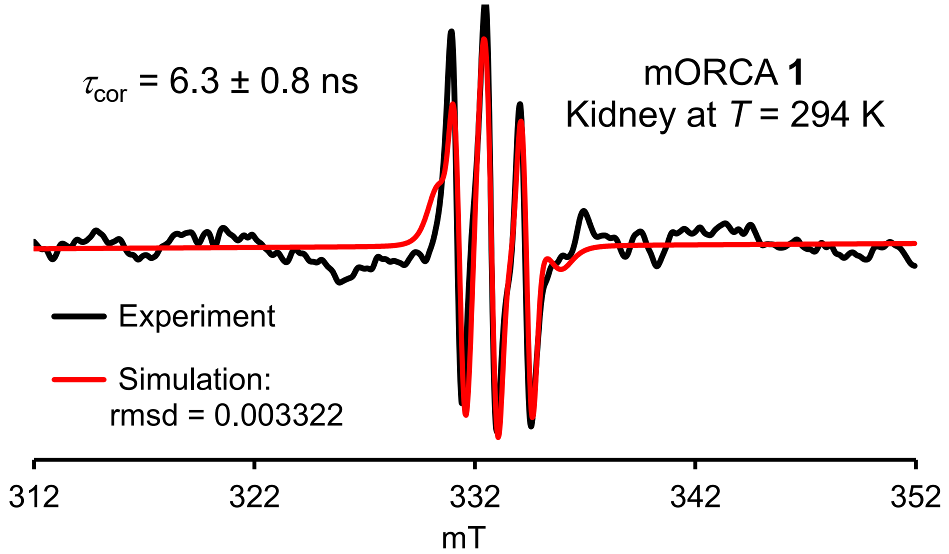

Fig. S56. Ex vivo EPR spectrum of the homogenized kidney (diluted with PBS) at 294 K (mouse label: SD130M2; EPR label: SD132R38). A 2 mm OD EPR quartz tube is employed. Spectral simulations are carried out with the EasySpin *chili* suite; key simulation parameters are summarized in Table S5.

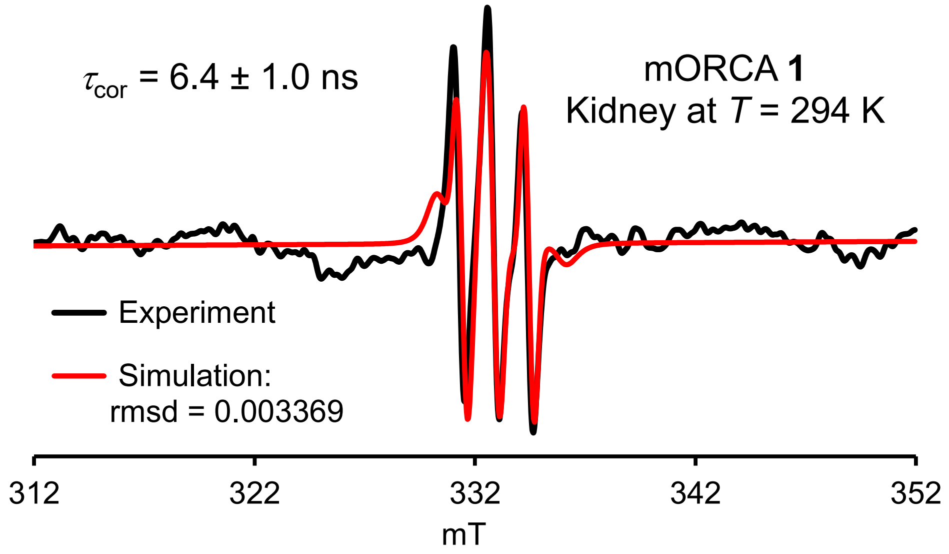

Fig. S57. Ex vivo EPR spectrum of the homogenized kidney (diluted with PBS) at 294 K (mouse label: SD130M2; EPR label: SD132R40). A 2 mm OD EPR quartz tube is employed. Spectral simulations are carried out with the EasySpin *chili* suite; key simulation parameters are summarized in Table S5.

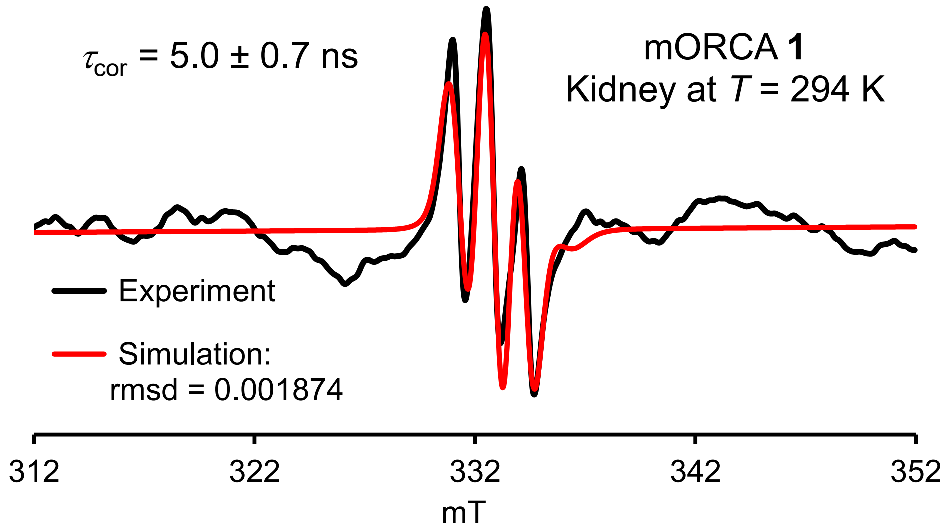

Fig. S58. Ex vivo EPR spectrum of the homogenized kidney (diluted with PBS) at 294 K (mouse label: SD130M2; EPR label: SD132R41). A 2 mm OD EPR quartz tube is employed. Spectral simulations are carried out with the EasySpin *chili* suite; key simulation parameters are summarized in Table S5.

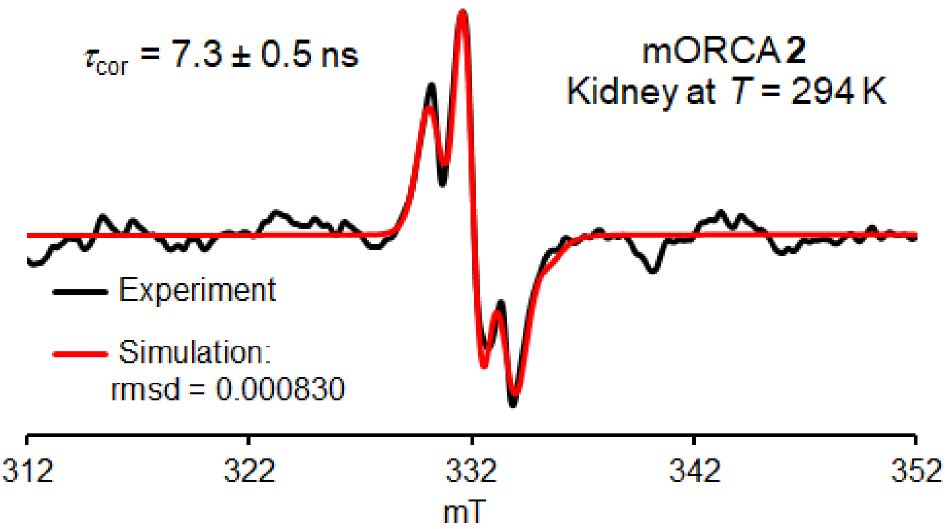

Fig. S59. This figure is identical to that in the main text (Figure 5E). Ex vivo EPR spectrum of the homogenized kidney (diluted with PBS) at 294 K (mouse label: SZ530M2; EPR label: SZ531R02). A 0.6 mm ID EPR quartz capillary is employed. Spectral simulations are carried out with the EasySpin *chili* suite; key simulation parameters are summarized in Table S5.

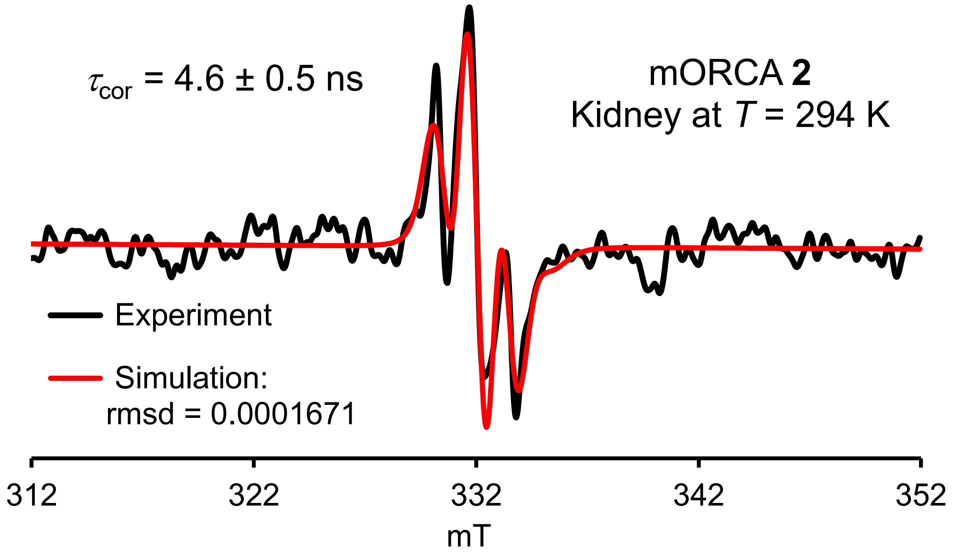

Fig. S60. Ex vivo EPR spectrum of the homogenized kidney (diluted with PBS) at 294 K (mouse label: SZ530M2; EPR label: SZ531R03). A 0.6 mm ID EPR quartz capillary is employed. Spectral simulations are carried out with the EasySpin *chili* suite; key simulation parameters are summarized in Table S5.

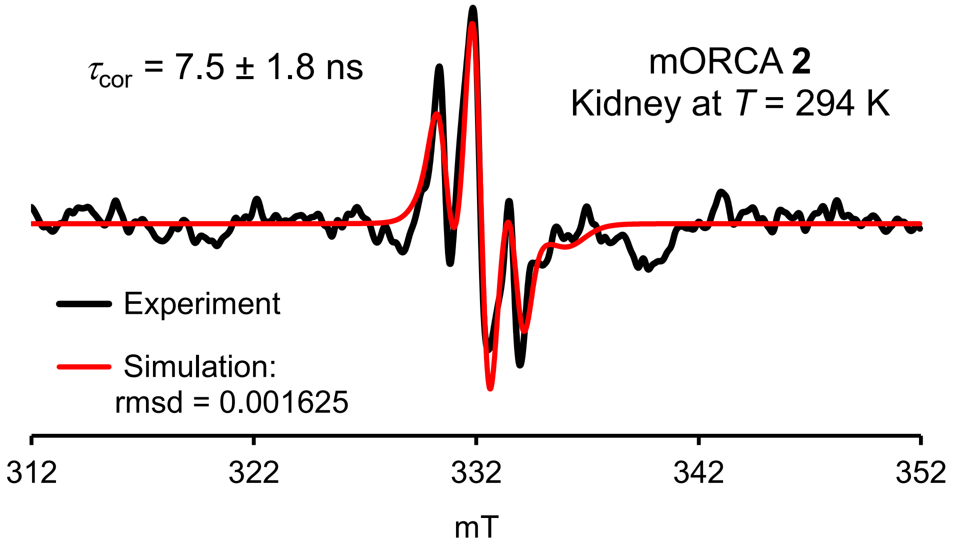

Fig. S61. Ex vivo EPR spectrum of the homogenized kidney (diluted with PBS) at 294 K (mouse label: SZ540M2; EPR label: SZ550R09). Spectral simulations are carried out with the EasySpin *chili* suite; key simulation parameters are summarized in Table S5.

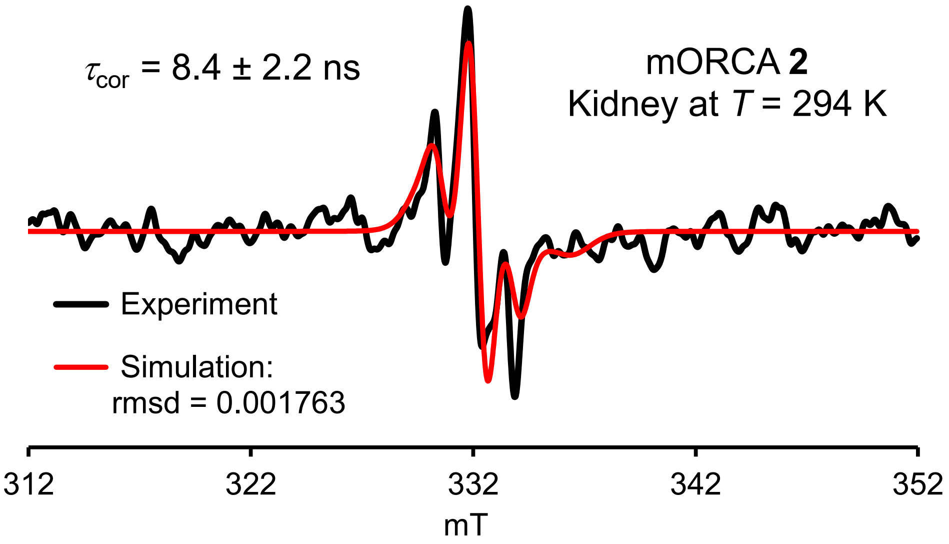

Fig. S62. Ex vivo EPR spectrum of the homogenized kidney (diluted with PBS) at 294 K (mouse label: SZ540M2; EPR label: SZ550R11). Spectral simulations are carried out with the EasySpin *chili* suite; key simulation parameters are summarized in Table S5.

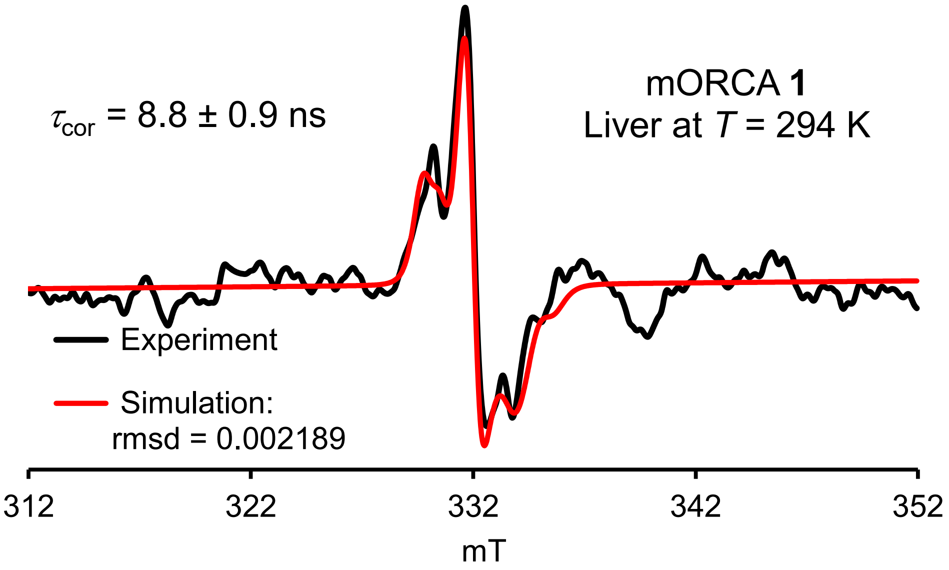

Fig. S63. Ex vivo EPR spectrum of the homogenized liver (diluted with PBS) at 294 K (mouse label: SD129M1; EPR label: SD130R13). A 0.6 mm ID EPR quartz capillary is employed. Spectral simulations are carried out with the EasySpin *chili* suite; key simulation parameters are summarized in Table S5.

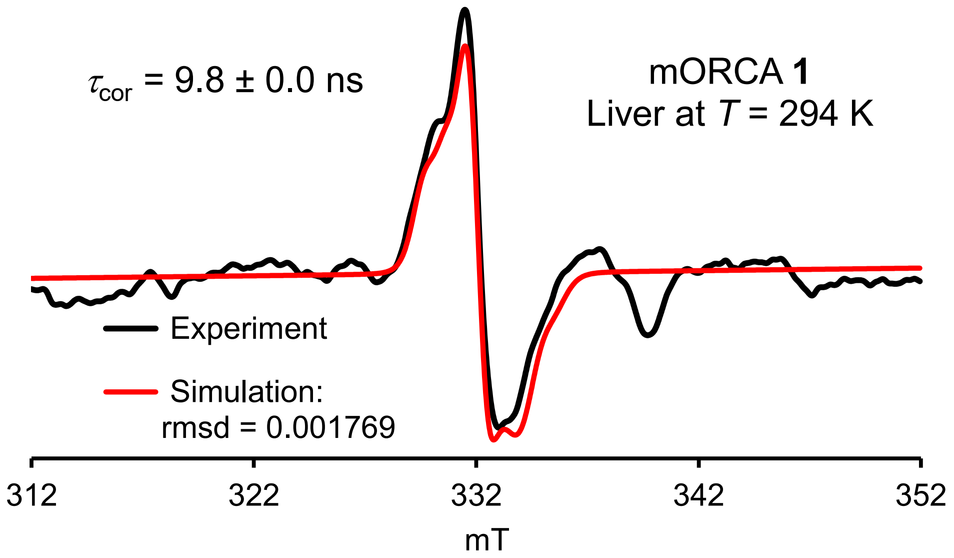

Fig. S64. Ex vivo EPR spectrum of the homogenized liver (diluted with PBS) at 294 K (mouse label: SD129M1; EPR label: SD130R14). A 0.6 mm ID EPR quartz capillary is employed. Spectral simulations are carried out with the EasySpin *chili* suite; key simulation parameters are summarized in Table S5.

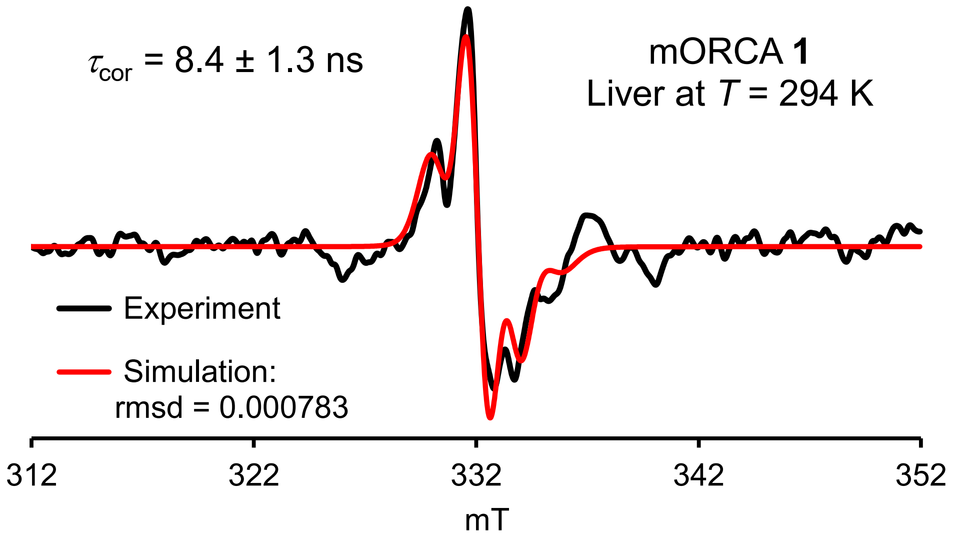

Fig. S65. Ex vivo EPR spectrum of the homogenized liver (diluted with PBS) at 294 K (mouse label: SD130M2; EPR label: SD132R11). A 0.6 mm ID EPR quartz capillary is employed. Spectral simulations are carried out with the EasySpin *chili* suite; key simulation parameters are summarized in Table S5.

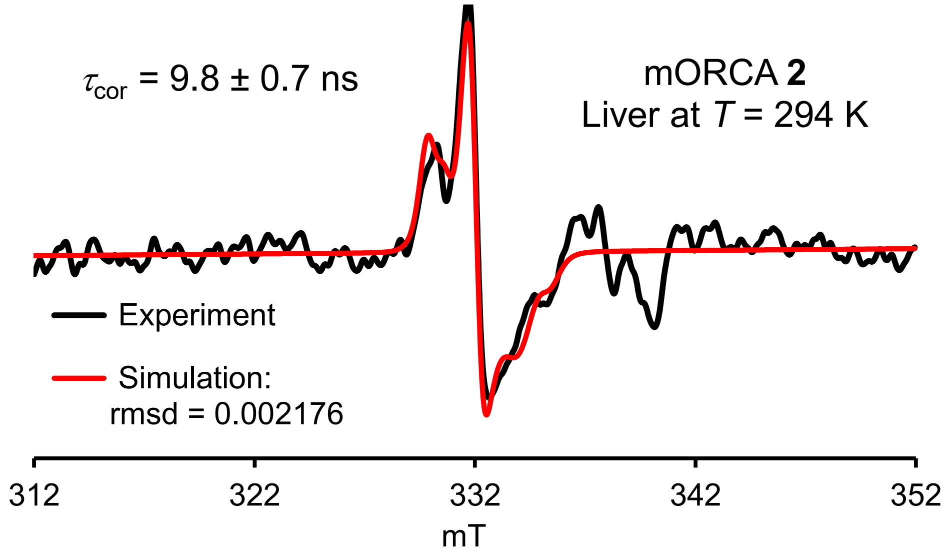

Fig. S66. Ex vivo EPR spectrum of the homogenized liver (diluted with PBS) at 294 K (mouse label: SZ530M2; EPR label: SZ531R22). A 0.6 mm ID EPR quartz capillary is employed. Spectral simulations are carried out with the EasySpin *chili* suite; key simulation parameters are summarized in Table S5.

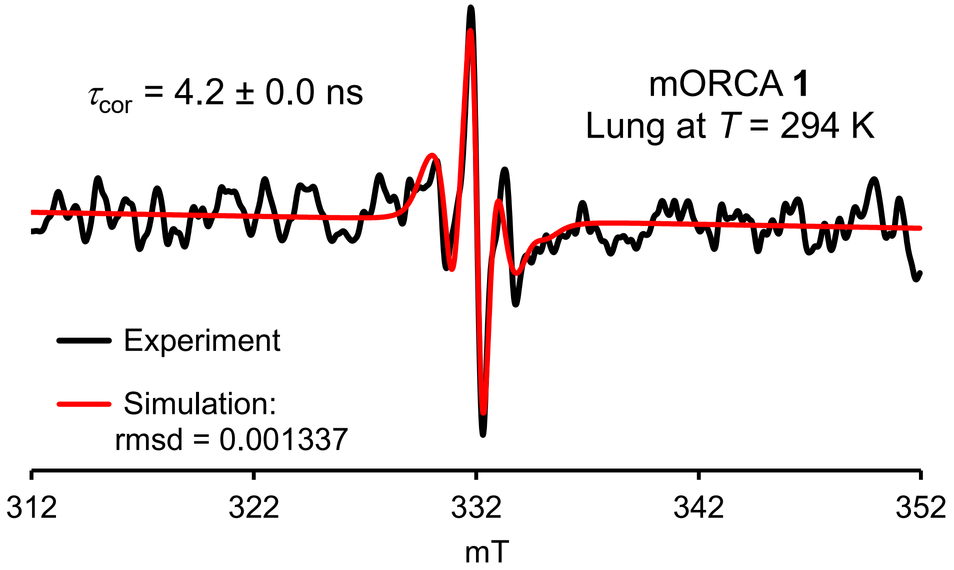

Fig. S67. Ex vivo EPR spectrum of the homogenized lung (diluted with PBS) at 294 K (mouse label: SD129M1; EPR label: SD130R07). A 0.6 mm ID EPR quartz capillary is employed. Spectral simulations are carried out with the EasySpin *chili* suite; key simulation parameters are summarized in Table S5.

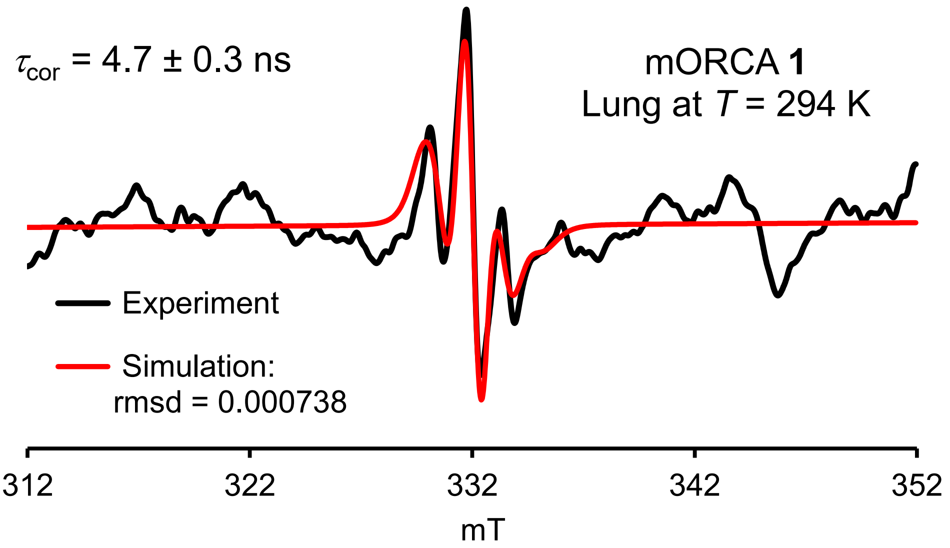

Fig. S68. Ex vivo EPR spectrum of the homogenized lung (diluted with PBS) at 294 K (mouse label: SD129M1; EPR label: SD130R08). A 0.6 mm ID EPR quartz capillary is employed. Spectral simulations are carried out with the EasySpin *chili* suite; key simulation parameters are summarized in Table S5.

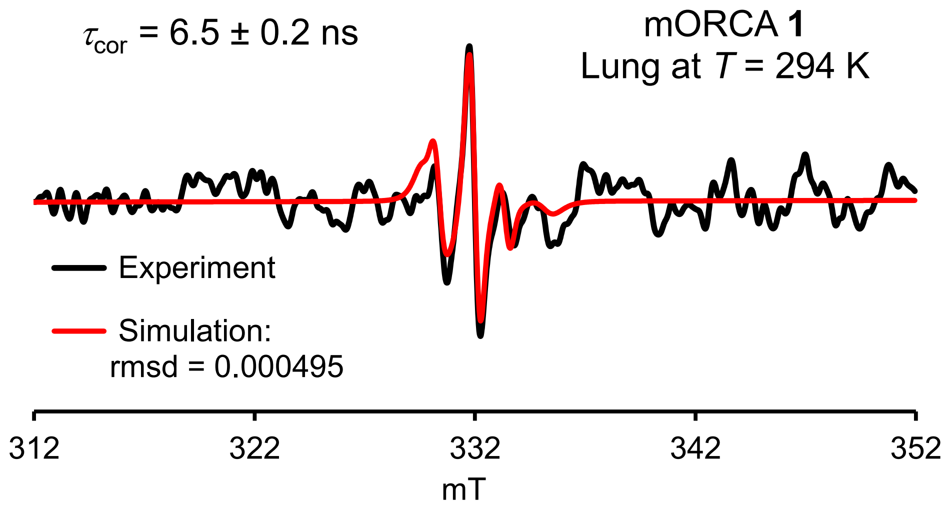

Fig. S69. Ex vivo EPR spectrum of the homogenized lung (diluted with PBS) at 294 K (mouse label: SD130M2, EPR label: SD132R05). A 0.6 mm ID EPR quartz capillary is employed. Spectral simulations are carried out with the EasySpin *chili* suite; key simulation parameters are summarized in Table S5.

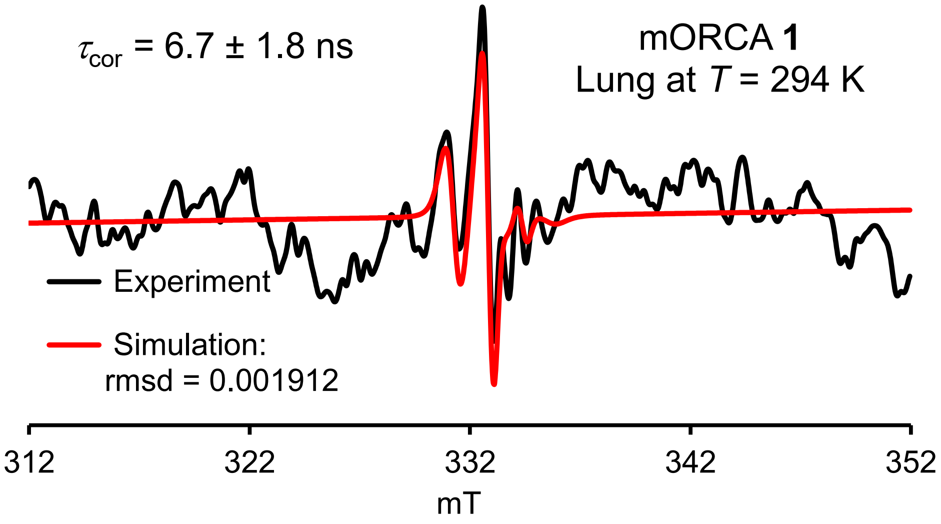

Fig. S70. Ex vivo EPR spectrum of the homogenized lung (diluted with PBS) at 294 K (mouse label: SD130M2, EPR label: SD132R44). A 2 mm ID EPR quartz tube is employed. Spectral simulations are carried out with the EasySpin *chili* suite; key simulation parameters are summarized in Table S5.

5. Characterization of 1, 2, 2a, and selected intermediates via ^{1}H and ^{13}C NMR, IR spectroscopies, and mass spectrometry (MS).

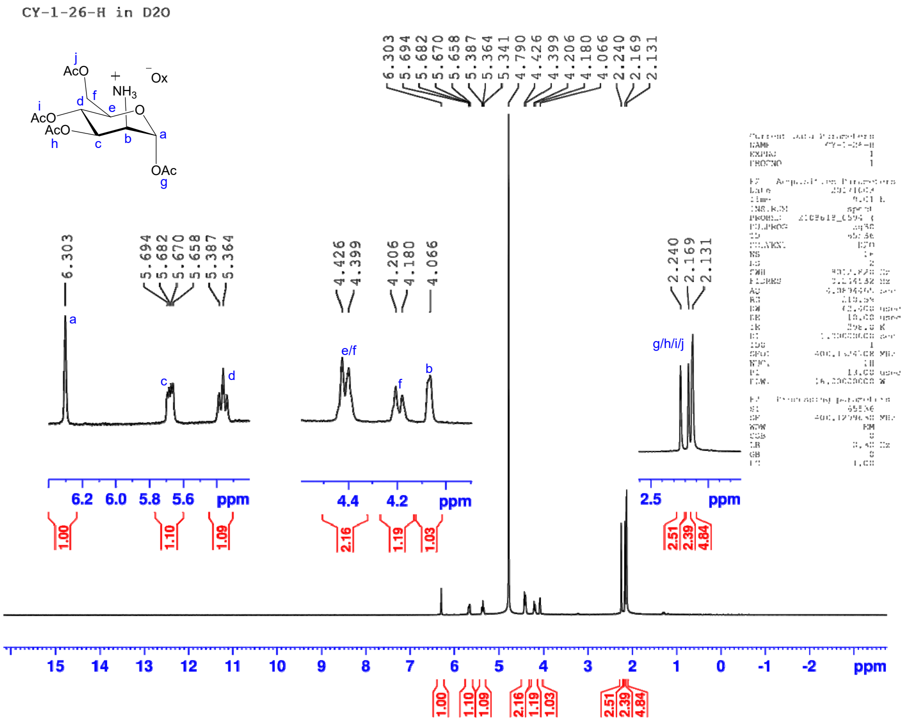

Figure S71. ^{1}H NMR of compound **5** (sample label: CY-1-26-H, 400 MHz, and D_2O).

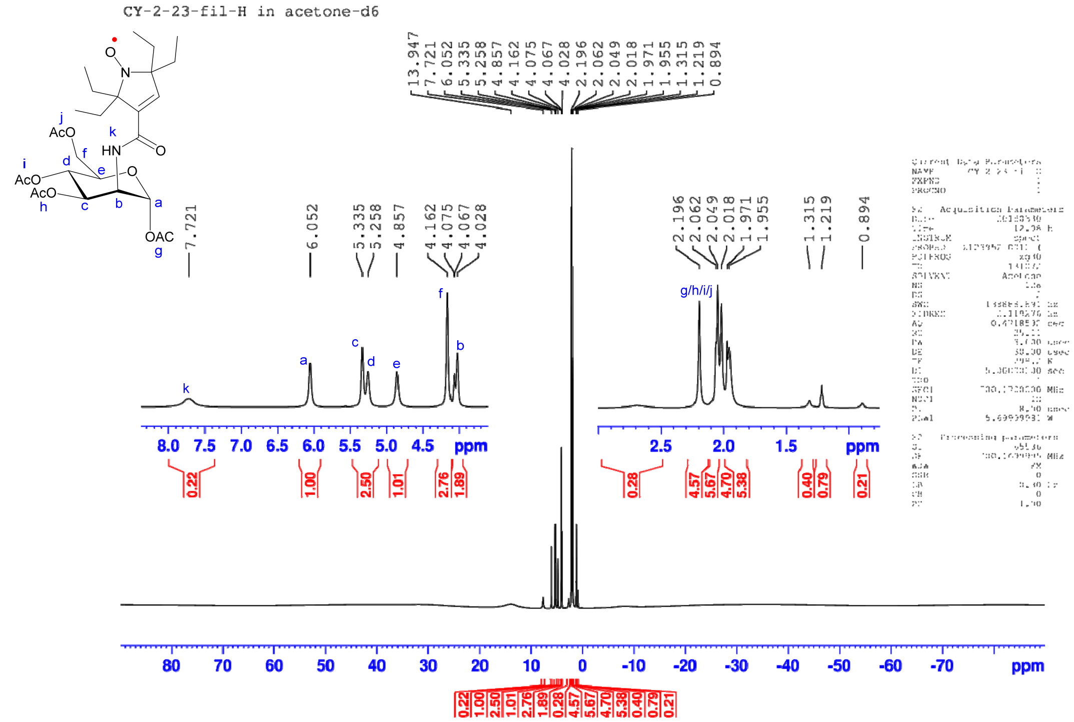

Figure S72. ^{1}H NMR of compound **2** (sample label: CY-2-23-fil-H, 700 MHz, and 0.1756 M in acetone-*d*6).

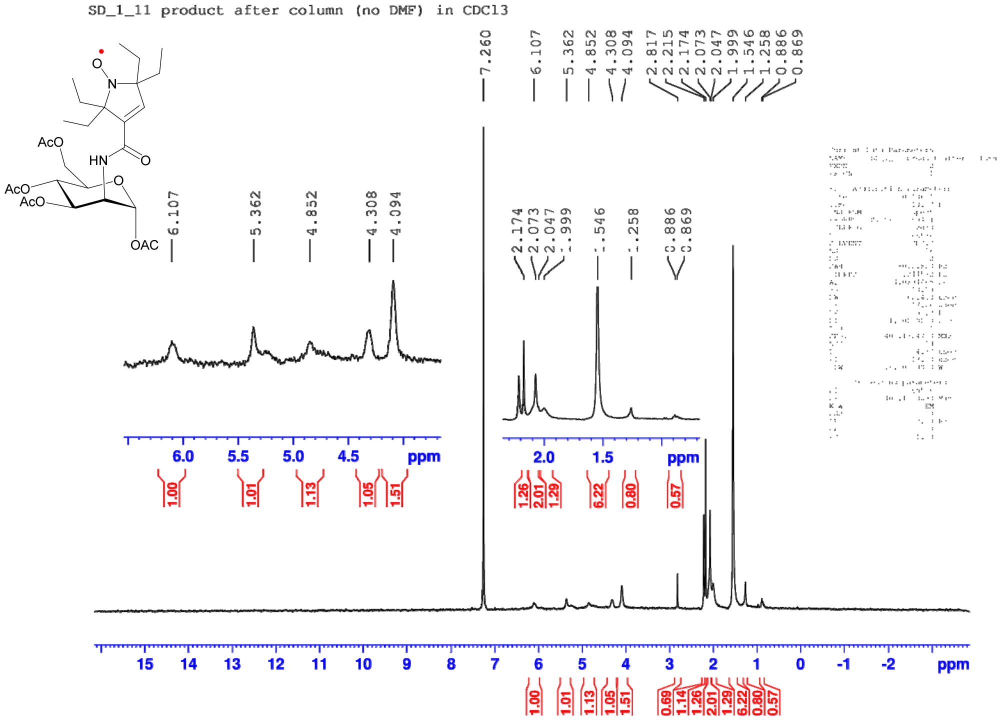

Figure S73. ^{1}H NMR of compound **2** (sample label: SD-1-11-product, $CDCl_3$, 400 MHz, and <0.01 M in $CDCl_3$).

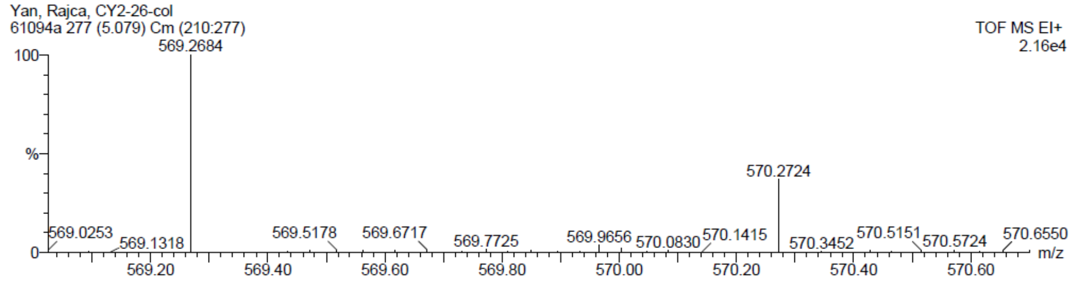

Mass	RA	Calc. Mass	mDa	PPM	DBE	Score	Formula
569.2684	100.00	569.2666	1.8	3.2	9.0	n/a	12C26 13C 1H40 14N2 16O11
		569.2710	-2.6	-4.6	8.5	n/a	12C27 1H41 14N2 16O11
		569.3030	-34.6	-60.7	8.0	n/a	12C27 13C 1H44 14N2 16O10
		569.3074	-39.0	-68.5	7.5	n/a	12C28 1H45 14N2 16O10
		569.2135	54.9	96.4	14.5	n/a	12C29 1H33 14N2 16O10
		569.2091	59.3	104.3	15.0	n/a	12C28 13C 1H32 14N2 16O10
		569.1771	91.3	160.3	15.5	n/a	12C28 1H29 14N2 16O11
		569.1727	95.7	168.2	16.0	n/a	12C27 13C 1H28 14N2 16O11
		569.1196	148.8	261.4	21.5	n/a	12C30 1H21 14N2 16O10
		569.1152	153.2	269.2	22.0	n/a	12C29 13C 1H20 14N2 16O10
		569.0832	185.2	325.3	22.5	n/a	12C29 1H17 14N2 16O11
		569.0788	189.6	333.1	23.0	n/a	12C28 13C 1H16 14N2 16O11
		569.0257	242.7	426.3	28.5	n/a	12C31 1H9 14N2 16O10
		569.0212	247.2	434.2	29.0	n/a	12C30 13C 1H8 14N2 16O10
		568.9893	279.1	490.2	29.5	n/a	12C30 1H5 14N2 16O11
		568.9849	283.5	498.1	30.0	n/a	12C29 13C 1H4 14N2 16O11
570.2724	36.82	570.2744	-2.0	-3.5	8.5	n/a	12C26 13C 1H41 14N2 16O11
		570.2789	-6.5	-11.3	8.0	n/a	12C27 1H42 14N2 16O11
		570.3108	-38.4	-67.3	7.5	n/a	12C27 13C 1H45 14N2 16O10
		570.3152	-42.8	-75.1	7.0	n/a	12C28 1H46 14N2 16O10
		570.2213	51.1	89.5	14.0	n/a	12C29 1H34 14N2 16O10
		570.2169	55.5	97.4	14.5	n/a	12C28 13C 1H33 14N2 16O10
		570.1850	87.4	153.3	15.0	n/a	12C28 1H30 14N2 16O11
		570.1805	91.9	161.2	15.5	n/a	12C27 13C 1H29 14N2 16O11
		570.1274	145.0	254.2	21.0	n/a	12C30 1H22 14N2 16O10
		570.1230	149.4	262.0	21.5	n/a	12C29 13C 1H21 14N2 16O10
		570.0911	181.3	318.0	22.0	n/a	12C29 1H18 14N2 16O11
		570.0866	185.8	325.8	22.5	n/a	12C28 13C 1H17 14N2 16O11
		570.0335	238.9	418.8	28.0	n/a	12C31 1H10 14N2 16O10
		570.0291	243.3	426.7	28.5	n/a	12C30 13C 1H9 14N2 16O10
		569.9972	275.2	482.6	29.0	n/a	12C30 1H6 14N2 16O11
		569.9927	279.7	490.5	29.5	n/a	12C29 13C 1H5 14N2 16O11

Figure S74. HR-MS-EI (m/z) for compound **2** (sample label: CY-2-26-col): $[M]^+$ calculated for $C_{27}H_{41}N_2O_{11}$ 569.2710 was found to be 569.2684 (-4.6 PPM, RA 100%); $[M+H]^+$ calculated for $C_{27}H_{42}N_2O_{11}$ 570.2789 was found to be 570.2724 (-11.3 PPM, RA 37%).

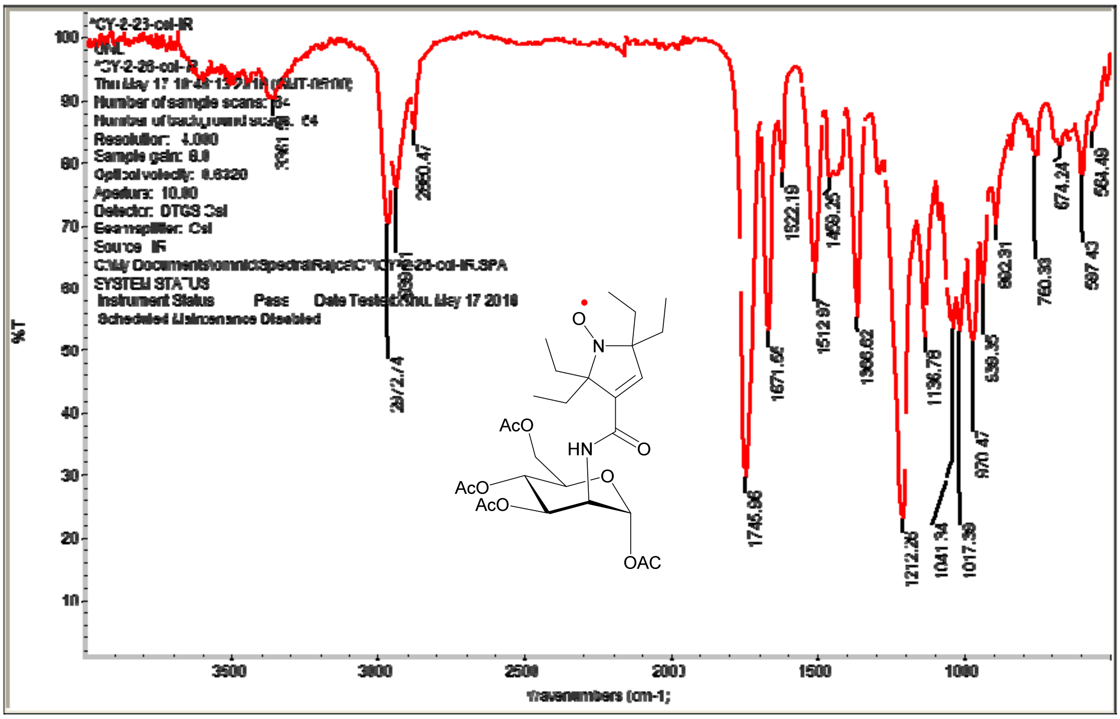

Figure S75. IR spectrum for compound **2** (sample label:CY-2-26-col-IR).

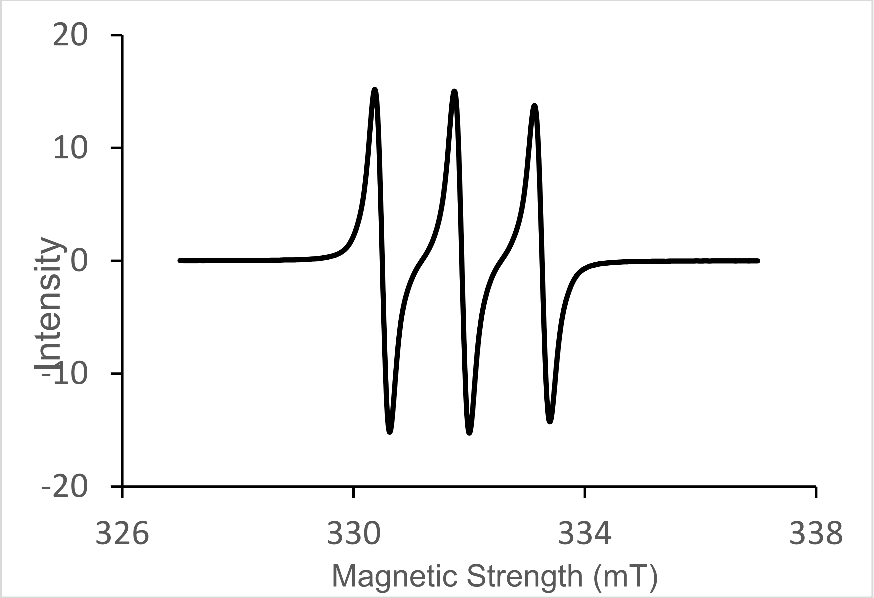

Figure S76. EPR spectrum of compound **2** (sample label: SZ467, 0.922 mM in chloroform/toluene (1:4), frequency = 9.31994 GHz, modulation amplitude = 0.1 mT, attenuation = 30 dB, receiver gain = 30 dB, central field = 332 mT, sweep width = 10 mT, sweep time = 5.24 s, and number of scans = 2).

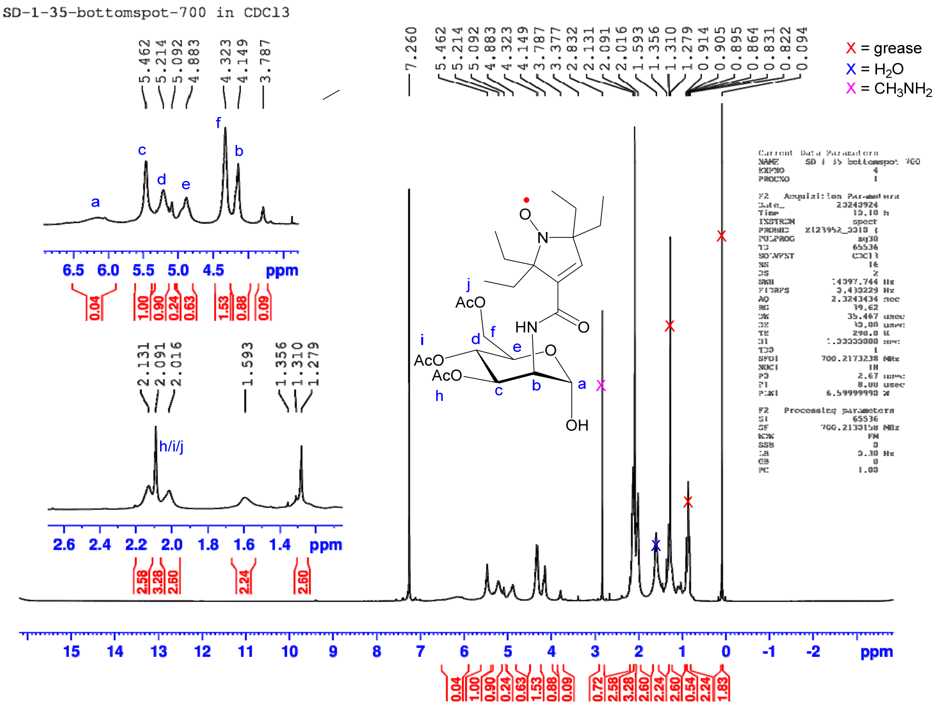

Figure S77. ^{1}H NMR of compound **3** (sample label: SD-1-35-bottomspot, 700 MHz, and 0.027 M in $CDCl_3$). Note: the ^{1}H singlet at 2.83 ppm is likely to be due to methylamine used as the reagent.[S5]

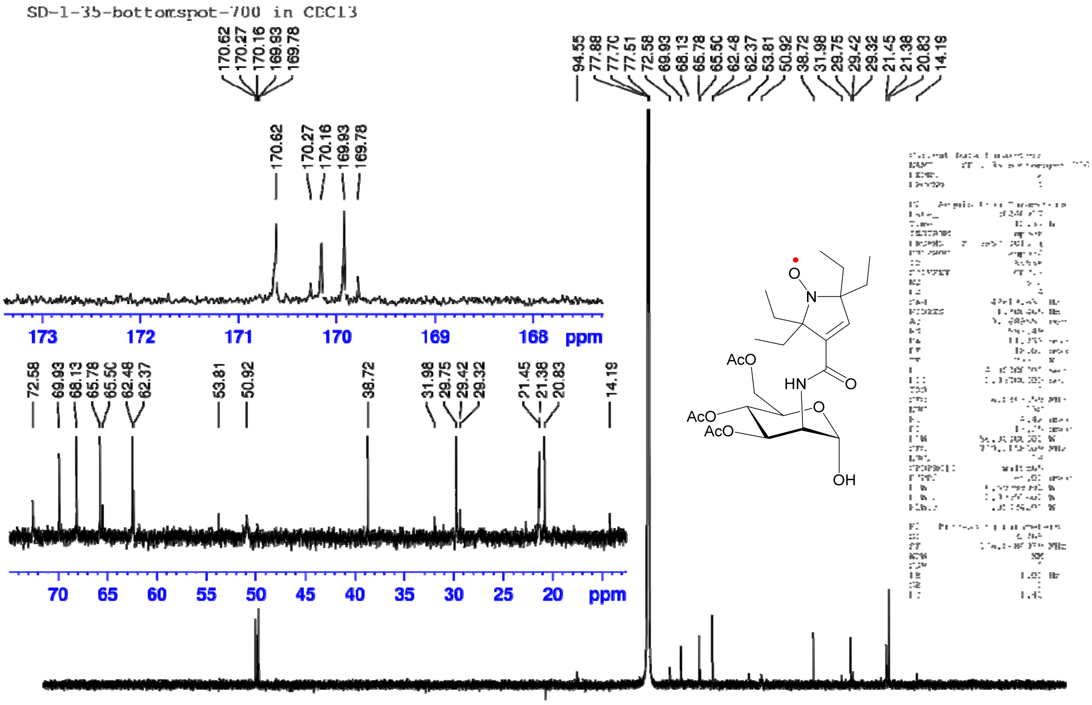

Figure S78. ^{13}C NMR of compound **3** (sample label: SD-1-35-bottomspot, 700 MHz, and 0.027 M in $CDCl_3$).

Multiple Mass Analysis: 2 mass(es) processed
Tolerance = 5.0 PPM / DBE: min = -1.5, max = 50.0
Element prediction: Off
Number of isotope peaks used for i-FIT = 3

Monoisotopic Mass, Odd and Even Electron Ions
1310642 formula(e) evaluated with 27 results within limits (up to 50 best isotopic matches for each mass)
Elements Used:
12C: 0-50 13C: 0-2 1H: 0-100 14N: 0-5 16O: 0-12 23Na: 1-1
61968 SD-1-40-BOTTOM 584 (1.244) AM (Med,5, Ar,10000.0,0.00,0.70); Cm (409:1083)
TOF MS ES+

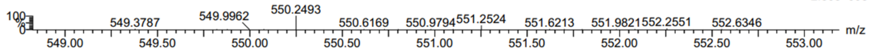

Mass	RA	Calc. Mass	mDa	PPM	DBE	i-FIT	Norm	Conf(%)	Formula
Minimum:	10.00				-1.5				
Maximum:	100.00		5.0	5.0	50.0				
550.2493	100.00	550.2493	0.0	0.0	21.5	-1.$	n/a	1.#R	12C32 13C2 1H31 14N5 16O 23Na
		550.2489	0.4	0.7	7.5	-1.$	n/a	1.#R	12C23 1H37 14N5 16O9 23Na
		550.2498	-0.5	-0.9	11.5	-1.$	n/a	1.#R	12C29 13C 1H38 16O8 23Na
		550.2499	-0.6	-1.1	3.5	-1.$	n/a	1.#R	12C19 13C2 1H39 14N3 16O12 23Na
		550.2485	0.8	1.5	12.0	-1.$	n/a	1.#R	12C27 13C 1H36 14N3 16O7 23Na
		550.2484	0.9	1.6	20.0	-1.$	n/a	1.#R	12C37 1H35 16O3 23Na
		550.2502	-0.9	-1.6	7.0	-1.$	n/a	1.#R	12C25 1H39 14N2 16O10 23Na
		550.2480	1.3	2.4	16.5	-1.$	n/a	1.#R	12C31 13C2 1H35 14N 16O5 23Na
		550.2507	-1.4	-2.5	21.0	-1.$	n/a	1.#R	12C34 13C2 1H33 14N2 16O2 23Na
		550.2511	-1.8	-3.3	24.5	-1.$	n/a	1.#R	12C40 1H33 14N 23Na
		550.2511	-1.8	-3.3	16.5	-1.$	n/a	1.#R	12C30 13C 1H34 14N4 16O4 23Na
		550.2470	2.3	4.2	20.5	-1.$	n/a	1.#R	12C35 1H33 14N3 16O2 23Na
		550.2467	2.6	4.7	17.0	-1.$	n/a	1.#R	12C29 13C2 1H33 14N4 16O4 23Na
		550.2466	2.7	4.9	25.0	-1.$	n/a	1.#R	12C39 13C 1H32 14N 23Na
551.2524	27.34	551.2523	0.1	0.2	7.5	-1.$	n/a	1.#R	12C22 13C 1H37 14N5 16O9 23Na
		551.2522	0.2	0.4	15.5	-1.$	n/a	1.#R	12C32 1H36 14N2 16O5 23Na
		551.2518	0.6	1.1	12.0	-1.$	n/a	1.#R	12C26 13C2 1H36 14N3 16O7 23Na
		551.2531	-0.7	-1.3	11.5	-1.$	n/a	1.#R	12C28 13C2 1H38 16O8 23Na
		551.2517	0.7	1.3	20.0	-1.$	n/a	1.#R	12C36 13C 1H35 16O3 23Na
		551.2536	-1.2	-2.2	7.0	-1.$	n/a	1.#R	12C24 13C 1H39 14N2 16O10 23Na
		551.2508	1.6	2.9	16.0	-1.$	n/a	1.#R	12C30 1H34 14N5 16O4 23Na
		551.2540	-1.6	-2.9	2.5	-1.$	n/a	1.#R	12C20 1H40 14N4 16O12 23Na
		551.2544	-2.0	-3.6	24.5	-1.$	n/a	1.#R	12C39 13C 1H33 14N 23Na
		551.2504	2.0	3.6	20.5	-1.$	n/a	1.#R	12C34 13C 1H33 14N3 16O2 23Na
		551.2545	-2.1	-3.8	16.5	-1.$	n/a	1.#R	12C29 13C2 1H34 14N4 16O4 23Na
		551.2500	2.4	4.4	25.0	-1.$	n/a	1.#R	12C38 13C2 1H32 14N 23Na
		551.2549	-2.5	-4.5	20.0	-1.$	n/a	1.#R	12C35 1H34 14N3 16O2 23Na

Figure S79. HR-MS-ES (m/z) for compound **3** (label: SD-1-40-bottom): $[M+Na]^+$ calculated for $C_{25}H_{39}N_2O_{10}Na$ 550.2502 was found to be 550.2493 (-1.6 ppm, RA 100%), and for $^{12}C_{24}{}^{13}CH_{39}N_2O_{10}Na$ 551.2536, it was found to be 551.2524 (-2.2 PPM, RA 27%).

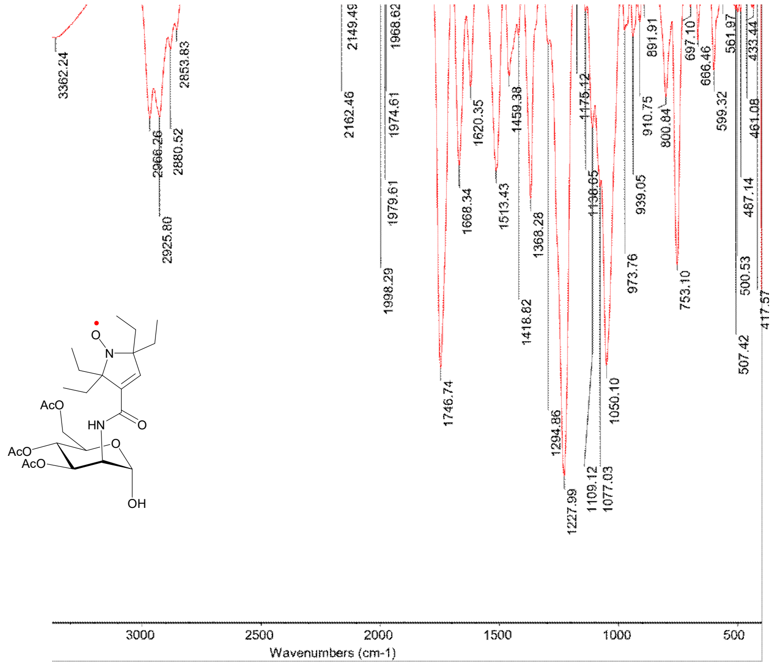

Figure S80. IR spectrum for compound **3** (sample label: SD-1-35-bottomspot).

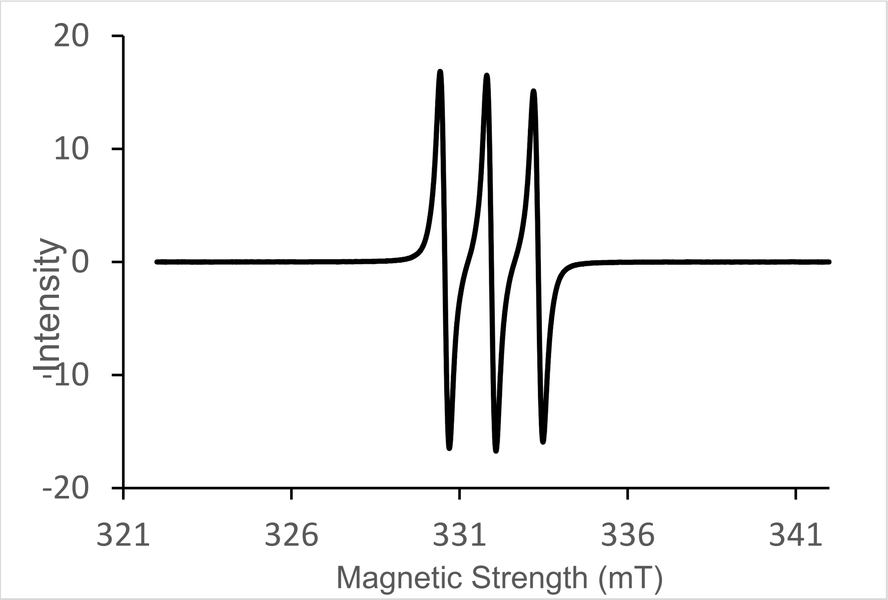

Figure S81. EPR spectrum of compound **3** (sample label: SD-1-40-bottom, 1.10 mM in chloroform/toluene (1:4), frequency = 9.321122 GHz, modulation amplitude = 0.1 mT, attenuation = 30 dB, receiver gain = 35 dB, center field = 332 mT, sweep width = 20 mT, sweep time = 10 s, and number of scans = 2).

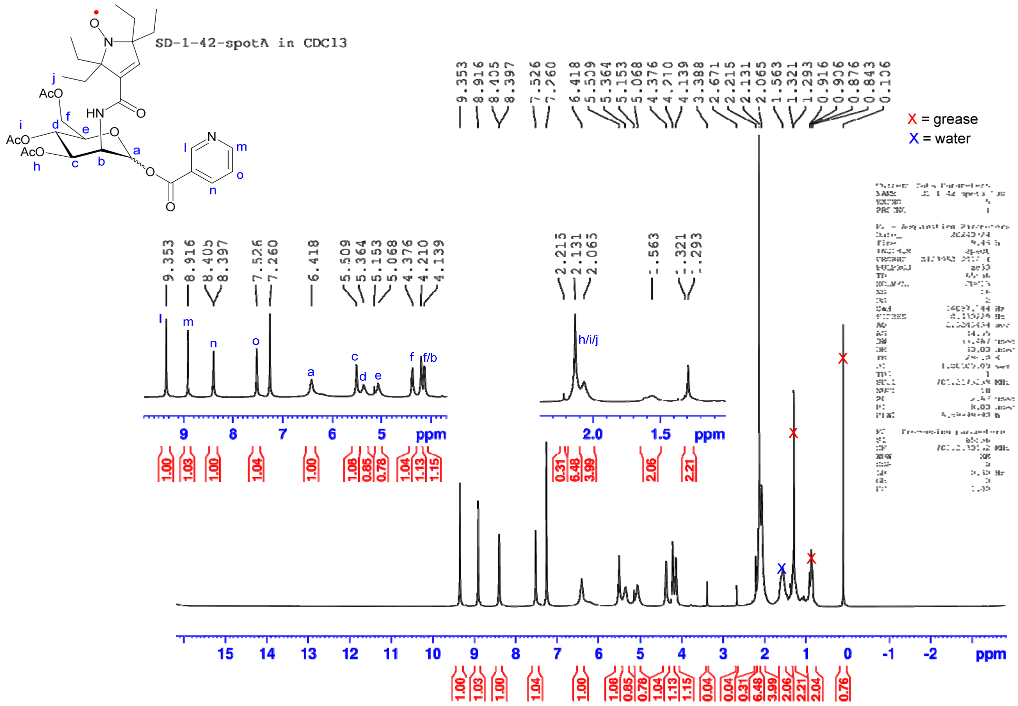

Figure S82. ^{1}H NMR of compound **1** (sample label: SD-1-42-spotA, 700 MHz, and 0.041 M in $CDCl_3$).

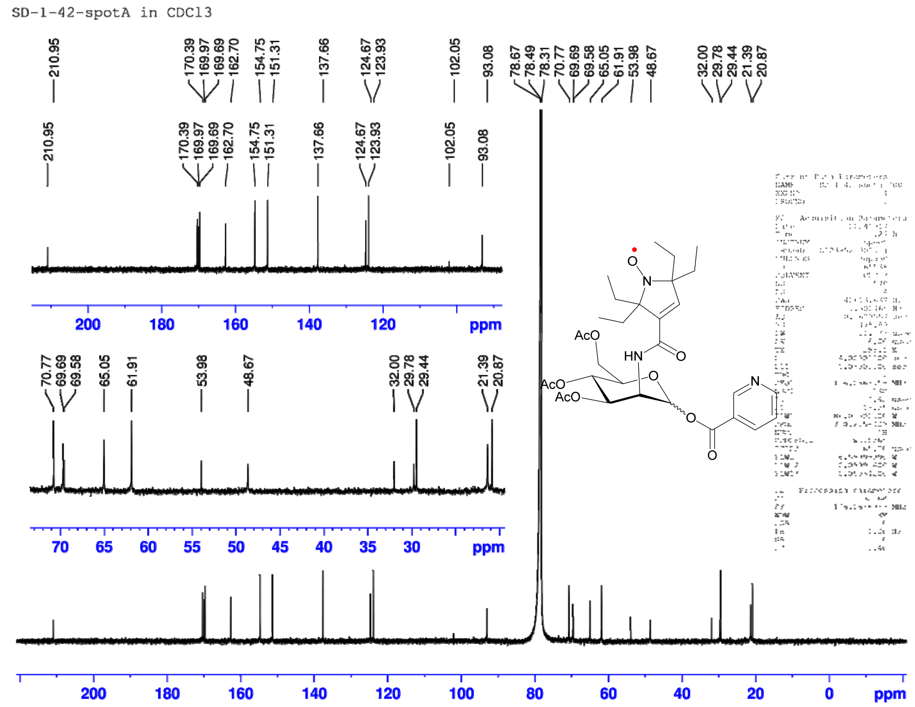

Figure S83. ^{13}C NMR of compound **1** (sample label: SD-1-42-spotA, 700 MHz, and 0.041 M in $CDCl_3$).

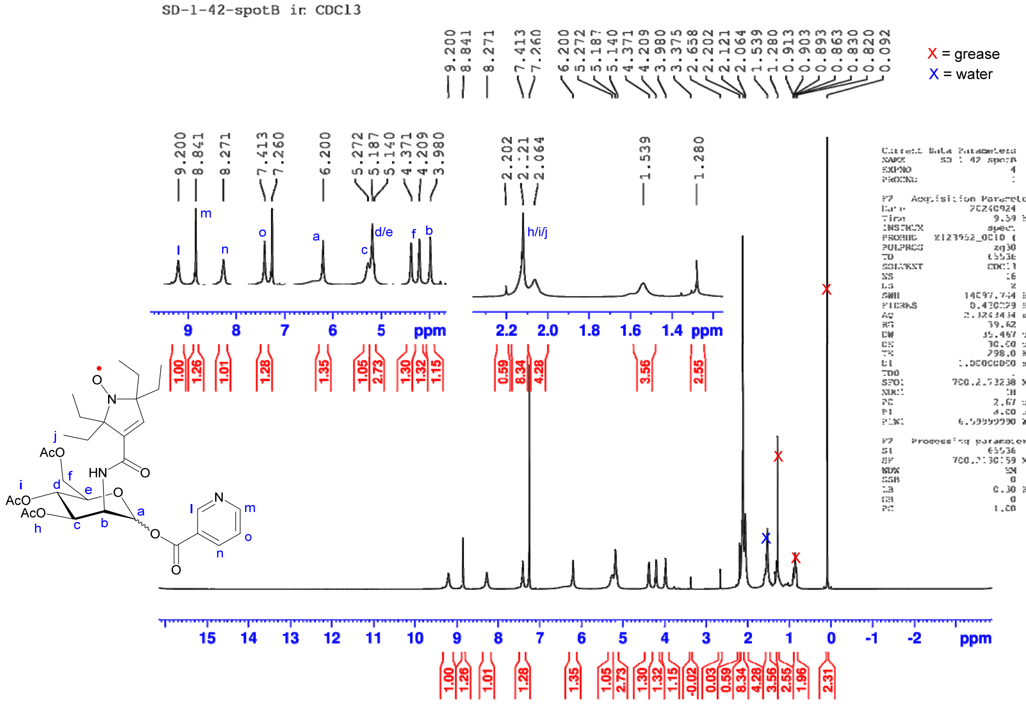

Figure S84. ^{1}H NMR of compound **1** (sample label: SD-1-42-spotB, 700 MHz, and 0.053 M in $CDCl_3$).

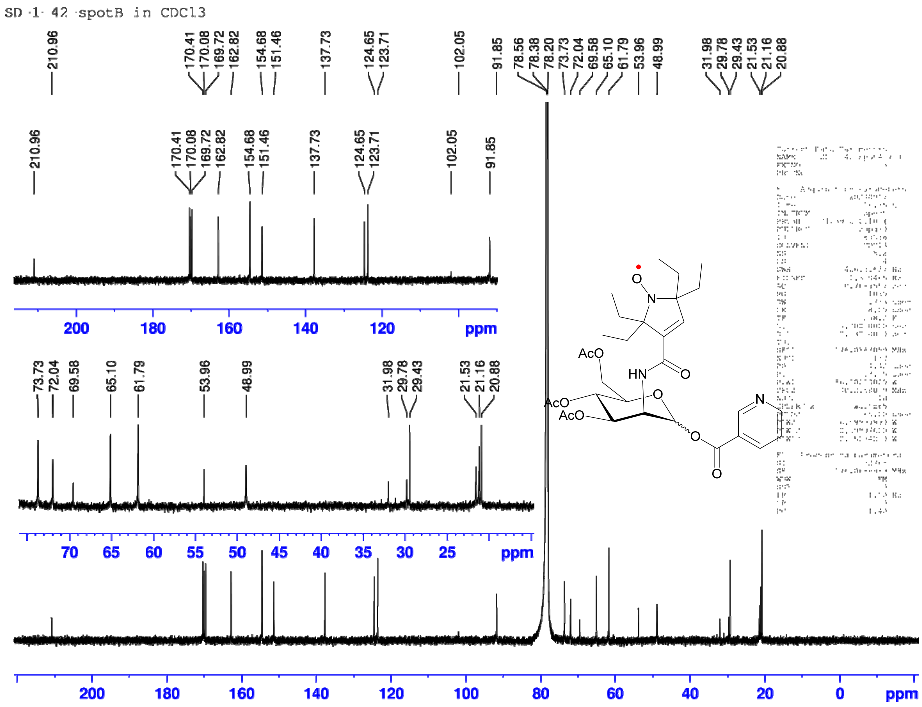

Figure S85. ^{13}C NMR of compound **1** (sample label: SD-1-42-spotB, 700 MHz, and 0.053 M in $CDCl_3$).

Multiple Mass Analysis: 3 mass(es) processed
Tolerance = 5.0 PPM / DBE: min = -1.5, max = 50.0
Element prediction: Off
Number of isotope peaks used for i-FIT = 3

Monoisotopic Mass, Odd and Even Electron Ions
2589452 formula(e) evaluated with 51 results within limits (up to 50 best isotopic matches for each mass)
Elements Used:
12C: 0-50 13C: 0-2 1H: 0-100 14N: 0-5 16O: 0-12 23Na: 1-1
61969 SD-1-42SPOT3 658 (1.437) AM (Med,5, Ar,10000.0,0.00,0.70); Cm (201:730)
TOF MS ES+

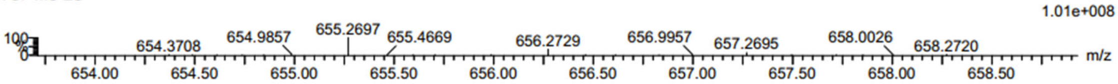

Mass	RA	Calc. Mass	mDa	PPM	DBE	i-FIT	Norm	Conf(%)	Formula
Minimum:	10.00				-1.5				
Maximum:	100.00		5.0	5.0	50.0				
655.2697	100.00	655.2695	0.2	0.3	21.5	-1.$	n/a	1.#R	12C37 13C2 1H38 14N2 16O6 23Na
		655.2699	-0.2	-0.3	25.0	-1.$	n/a	1.#R	12C43 1H38 14N 16O4 23Na
		655.2699	-0.2	-0.3	17.0	-1.$	n/a	1.#R	12C33 13C 1H39 14N4 16O8 23Na
		655.2686	1.1	1.7	12.0	-1.$	n/a	1.#R	12C32 13C 1H43 16O12 23Na
		655.2685	1.2	1.8	25.5	-1.$	n/a	1.#R	12C41 1H36 14N4 16O3 23Na
		655.2712	-1.5	-2.3	30.0	-1.$	n/a	1.#R	12C44 1H34 14N5 23Na
		655.2713	-1.6	-2.4	16.5	-1.$	n/a	1.#R	12C35 13C 1H41 14N 16O9 23Na
		655.2681	1.6	2.4	30.0	-1.$	n/a	1.#R	12C45 13C 1H35 14N2 16O 23Na
		655.2681	1.6	2.4	22.0	-1.$	n/a	1.#R	12C35 13C2 1H36 14N5 16O5 23Na
		655.2717	-2.0	-3.1	12.0	-1.$	n/a	1.#R	12C31 1H42 14N3 16O11 23Na
		655.2721	-2.4	-3.7	26.0	-1.$	n/a	1.#R	12C40 13C2 1H36 14N3 16O3 23Na
		655.2672	2.5	3.8	12.5	-1.$	n/a	1.#R	12C30 13C 1H41 14N3 16O11 23Na
		655.2672	2.5	3.8	20.5	-1.$	n/a	1.#R	12C40 1H40 16O7 23Na
		655.2725	-2.8	-4.3	29.5	-1.$	n/a	1.#R	12C46 1H36 14N2 16O 23Na
		655.2668	2.9	4.4	17.0	-1.$	n/a	1.#R	12C34 13C2 1H40 14N 16O9 23Na
		655.2726	-2.9	-4.4	21.5	-1.$	n/a	1.#R	12C36 13C 1H37 14N5 16O5 23Na
		655.2667	3.0	4.6	30.5	-1.$	n/a	1.#R	12C43 13C 1H33 14N5 23Na
656.2729	33.17	656.2732	-0.3	-0.5	25.0	-1.$	n/a	1.#R	12C42 13C 1H38 14N 16O4 23Na
		656.2733	-0.4	-0.6	17.0	-1.$	n/a	1.#R	12C32 13C2 1H39 14N4 16O8 23Na
		656.2737	-0.8	-1.2	20.5	-1.$	n/a	1.#R	12C38 1H39 14N3 16O6 23Na
		656.2719	1.0	1.5	12.0	-1.$	n/a	1.#R	12C31 13C2 1H43 16O12 23Na
		656.2719	1.0	1.5	25.5	-1.$	n/a	1.#R	12C40 13C 1H36 14N4 16O3 23Na
		656.2714	1.5	2.3	30.0	-1.$	n/a	1.#R	12C44 13C2 1H35 14N2 16O 23Na
		656.2745	-1.6	-2.4	30.0	-1.$	n/a	1.#R	12C43 13C 1H34 14N5 23Na
		656.2746	-1.7	-2.6	16.5	-1.$	n/a	1.#R	12C34 13C2 1H41 14N 16O9 23Na
		656.2710	1.9	2.9	16.0	-1.$	n/a	1.#R	12C35 1H41 14N2 16O9 23Na
		656.2750	-2.1	-3.2	20.0	-1.$	n/a	1.#R	12C40 1H41 16O7 23Na
		656.2751	-2.2	-3.4	12.0	-1.$	n/a	1.#R	12C30 13C 1H42 14N3 16O11 23Na
		656.2706	2.3	3.5	12.5	-1.$	n/a	1.#R	12C29 13C2 1H41 14N3 16O11 23Na
		656.2705	2.4	3.7	20.5	-1.$	n/a	1.#R	12C39 13C 1H40 16O7 23Na
		656.2701	2.8	4.3	30.5	-1.$	n/a	1.#R	12C42 13C2 1H33 14N5 23Na
		656.2759	-3.0	-4.6	29.5	-1.$	n/a	1.#R	12C45 13C 1H36 14N2 16O 23Na
		656.2759	-3.0	-4.6	21.5	-1.$	n/a	1.#R	12C35 13C2 1H37 14N5 16O5 23Na
		656.2696	3.3	5.0	16.5	-1.$	n/a	1.#R	12C33 1H39 14N5 16O8 23Na
657.2695	13.20	657.2699	-0.4	-0.6	16.5	-1.$	n/a	1.#R	12C33 13C2 1H40 14N2 16O9 23Na
		657.2689	0.6	0.9	20.5	-1.$	n/a	1.#R	12C37 1H38 14N4 16O6 23Na
		657.2702	-0.7	-1.1	20.0	-1.$	n/a	1.#R	12C39 1H40 14N 16O7 23Na
		657.2703	-0.8	-1.2	12.0	-1.$	n/a	1.#R	12C29 13C 1H41 14N4 16O11 23Na
		657.2685	1.0	1.5	17.0	-1.$	n/a	1.#R	12C31 13C2 1H38 14N5 16O8 23Na
		657.2685	1.0	1.5	25.0	-1.$	n/a	1.#R	12C41 13C 1H37 14N2 16O4 23Na
		657.2680	1.5	2.3	29.5	-1.$	n/a	1.#R	12C45 13C2 1H36 16O2 23Na
		657.2711	-1.6	-2.4	29.5	-1.$	n/a	1.#R	12C44 13C 1H35 14N3 16O 23Na
		657.2676	1.9	2.9	15.5	-1.$	n/a	1.#R	12C36 1H42 16O10 23Na
		657.2716	-2.1	-3.2	25.0	-1.$	n/a	1.#R	12C40 1H36 14N5 16O3 23Na
		657.2717	-2.2	-3.3	11.5	-1.$	n/a	1.#R	12C31 13C 1H43 14N 16O12 23Na
		657.2672	2.3	3.5	12.0	-1.$	n/a	1.#R	12C30 13C2 1H42 14N 16O12 23Na
		657.2671	2.4	3.7	25.5	-1.$	n/a	1.#R	12C39 13C 1H35 14N5 16O3 23Na
		657.2667	2.8	4.3	30.0	-1.$	n/a	1.#R	12C43 13C2 1H34 14N3 16O 23Na
		657.2725	-3.0	-4.6	29.0	-1.$	n/a	1.#R	12C46 13C 1H37 16O2 23Na
		657.2725	-3.0	-4.6	21.0	-1.$	n/a	1.#R	12C36 13C2 1H38 14N3 16O6 23Na
		657.2662	3.3	5.0	16.0	-1.$	n/a	1.#R	12C34 1H40 14N3 16O9 23Na

Figure S86. HR-MS-ES (m/z) for compound **1** diastereomer **A** (sample label: SD-1-42-spot3/A): $[M+Na]^+$ calculated for $C_{31}H_{42}N_3O_{11}Na$ 655.2717 was found to be 655.2697 (-3.1 ppm, RA 100%), for $^{12}C_{30}{}^{13}CH_{42}N_3O_{11}Na$ 656.2751, it was found to be 656.2729 (-3.4 ppm, RA 33%)

Multiple Mass Analysis: 3 mass(es) processed
Tolerance = 2.0 PPM / DBE: min = -1.5, max = 50.0
Element prediction: Off
Number of isotope peaks used for i-FIT = 3

Monoisotopic Mass, Odd and Even Electron Ions
2589434 formula(e) evaluated with 22 results within limits (up to 50 best isotopic matches for each mass)
Elements Used:
12C: 0-50 13C: 0-2 1H: 0-100 14N: 0-5 16O: 0-12 23Na: 1-1
61970 SD-1-42SPOT4 58 (0.142) AM (Med,5, Ar,10000.0,0.00,0.70); Cm (58:304)
TOF MS ES+

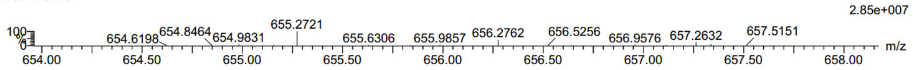

Minimum:	10.00				-1.5				
Maximum:	100.00		5.0	2.0	50.0				

Mass	RA	Calc. Mass	mDa	PPM	DBE	i-FIT	Norm	Conf(%)	Formula
655.2721	100.00	655.2721	0.0	0.0	26.0	-1.$	n/a	1.#R	12C40 13C2 1H36 14N3 16O3 23Na
		655.2717	0.4	0.6	12.0	-1.$	n/a	1.#R	12C31 1H42 14N3 16O11 23Na
		655.2725	-0.4	-0.6	29.5	-1.$	n/a	1.#R	12C46 1H36 14N2 16O 23Na
		655.2726	-0.5	-0.8	21.5	-1.$	n/a	1.#R	12C36 13C 1H37 14N5 16O5 23Na
		655.2713	0.8	1.2	16.5	-1.$	n/a	1.#R	12C35 13C 1H41 14N 16O9 23Na
		655.2730	-0.9	-1.4	11.5	-1.$	n/a	1.#R	12C33 1H44 16O12 23Na
		655.2712	0.9	1.4	30.0	-1.$	n/a	1.#R	12C44 1H34 14N5 23Na
656.2762	37.02	656.2763	-0.1	-0.2	25.0	-1.$	n/a	1.#R	12C41 1H37 14N4 16O3 23Na
		656.2764	-0.2	-0.3	11.5	-1.$	n/a	1.#R	12C32 13C 1H44 16O12 23Na
		656.2759	0.3	0.5	29.5	-1.$	n/a	1.#R	12C45 13C 1H36 14N2 16O 23Na
		656.2759	0.3	0.5	21.5	-1.$	n/a	1.#R	12C35 13C2 1H37 14N5 16O5 23Na
		656.2751	1.1	1.7	12.0	-1.$	n/a	1.#R	12C30 13C 1H42 14N3 16O11 23Na
		656.2773	-1.1	-1.7	21.0	-1.$	n/a	1.#R	12C37 13C2 1H39 14N2 16O6 23Na
		656.2750	1.2	1.8	20.0	-1.$	n/a	1.#R	12C40 1H41 16O7 23Na
657.2632	20.68	657.2631	0.1	0.2	16.0	-1.$	n/a	1.#R	12C35 13C 1H41 16O10 23Na
		657.2630	0.2	0.3	29.5	-1.$	n/a	1.#R	12C44 1H34 14N4 16O 23Na
		657.2635	-0.3	-0.5	11.5	-1.$	n/a	1.#R	12C31 1H42 14N2 16O12 23Na
		657.2626	0.6	0.9	26.0	-1.$	n/a	1.#R	12C38 13C2 1H34 14N5 16O3 23Na
		657.2640	-0.8	-1.2	25.5	-1.$	n/a	1.#R	12C40 13C2 1H36 14N2 16O4 23Na
		657.2622	1.0	1.5	12.0	-1.$	n/a	1.#R	12C29 1H40 14N5 16O11 23Na
		657.2644	-1.2	-1.8	29.0	-1.$	n/a	1.#R	12C46 1H36 14N 16O2 23Na
		657.2644	-1.2	-1.8	21.0	-1.$	n/a	1.#R	12C36 13C 1H37 14N4 16O6 23Na

Figure S87. HR-MS-ES (m/z) for compound **1** diastereomer **B** (sample label: SD-1-42-spot4/B): $[M+Na]^+$ calculated for $C_{31}H_{42}N_3O_{11}Na$ 655.2717 was found to be 655.2721 (0.6 ppm, RA 100%), for $^{12}C_{30}{}^{13}CH_{42}N_3O_{11}Na$ 656.2751, it was found to be 656.2762 (1.7 ppm, RA 37%).

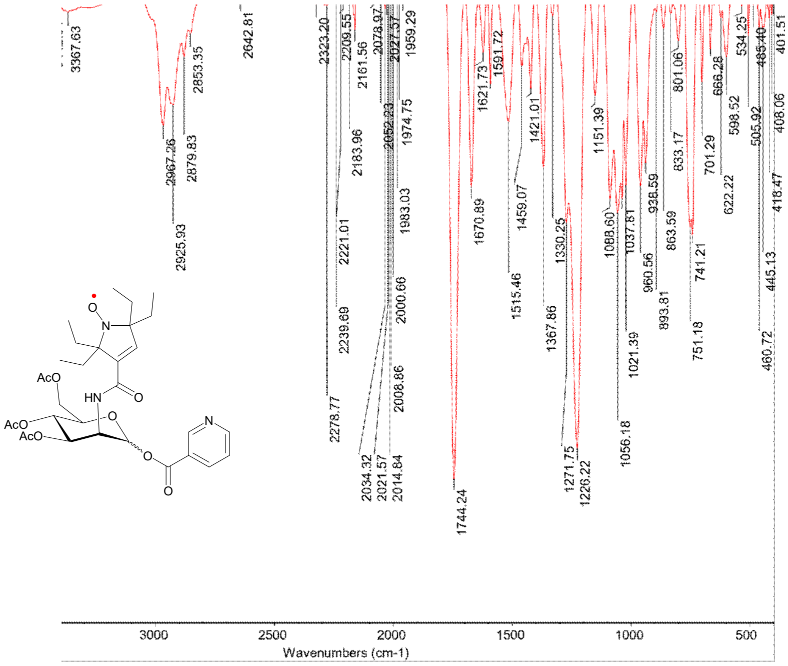

Figure S88. IR spectrum for compound **1** (sample label:SD-1-42-spot3/A).

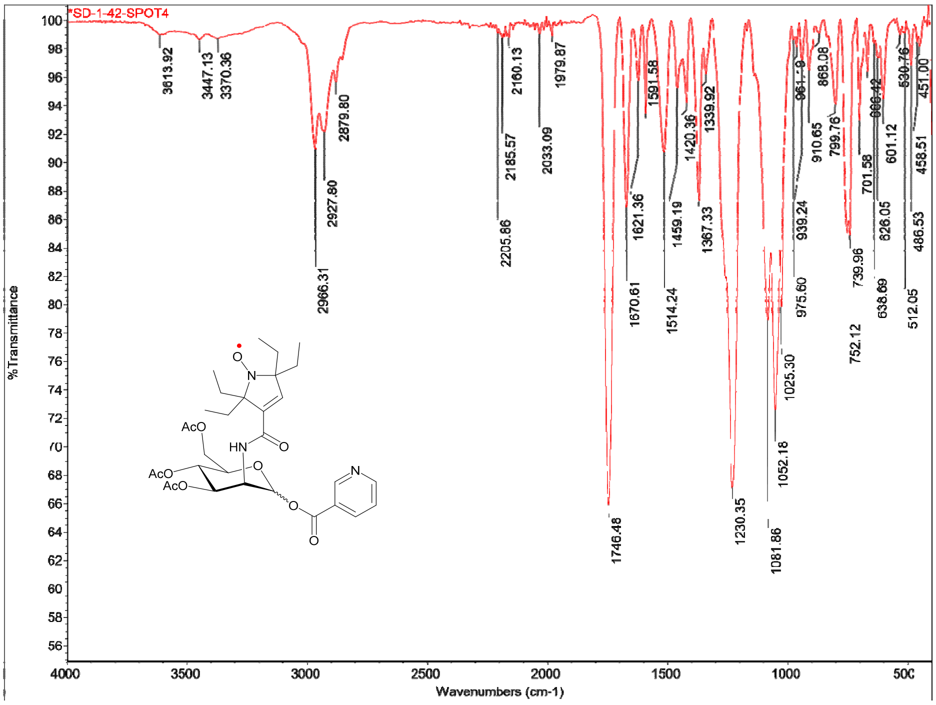

Figure S89. IR spectrum for compound **1** (Sample label: SD-1-42-spot4/B).

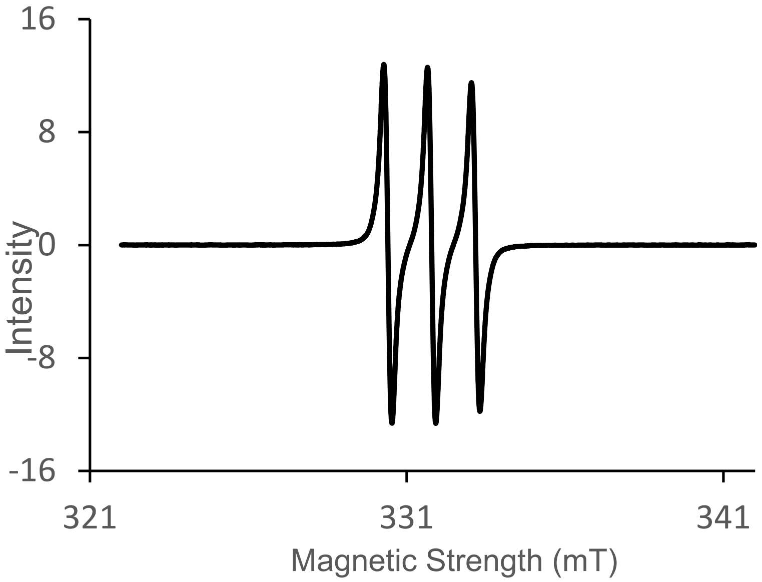

Figure S90. EPR spectrum of compound **1** (sample label: SD-1-17-spotA, 0.829 mM in dichloromethane/toluene (1:4), frequency = 9.317097 GHz, modulation amplitude = 0.1 mT, attenuation = 30 dB, receiver gain = 30 dB, central field = 332 mT, sweep width = 20 mT, sweep time = 10 s, and number of scans = 2).

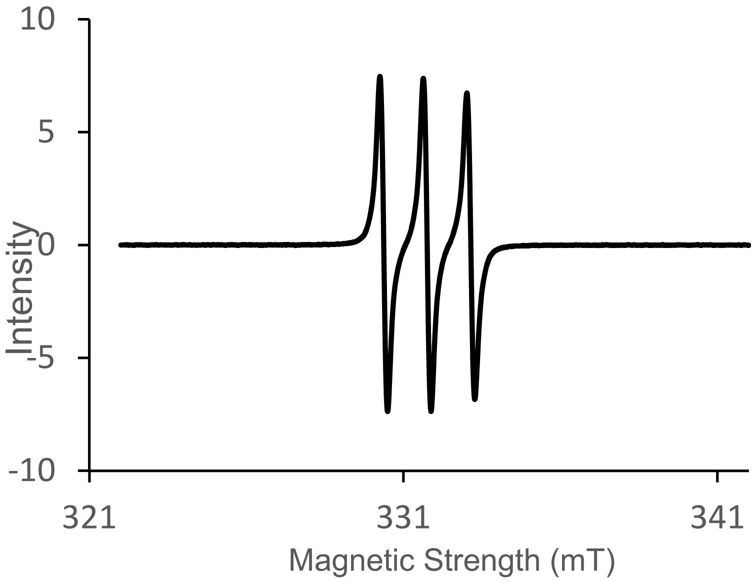

Figure S91. EPR spectrum of compound **1** (sample label: SD-1-17-spotB, 0.438 mM in dichloromethane/toluene (1:4), frequency = 9.316201 GHz, modulation amplitude = 0.05 mT, attenuation = 25 dB, receiver gain = 30 dB, center field = 332 mT, sweep width = 20 mT, sweep time = 10 s, and number of scans = 2).

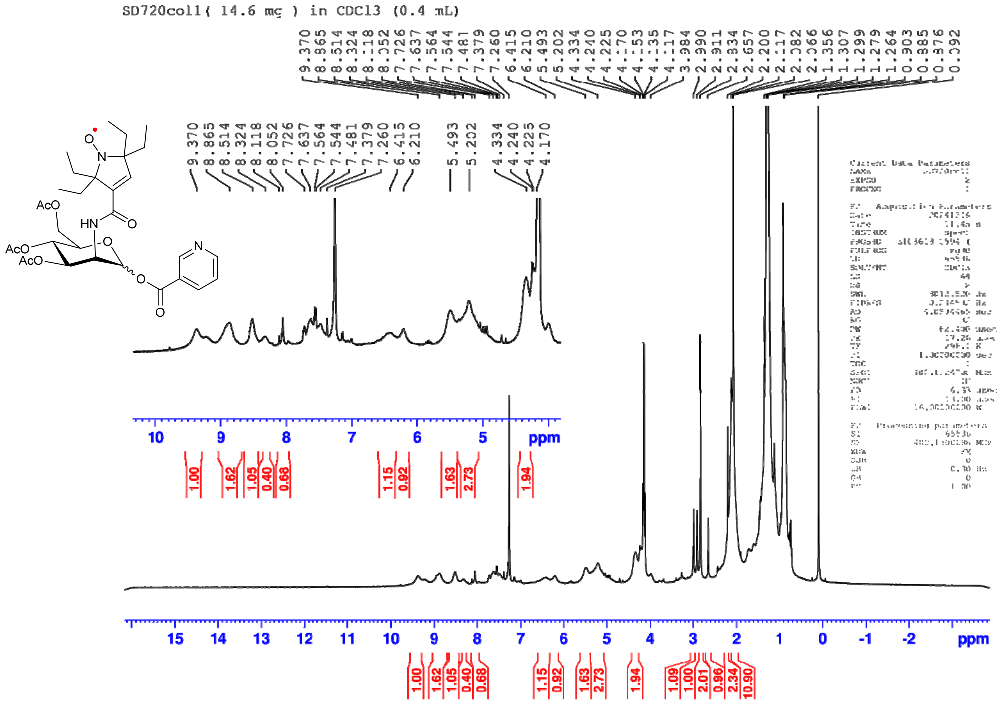

Figure S92. ^{1}H NMR of compound **1** (sample label: SD720col1, 400 MHz, and 0.046 M in $CDCl_3$).

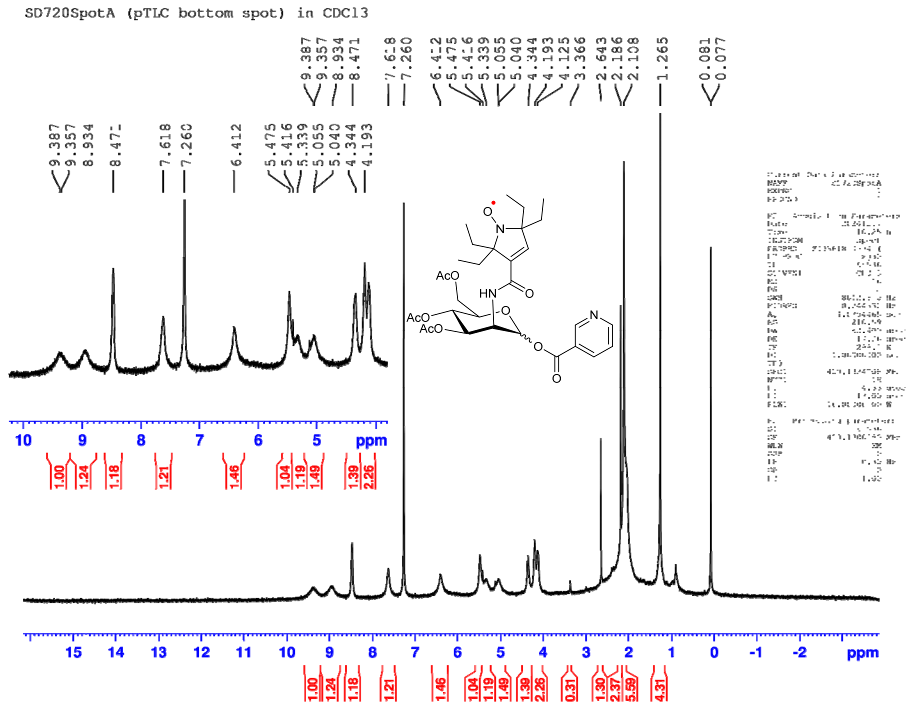

Figure S93. ^{1}H NMR of compound **1** (sample label: SD720spotA, 400 MHz, and 0.010 M in $CDCl_3$).

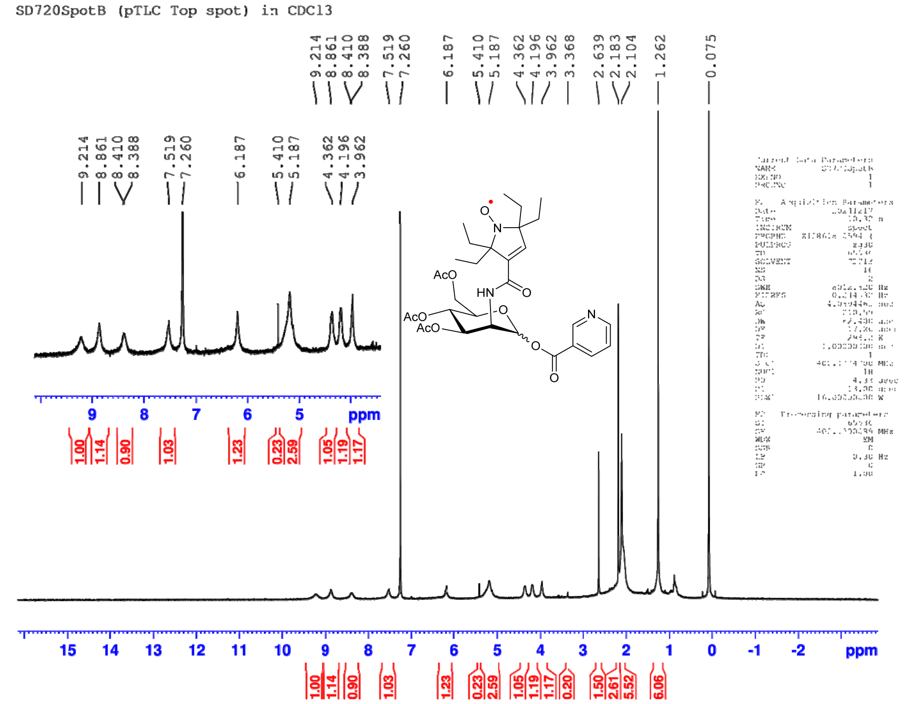

Figure S94. ^{1}H NMR of compound **1** (sample label: SD720spotB, 400 MHz, and 0.0075 M in $CDCl_3$).

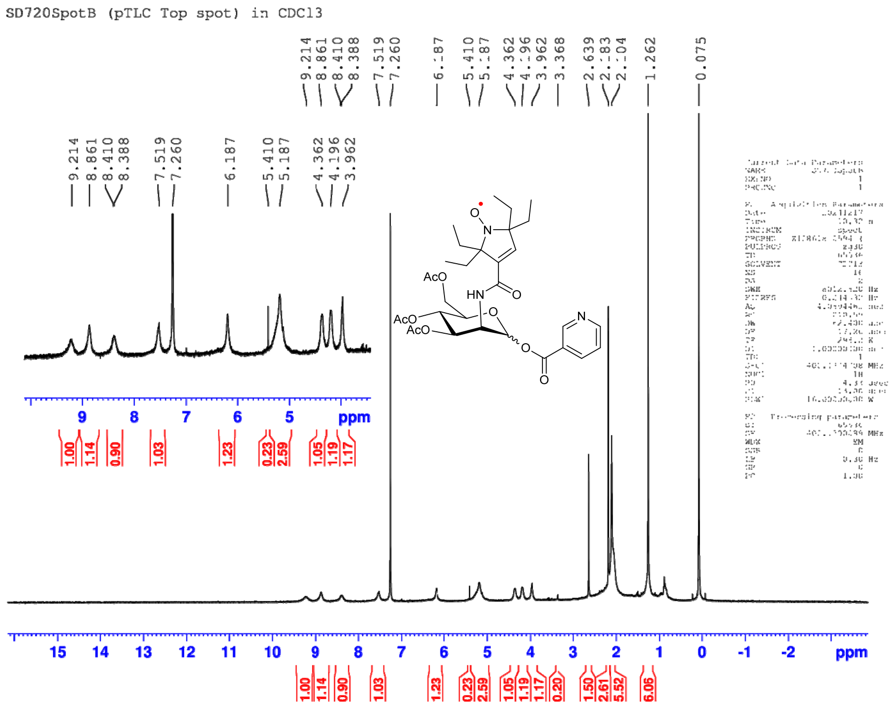

Figure S95. ^{1}H NMR of compound **1** (sample label: SD720spotA, 400 MHz, and 0.010 M in $CDCl_3$).

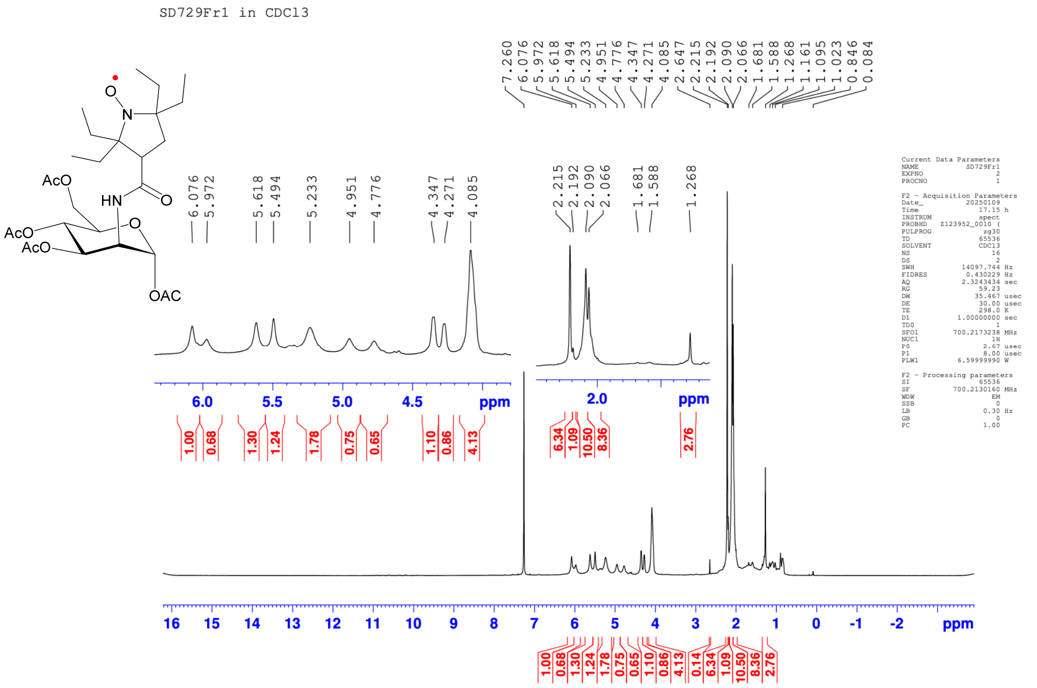

Figure S96. ^{1}H NMR of compound **2a** (sample label: SD729Fr1, 700 MHz, and 0.018 M in $CDCl_3$).

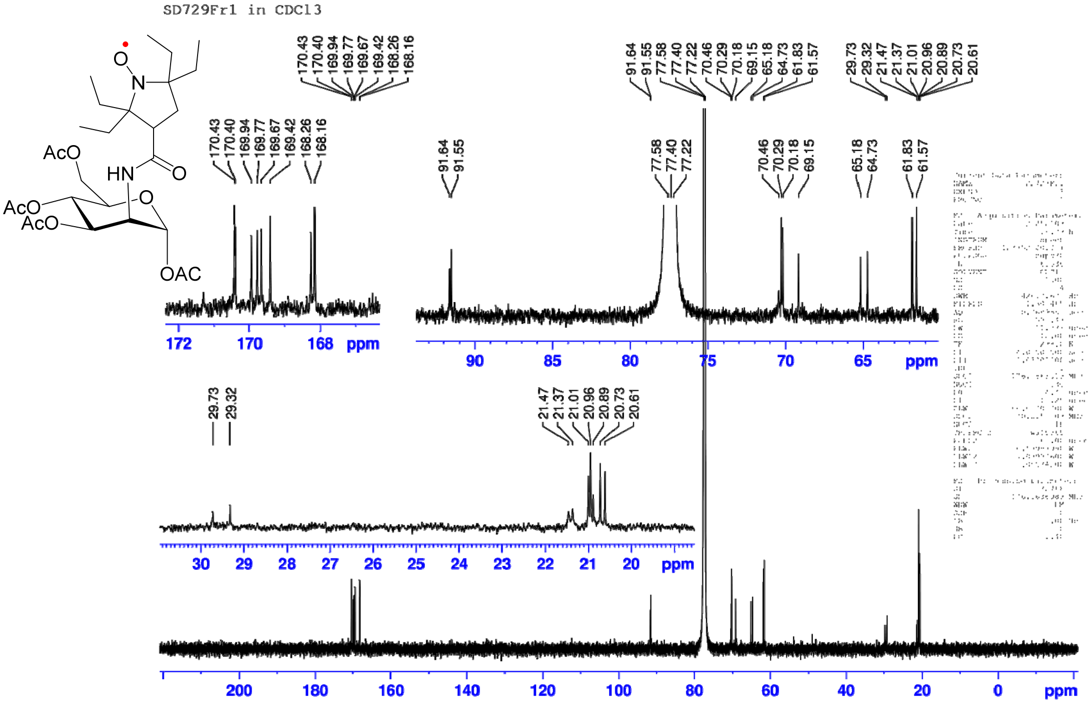

Figure S97. ^{13}C NMR of compound **2a** (sample label: SD729Fr1, 700 MHz, and 0.018 M in $CDCl_3$).

Analysis Name	D:\Kurt's stuff\Kurt's Data\10217 SD729Fr1_000001.d		
Method	Shim 10_23_24	Operator	
Sample Name		Instrument	solariX XR
Comment			

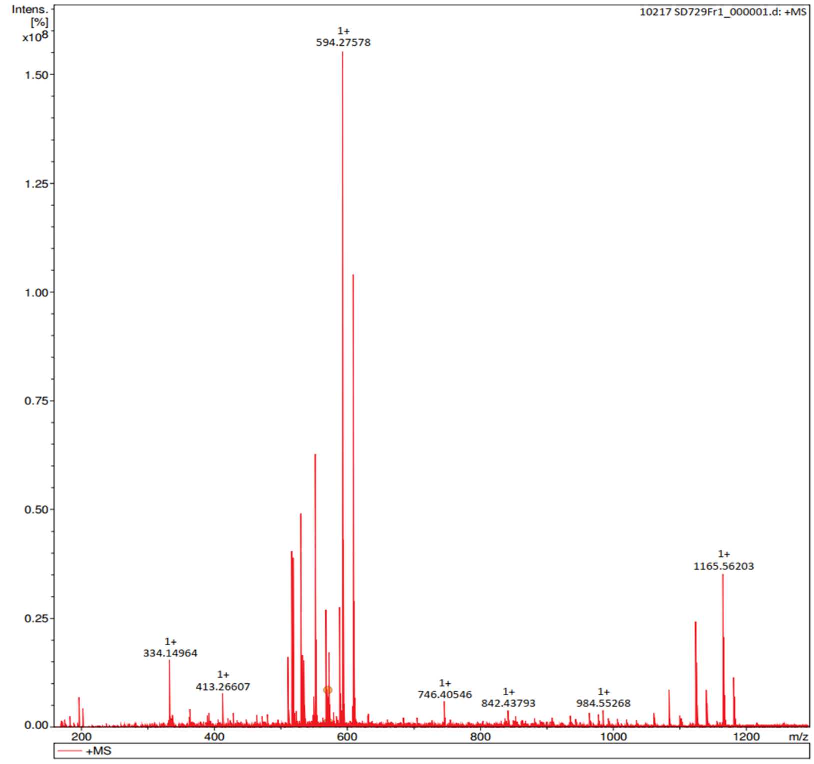

Meas. m/z	#	Ion Formula	Score	m/z	err [ppm]	Mean err [ppm]	mSigma	rdb	e^- Conf	N-Rule
569.363593	1	C26H53N2O11	83.65	569.364387	1.4	3.0	16.7	2.0	even	ok
	2	C24H51N5O10	100.00	569.363044	-1.0	0.6	24.6	2.5	odd	ok
	3	C25H47N9O6	69.86	569.364382	1.4	1.4	34.4	7.5	odd	ok
	4	C23H45N12O5	63.99	569.363039	-1.0	-0.6	42.1	8.0	even	ok
	5	C24H41N16O	56.45	569.364376	1.4	1.5	42.4	13.0	even	ok
	6	C22H39N19	47.95	569.363034	-1.0	-0.5	51.8	13.5	odd	ok
	7	C39H45N4	53.84	569.363874	0.5	2.4	52.3	20.0	even	ok
	8	C38H49O4	26.82	569.362536	-1.9	0.2	54.7	15.0	even	ok
594.275783	1	C25H41N5NaO10	61.82	594.274563	-2.1	-1.7	13.3	8.0	even	ok
	2	C26H37N9NaO6	100.00	594.275901	0.2	-0.7	17.5	13.0	even	ok
	3	C27H43N2NaO11	76.25	594.275906	0.2	0.3	19.7	7.5	odd	ok
	4	C24H35N12NaO5	52.61	594.274558	-2.1	-2.7	21.0	13.5	odd	ok
	5	C25H31N16NaO	82.72	594.275895	0.2	-0.7	26.3	18.5	odd	ok
	6	C23H29N19Na	39.34	594.274553	-2.1	-2.5	33.3	19.0	even	ok
	7	C40H35N4Na	15.79	594.275393	-0.7	-0.8	75.8	25.5	odd	ok
	8	C42H37NNaO	3.83	594.276736	1.6	1.2	97.1	25.0	even	ok
610.249734	1	C25H41KN5O10	59.95	610.248500	-2.0	-1.8	12.8	8.0	even	ok
	2	C27H43KN2O11	99.86	610.249843	0.2	0.2	16.1	7.5	odd	ok
	3	C26H37KN9O6	100.00	610.249838	0.2	0.3	16.2	13.0	even	ok
	4	C24H35KN12O5	52.51	610.248495	-2.0	-1.6	19.2	13.5	odd	ok
	5	C25H31KN16O	69.74	610.249833	0.2	-0.0	25.8	18.5	odd	ok
	6	C23H29KN19	33.47	610.248490	-2.0	-1.9	31.9	19.0	even	ok
	7	C40H35KN4	23.00	610.249330	-0.7	-0.2	64.7	25.5	odd	ok
	8	C42H37KNO	8.49	610.250673	1.5	1.9	79.7	25.0	even	ok
1165.562032	1	C51H88N3NaO25	45.42	1165.559911	-1.8	-2.2	6.4	9.5	odd	ok
	2	C50H82N10NaO20	44.79	1165.559906	-1.8	-2.4	6.9	15.0	even	ok
	3	C49H76N17NaO15	41.30	1165.559901	-1.8	-2.7	11.1	20.5	odd	ok
	4	C53H90NaO26	100.00	1165.561254	-0.7	-1.0	11.4	9.0	even	ok
	5	C52H84N7NaO21	96.15	1165.561248	-0.7	-1.2	13.3	14.5	odd	ok
	6	C48H70N24NaO10	37.10	1165.559895	-1.8	-2.9	16.4	26.0	even	ok
	7	C51H78N14NaO16	88.87	1165.561243	-0.7	-1.4	17.1	20.0	even	ok
	8	C54H86N4NaO22	94.35	1165.562591	0.5	0.1	19.9	14.0	even	ok
	9	C50H72N21NaO11	80.16	1165.561238	-0.7	-1.6	21.8	25.5	odd	ok
	10	C53H80N11NaO17	87.46	1165.562586	0.5	-0.1	23.4	19.5	odd	ok
	11	C47H64N31NaO5	24.93	1165.559890	-1.8	-2.9	24.3	31.5	odd	ok
	12	C56H88NNaO23	35.64	1165.563934	1.6	1.4	26.5	13.5	odd	ok
	13	C49H66N28NaO6	71.08	1165.561233	-0.7	-1.9	27.0	31.0	even	ok
	14	C52H74N18NaO12	79.30	1165.562581	0.5	-0.3	27.8	25.0	even	ok
	15	C55H82N8NaO18	32.95	1165.563929	1.6	1.1	30.0	19.0	even	ok
	16	C46H58N38Na	21.39	1165.559885	-1.8	-3.1	30.6	37.0	even	ok
	17	C51H68N25NaO7	70.64	1165.562575	0.5	-0.6	32.7	30.5	odd	ok
	18	C54H76N15NaO13	29.84	1165.563923	1.6	0.9	34.1	24.5	odd	ok
	19	C48H60N35NaO	45.31	1165.561227	-0.7	-1.8	36.1	36.5	odd	ok
	20	C53H70N22NaO8	26.56	1165.563918	1.6	0.7	38.7	30.0	even	ok
	21	C50H62N32NaO2	44.05	1165.562570	0.5	-0.5	42.1	36.0	even	ok
	22	C52H64N29NaO3	23.28	1165.563913	1.6	0.5	43.6	35.5	odd	ok

Page 1

Figure S98. HR-MS-ESI (m/z) for compound **2a** (sample label: SD729Fr1): $[M+Na]^+$ calculated for $C_{27}H_{43}N_2O_{11}Na$ 594.275906 was found to be 594.275783 (0.2 ppm), and $[2M+Na]^+$ calculated for $C_{54}H_{86}N_4O_{22}Na$ 1165.562591 was found to be 1165.56203 (0.5 ppm).

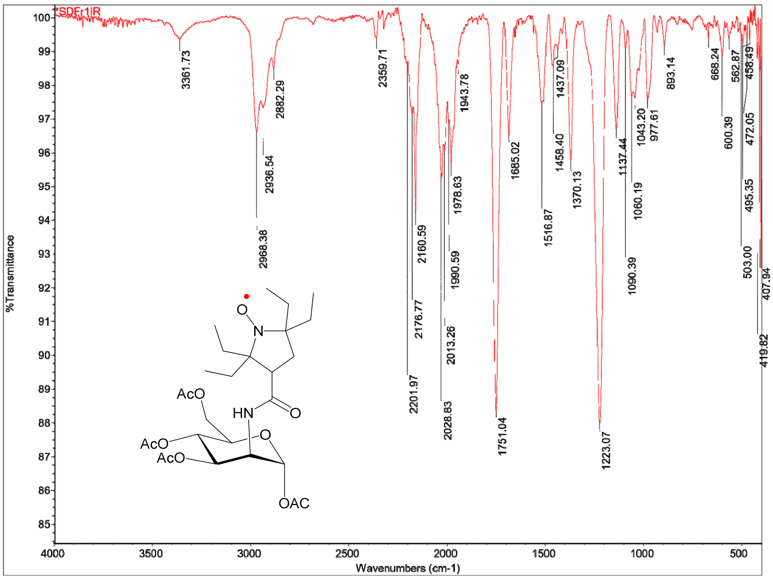

Figure S99. IR spectrum for compound **2a** (sample label: SD729Fr1).

Supporting References

S1. Sampathkumar, S.-G.; Li, A. V.; Yarema, K. J. Synthesis of Non-Natural ManNAcAnalogs for the Expression of Thiols on Cell-Surface Sialic Acids. *Nat Protoc* **2006**, *1* (5), 2377–2385.

S2. Ghosh, A. K.; Shahabi, D. Synthesis of Amide Derivatives for Electron Deficient Amines and Functionalized Carboxylic Acids Using EDC and DMAP and a Catalytic Amount of HOBt as the Coupling Reagents. *Tetrahedron Letters* **2021**, *63*, 152719.

S3. Wang, Y.; Paletta, J. T.; Berg, K.; Reinhart, E.; Rajca, S.; Rajca, A. Synthesis of Unnatural Amino Acids Functionalized with Sterically Shielded Pyrroline Nitroxides. *Org. Lett.* **2014**, *16* (20), 5298–5300.

S4. Hattie, M.; Stubbs, K. A. Generalising a Simple Methodology for the Regioselective Anomeric Deacetylation of Carbohydrates. *ChemistrySelect* **2020**, *5* (2), 875–877.

S5. Shajahan, A.; Parashar, S.; Goswami, S.; Ahmed, S. M.; Nagarajan, P.; Sampathkumar, S.-G. Carbohydrate–Neuroactive Hybrid Strategy for Metabolic Glycan Engineering of the Central Nervous System in Vivo. *J. Am. Chem. Soc.* **2017**, *139* (2), 693–700.
